# Mechanism and Origin of Regioselectivity in the Phosphine-Catalyzed Heine Reaction

**DOI:** 10.1021/acs.joc.5c00416

**Published:** 2025-05-07

**Authors:** Sebastián Gallardo-Fuentes, Lucas Lodeiro, Israel Fernández

**Affiliations:** † Instituto de Química, Facultad de Ciencias, Pontificia Universidad Católica de Valparaíso, Avenida Universidad 330, Curauma, Valparaíso 2373223, Chile; ‡ Departamento de Química, Facultad de Ciencias, 117433 Universidad de Chile, Las Palmeras 3425, Ñuñoa 7800003, Santiago, Chile; § Departamento de Química Orgánica I and Centro de Innovación en Química Avanzada (ORFEO−CINQA), Facultad de Ciencias Químicas, 16734 Universidad Complutense de Madrid, 28040 Madrid, Spain

## Abstract

Herein, we present a comprehensive computational study of the reaction mechanism and regioselectivity patterns of the phosphine-catalyzed Heine reaction involving *N*-benzoylaziridines. Density functional theory (DFT) calculations reveal that the regioselectivity of the process takes place under kinetic control, favoring the formation of the corresponding 4-substituted oxazoline derivatives. Conformational analysis indicates that the predominantly populated ground-state conformation of aziridine does not represent the kinetically active species. Exploration of the conformational space in the transition state (TS) region shows that the preferred pathway for the nucleophilic ring-opening process involves a TS structure where the benzoyl moiety adopts a nearly coplanar arrangement. Furthermore, the main factors controlling the observed regioselectivity as well as the impact of substituents on the reactivity, are quantitatively rationalized using the activation strain model of reactivity in combination with energy decomposition analysis.

## Graphical abstract

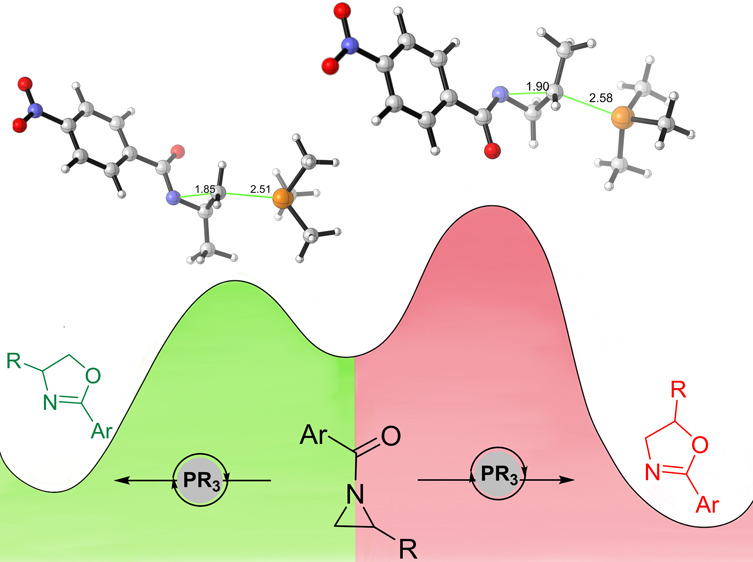

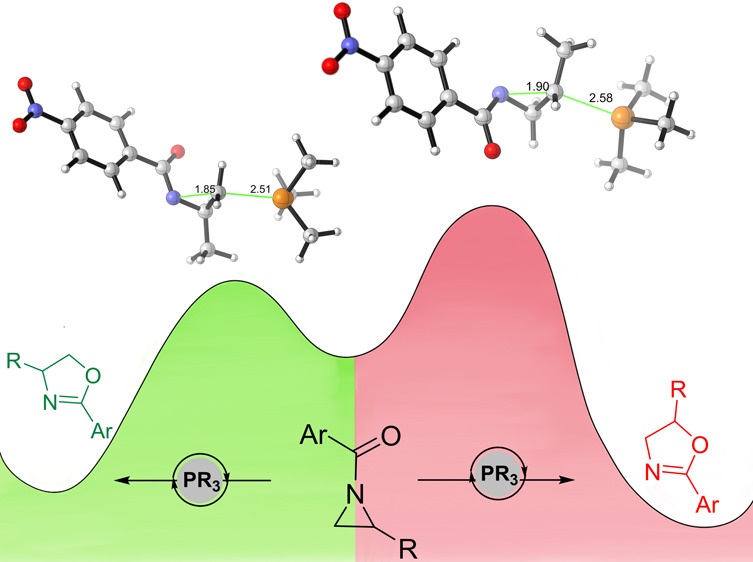

## Introduction

The field of organic synthesis relies on the strategic use of molecular scaffolds with unique reactivity and synthetic potential. Among them, aziridines are particularly valuable building blocks due to their broad applications in the preparation of nitrogen-containing biologically active structures and natural products.^1^ The strained three-membered structure of aziridines is prone to engage in nucleophilic ring-opening reactions by a range of nucleophiles,^2^ thereby serving as versatile synthons in organic synthesis for increasing molecular complexity. For instance, when aziridines undergo ring-opening with indole as a nucleophilic reagent, they yield tryptamine derivatives, species commonly found in various biologically active natural products.^3^ Furthermore, in the past few years, several organocatalytic systems, such as amines,^4^ *N*-heterocyclic carbenes,^5^ and phosphines,^6^ have been employed for the intermolecular activation of aziridines with various nucleophiles.

Even though the nucleophilic ring-opening reaction of aziridines primarily generates functionalized building blocks, the use of Lewis bases to catalyze the rearrangement of the aziridine ring has also been described. In this regard, Heine and co-workers were pioneers in utilizing *N*-acylaziridines as synthetic precursors for the production of oxazolines under Lewis base catalysis.^7^ As oxazolines are valuable organic synthons and potential ligands for transition-metal catalysis, different methods to achieve enantioselective versions of the Heine reaction have also been reported.^8^ Inspired by Heine’s seminal work, Morgan and co-workers disclosed an elegant and unprecedented phosphine-catalyzed ring rearrangement of *N-*acylaziridines into oxazoline derivatives (Scheme 1).^9^ These authors demonstrated the utility of electron-rich phosphines to regioselectively catalyze the C–N ring-opening of *N*-acylaziridines. Although detailed mechanistic studies on this phosphine-catalyzed Heine reaction have not been undertaken, the accepted mechanism involves two consecutive S_N_2 processes (Scheme 1).^9^ First, the nucleophilic ring-opening stage proceeds through an S_N_2-type mechanism involving the nucleophilic attack on either the C2 or C3 aziridine carbon atom with the ring nitrogen acting as the leaving group. Next, the catalytically generated phosphonium intermediates (**int1-C2** or **int1-C3**) can engage in an intramolecular nucleophilic attack through a *5-exo-tet* ^10^ cyclization with concomitant release of the phosphine catalyst, affording the regioisomeric oxazoline derivatives **2** and **3**, as illustrated in Scheme 1. Remarkably, the **2:3** ratio varies depending on the phosphine used as the catalyst, with oxazoline **3** consistently being the major product obtained.^9^ However, the reasons behind this good to almost exclusive selectivity (>20:1 when using a bulky phosphine) are essentially unknown.

**Scheme 1:**
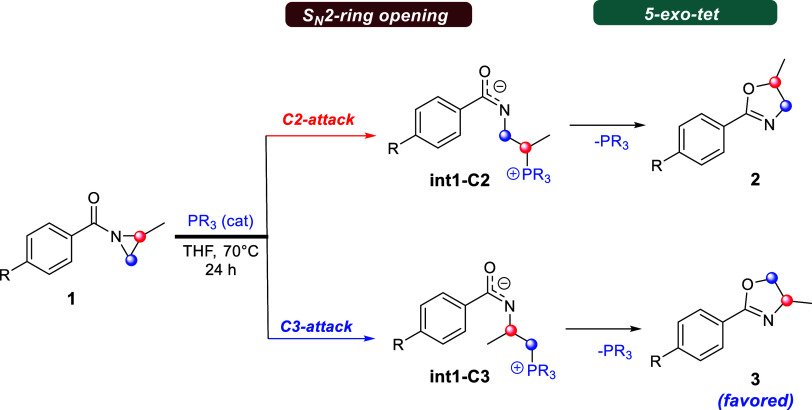
Proposed Mechanism for the Phosphine-Catalyzed Heine Reaction

Despite the number of theoretical studies devoted to the nucleophilic ring-opening reaction of aziridines,^11^ the mechanism of the phosphine-catalyzed Heine reaction has not been investigated in detail so far. This prompted us to perform a comprehensive Density Functional Theory (DFT) study to unravel the underlying factors controlling the reactivity and selectivity patterns in this transformation. To this end, state-of-the-art computational methods, namely, the Activation Strain Model (ASM)^12^ of chemical reactivity (also called the distortion-interaction model)^13^ in combination with the Energy Decomposition Analysis (EDA)^14^ method will be applied. This approach was chosen because it has greatly contributed to our current understanding of fundamental reactions in organic and organometallic chemistry,^15^ including organocatalyzed transformations.^16^

## Results and Discussion

In their original experimental study, Morgan and co-workers highlighted that the nucleophilic ring-opening reaction of *N*-acyl aziridines occurs regioselectively at the less hindered aziridine carbon atom. Moreover, they demonstrated that the substitution patterns on the benzoyl moiety and the nature of the phosphine catalyst are essential for achieving high reaction yields and regioselectivity.^9^ To better understand the reactivity patterns and regioselectivities in this phosphine-catalyzed Heine reaction, we conducted a computational study at the M06-2*X*/6-31+G­(d,p) level.^17^ For this purpose, we selected the PCyPh_2_-catalyzed Heine reaction of methyl-substituted *N*-benzoyl aziridine **1a** as a model reaction (see Scheme 2), which favors the formation of the oxazoline **3a** over **2a** (ratio 91:9).

**Scheme 2:**
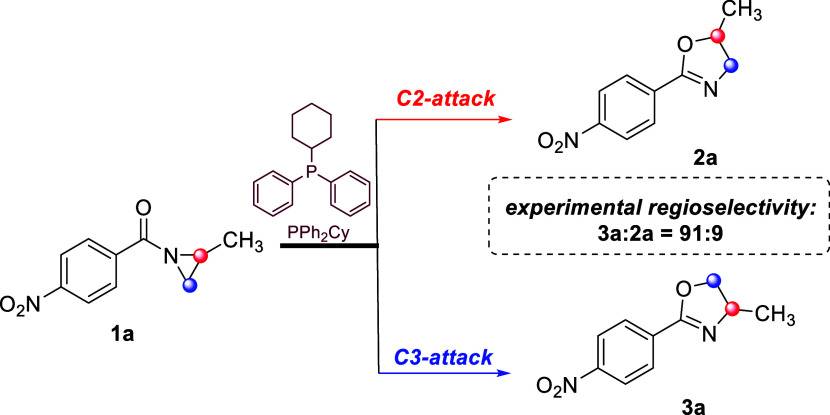
Regioselectivity in the PCyPh_2_-Catalyzed Heine Reaction Involving *N*-Benzoylaziridine **1a** ^a^

a Cy = cyclohexyl group.

## Conformational Analysis

Before investigating the ring-opening reaction, we first conducted a conformational analysis of aziridines **1a**–**d** in their ground state. It is well-documented that *N*-substituted aziridines exist as an equilibrating mixture of two invertomers (*cis* and *trans*) due to nitrogen inversion.^18^ Furthermore, rotation around the C–N bond in *N*-benzoyl-2-methylaziridines can result in two conformers for each invertomer, namely one with the aryl fragment pointing toward the aziridine ring and another where the aryl group is oriented outward from the aziridine moiety, as depicted in Scheme 3. All four conformations have been characterized as minima at the SMD­(THF)-M06-2*X*/6-31+G­(d,p) level, showing a conformational preference for the ***trans-in*** arrangement, regardless of the nature of substituents on the aryl ring (see Table 1). The DFT-optimized geometries for all these main conformers are presented in the Supporting Information (SI, Figures S1–S4). Despite that, the computed energy differences between the conformers are <2 kcal/mol. This, together with the computed rather low interconversion barriers (Δ*G* ^‡^ = 5.9 and 6.0 kcal/mol for the ***trans-in→cis-in*** and ***trans-in→trans-out*** processes, respectively, X = NO_2_), suggest a rapid interconversion between all species at the temperature used in the experiments (70 °C).

**Scheme 3:**
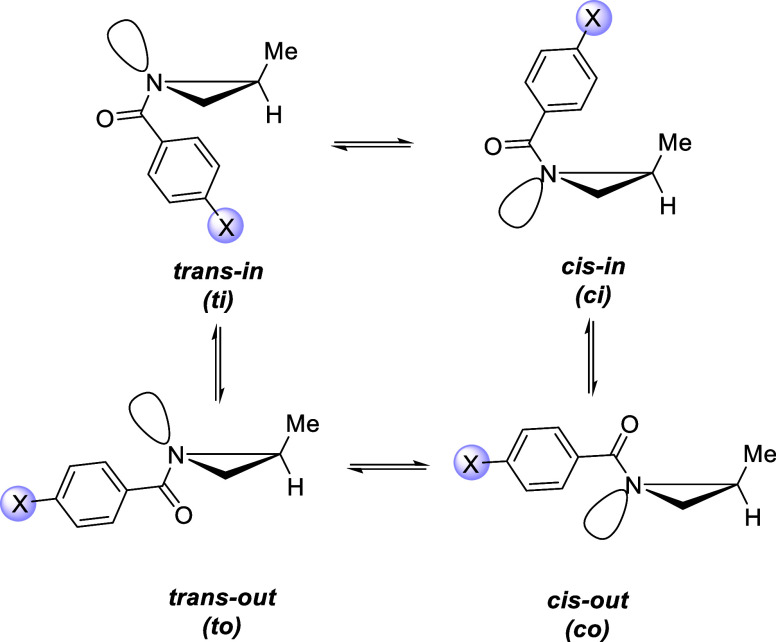
Possible Conformers in *N*-Benzoyl-2-methylaziridines

**Table 1:** Relative Gibbs Free Energies (in kcal/mol) for the Main Conformers of *N*-Benzoyl-2-methyl Aziridines **1a**–**d** Optimized at the SMD­(THF)-M06-2*X*/6-31+G­(d,p) Level

compound	**substituent (X)**	***trans-in***	***trans-out***	***cis-in***	***cis-out***
**1a**	**NO**_**2**_	0.0	0.4	0.5	1.4
**1b**	**Cl**	0.0	0.3	0.5	1.6
**1c**	**H**	0.0	0.4	0.5	1.8
**1d**	**OMe**	0.0	0.3	0.6	2.0

As suggested in Scheme 1, the phosphine-catalyzed Heine reaction initiates through an S_N_2-type ring-opening mechanism, where the phosphine catalyst undergoes a nucleophilic attack on either the C2 or C3 aziridine carbon atom. We have explored all the possible ring-opening reactions involving the different conformers of aziridine **1a**. Even though the conformational analysis revealed that the ***trans-in*** arrangement is the lowest energy conformer, the nucleophilic ring-opening mechanism via the C3 attack is unfeasible at this conformation according to the computed rather high activation barrier (Δ*G* ^‡^ = 41.8 kcal/mol, Figure 1). Similar high barriers were computed for the ***cis-out*** and ***cis-in*** conformers. At variance, we found a much more feasible ring-opening reaction for conformer ***trans-out***, which exhibits a barrier of 34.6 kcal/mol (through transition state **TS1aC3-** ***to***, Figure 1). A similar exploration of the potential energy surface for the C2-attack pathway indicates that the lowest energy TS conformation also involves the ***trans-out*** arrangement (Δ*G* ^‡^ = 36.0 kcal/mol, Figure 2). It is important to note that the same reactivity pattern was observed when modeling the corresponding TS conformations using a structurally simpler catalyst, namely trimethylphosphine (see Figures S5 and S6 in SI). These results suggest that the kinetic preference toward the **TS1aC3-** ***to*** conformation is determined by the spatial arrangement adopted by the substrate in the TS region rather than by the nature of the catalyst.

**Figure 1:**
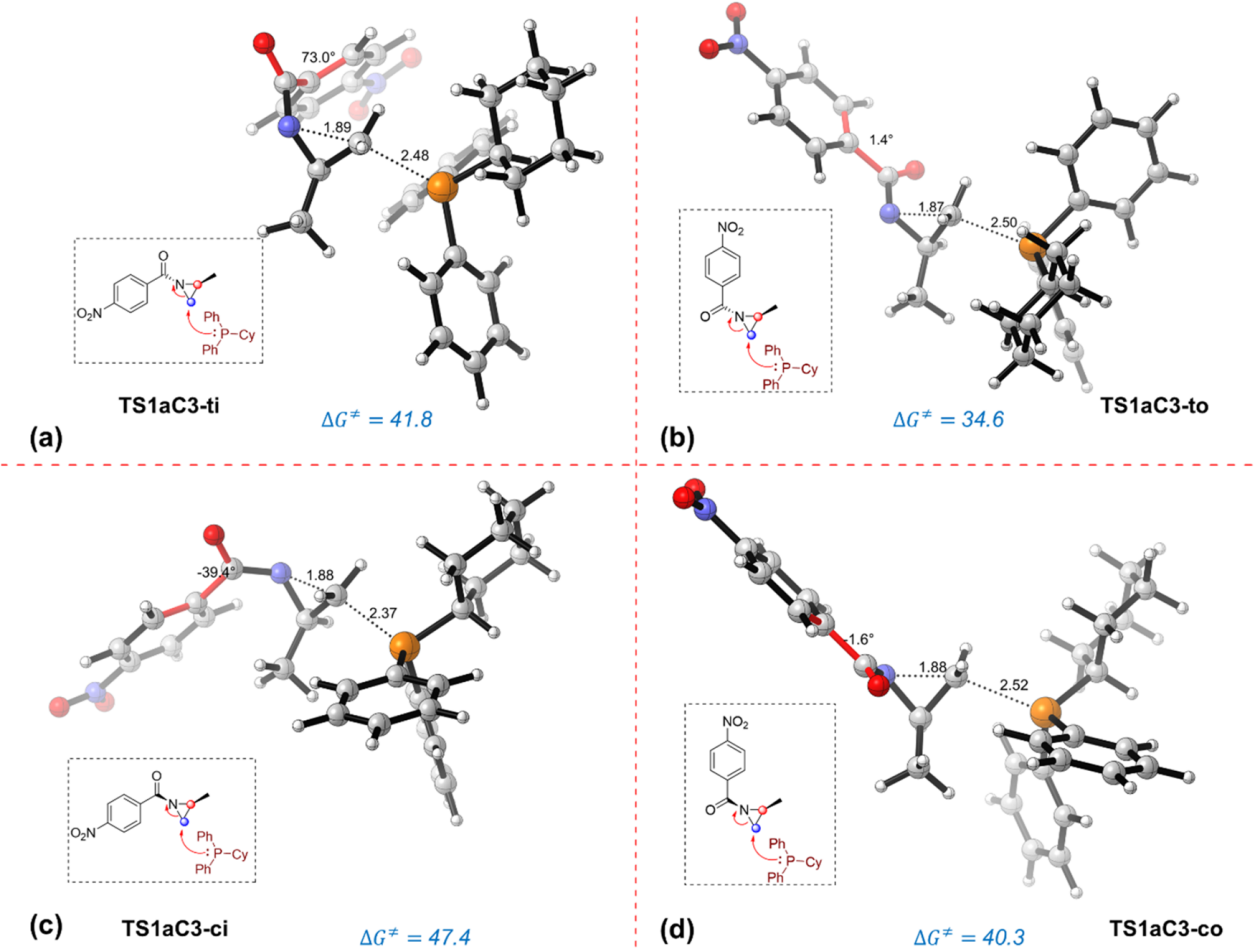
Transition state conformations (a-d) associated with the nucleophilic addition of PCyPh_2_ catalyst to the C3 carbon atom. TS structures were optimized at the SMD­(THF)-M06-2*X*/6-31+G­(d,p) level. Activation Gibbs free energies (Δ*G* ^⧧^) are reported relative to the low-lying ***trans-in*** aziridine invertomer and are given in kcal/mol; distances are given in Angstroms (Å) and dihedral angles in degrees.

**Figure 2:**
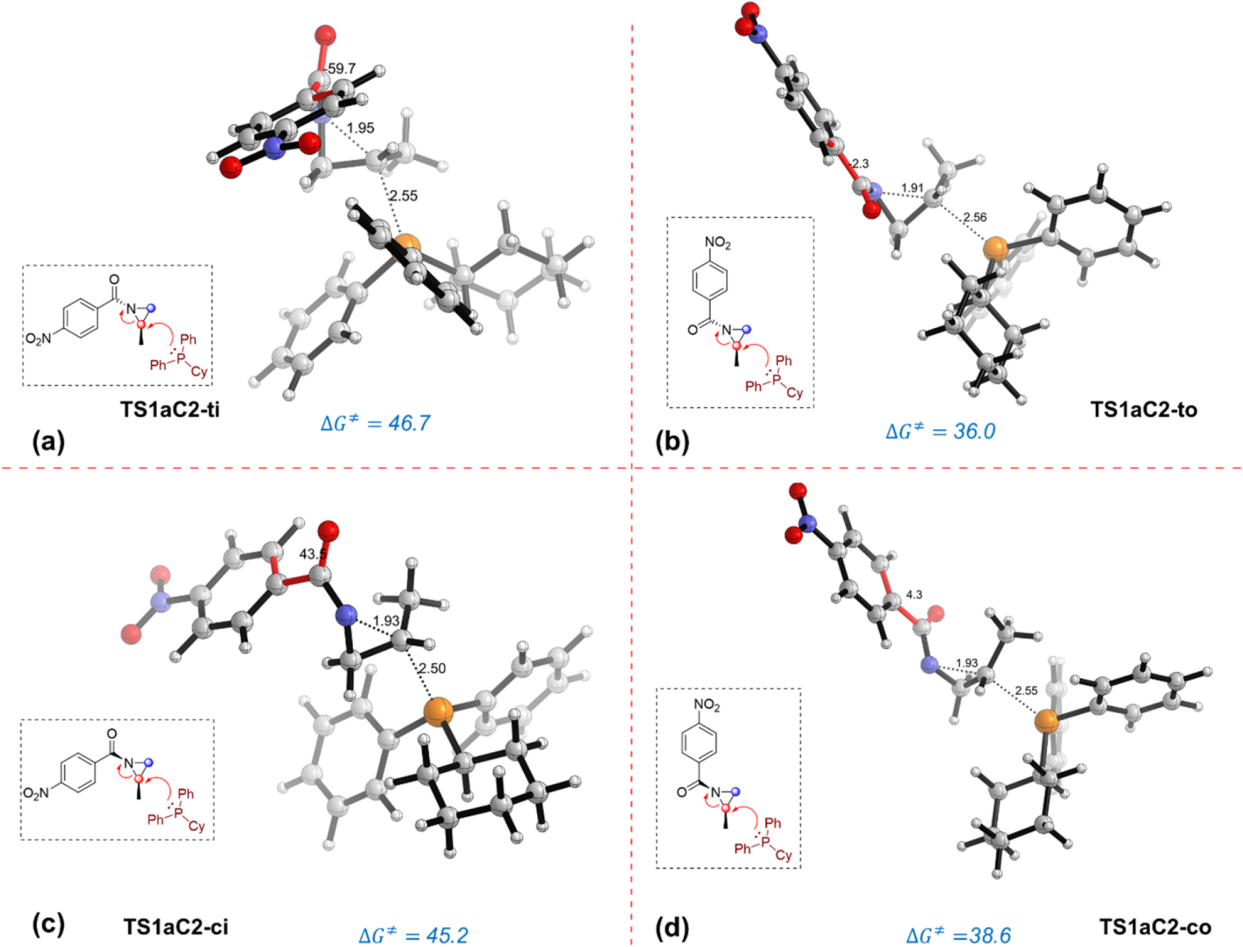
Transition state conformations (a-d) associated with the nucleophilic addition of PCyPh_2_ catalyst to the C2 carbon atom. TS structures were optimized at the SMD­(THF)-M06-2*X*/6-31+G­(d,p) level. Activation Gibbs free energies (Δ*G* ^⧧^) are reported relative to the low-lying ***trans-in*** aziridine invertomer and are given in kcal/mol; distances are given in Angstroms (Å) and dihedral angles in degrees.

Closer examination of the resulting TS structures for both competing ring-opening pathways of the model reaction (substrate **1a**) reveals that the low-lying conformations feature a nearly coplanar arrangement of the aryl and carbonyl moieties. For instance, the **TS1aC3-** ***to*** structure (Figure 1b) exhibits a molecular arrangement where the phenyl group nearly aligns with the carbonyl group, displaying a C–C–C=O dihedral angle of 1.4°. However, in the analogous **TS1aC3-** ***ti*** structure (Figure 1a), the phenyl substituent no longer lies coplanar with the carbonyl functionality, exhibiting a C–C–C=O dihedral angle of 73.0°. In the same line, the energetically favored pathway for the P–C2 bond-forming mechanism takes place through a TS structure characterized by a nearly coplanar phenyl-ketone arrangement (**TS1aC2-** ***to***) showing a dihedral angle of 2.3° (Figure 2b). This observation strongly suggests that the conformational arrangement of the benzoyl moiety dictates the reactivity patterns during the nucleophilic ring-opening stage. Natural Bond Orbital calculations indicate that the nearly coplanar arrangement in the favored transition state benefits from stronger σ­(C_aziridine_–N)→π*­(C=O) and π­(C=C,aryl)→π*­(C=O)­molecular orbital interactions (Δ*E* ^(2)^ = −54.6 and −19.5 kcal/mol, respectively) as compared to the analogous interactions in the unfavored **TS1aC3-** ***ti*** structure (ΔE^(2)^ = −52.6 and −3.5 kcal/mol, respectively).

To provide quantitative support for this hypothesis, *i.e*., the conformational arrangement of the benzoyl moiety dictates the reactivity, we conducted an Activation Strain Model (ASM) analysis on the possible C3-addition pathways. This approach involves decomposing the potential energy surface Δ*E*(ζ), along the reaction coordinate ζ, into two contributions, namely strain (Δ*E*_strain_(ζ)) that derives from the required distortion of the individual reactants from their initial equilibrium geometries plus the actual interaction Δ*E*_int_(ζ) between the increasingly deformed reactants: Δ*E*(ζ) = Δ*E*_strain_(ζ) + Δ*E*_int_(ζ).^12,19^ For the sake of clarity, we focused only on the processes involving the **TS1aC3-** ***to*** and **TS1aC3-** ***ti*** saddle points as representative cases (Figure 3).

**Figure 3:**
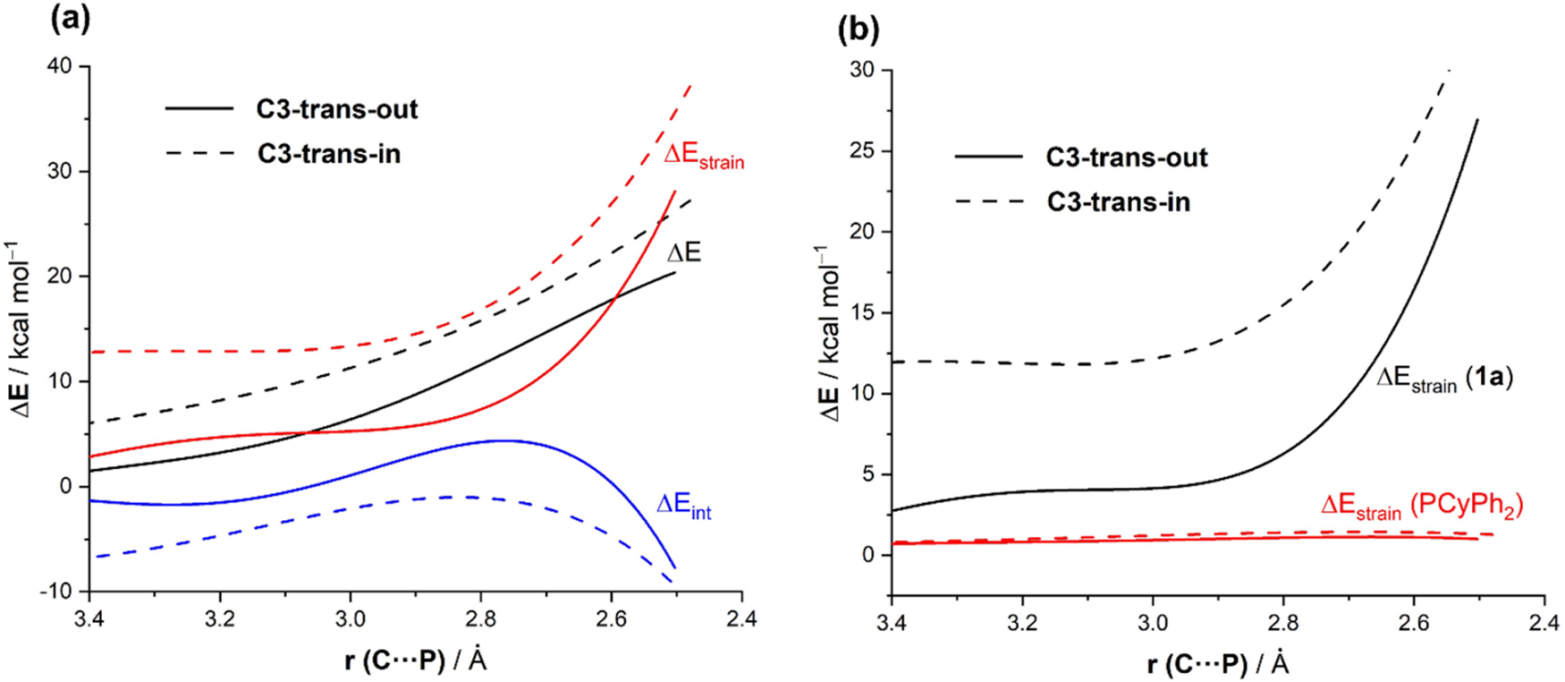
(a) Comparative activation strain diagrams for the PCyPh_2_-catalyzed ring-opening reaction of *N*-benzoylaziridine substrate **1a** illustrating the nucleophilic attack at the C3-site involving both the *trans-out* invertomer (solid lines) and *trans-in* invertomer (dotted lines), projected onto the C···P bond-forming distance. (b) Partitioning of the strain term for both processes. All data have been computed at the SMD­(THF)-M06-2*X*/6-31+G­(d,p) level.

From the comparative Activation Strain Diagrams (ASDs) depicted in Figure 3a, showing the approach of both reactants from the initial stages of the processes up to the corresponding TSs and projected onto the C···P bond-forming distance, it becomes evident that the strain term (Δ*E*_strain_) plays a key role in controlling the kinetic preference toward the *trans-out* invertomer, particularly at the TS region. Thus, although the interaction energy term (Δ*E*_int_) is more stabilizing for the *trans-in* conformation, it cannot offset the much more destabilizing strain term for this pathway as compared to the analogous reaction involving the *trans-out* conformation. For instance, at a consistent C···P bond-forming distance of 2.6 Å,^19,20^ the ΔΔ*E*_strain_ = 15.4 kcal/mol favoring the C3-*trans-out* conformation, and ΔΔ*E*_int_ = 6.3 kcal/mol favoring the C3-*trans-in* conformation, leading to an overall ΔΔ*E* = 9.1 kcal/mol favoring the pathway involving **TS1aC3-** ***to***. These results emphasize that the kinetic preference for the **TS1aC3-** ***to*** approach is determined by the less destabilizing strain energy term along the entire reaction pathway, which is directly related to the different geometrical arrangements commented above. Further partitioning of the strain term into contributions derived from each reactants confirms that indeed the deformation involving the aziridine reactant becomes almost exclusively the main source of strain, requiring the *trans-in* conformation a higher distortion to reach the corresponding transition state geometry as compared to *trans-out* (Figure 3b).

## Reaction Mechanism

Figure 4 shows the computed reaction profiles for the formation of the regioisomeric oxazoline derivatives **2a** and **3a** (red and blue pathways, respectively) involving the favored *trans-out* conformation. Our calculations predict that the nucleophilic ring-cleavage step involving the C3-attack (blue pathway) proceeds with an activation barrier of 34.6 kcal/mol (at 298 K) leading to the zwitterionic intermediate **int1a-C3** which lies 4.5 kcal/mol above the isolated reactants. Subsequently, this phosphonium intermediate can undergo an intramolecular nucleophilic *O*-attack in a *5-exo-tet* fashion^10^ via **TS2aC3** with a free energy barrier of 31.8 kcal/mol, relative to the phosphonium intermediate **int1a-C3**, affording the corresponding 4-substituted oxazoline derivative **3a** and releasing the catalyst in an exergonic reaction (Δ*G*_R_ = −10.7 kcal/mol, respect to initial reactants). The exergonicity of this second step compensates for the previous endergonic ring-opening step and drives the entire transformation forward. On the other hand, the initial nucleophilic attack at the more hindered C2 site of the aziridine ring proceeds with a barrier of 36.0 kcal/mol (via **TS1aC2-to**) leading to the endergonic formation of the phosphonium intermediate **int1a-C2** (Δ*G* = 6.5 kcal/mol relative to the separate reactants). This process is followed by an analogous intramolecular 5*-exo-tet* cyclization passing through **TS2aC2** with a free activation barrier of Δ*G* ^‡^ = 31.6 kcal/mol (relative to the zwitterionic intermediate **int1a-C2**) and leading to the exergonic formation of oxazoline derivative **2a** (Δ*G*_R_ = −11.6 kcal/mol relative to the separate reactants). It must be noted that the formation of both regioisomeric oxazoline derivatives is exergonic, with the *5*-substituted isoxazoline isomer **2a** being thermodynamically favored, which indicates that the reaction selectivity occurs entirely under kinetic control.^21^ Moreover, data in Figure 4 indicates that the selectivity of the process takes place exclusively during the initial ring-opening step. Indeed, the computed barrier energy difference for this step (ΔΔ*G* ^‡^ = 1.4 kcal/mol, involving the favored **TS1a-C3** and the regioisomeric **TS1a-C2** transition states) translates into a **3a:2a** product ratio of 92:8 (calculated from Boltzmann factor),^22^ which is in excellent agreement with the experimentally determined regioselectivity (91:9, see Scheme 2).^9^ It must be noted that a rather similar free activation barrier difference was computed at the experimental temperature (70 °C), yielding a predicted selectivity of 89:11, or using a different functional (ΔΔ*G* ^‡^ = 1.6 kcal/mol, see Figure 4), therefore providing support to the selected computational method for this study.

**Figure 4:**
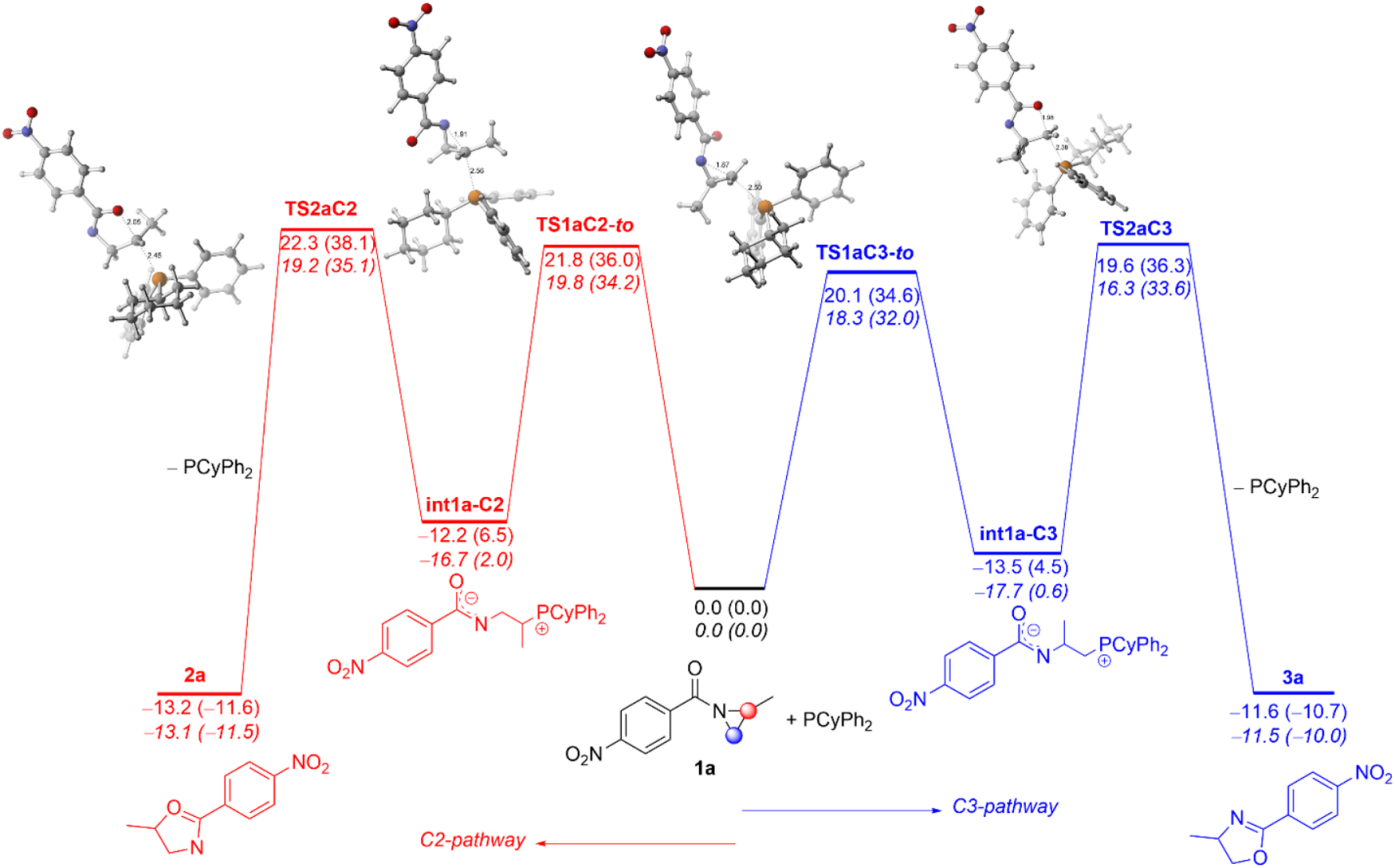
Computed reaction profile for the PCyPh_2_-catalyzed Heine reaction of *N*-benzoylaziridine **1a**. The blue profile describes the reaction pathway associated with the initial nucleophilic attack at the C3-site while the red profile represents the energetically disfavored pathway associated with the initial C2-attack. Relative total energies (free energies Δ*G*, at 298 K, within parentheses) and bond distances are given in kcal/mol and Angstroms (Å), respectively. Plain values were computed at the SMD­(THF)-M06-2*X*/6-31+G­(d,p) level whereas values in italics were computed at the SMD­(THF)-ωB97xD/6-31+G­(d,p) level. Complete numerical data are given in the SI.

To quantitatively rationalize the factors behind the observed regioselectivity in the key nucleophilic ring-cleavage step, the ASM method was applied once again. Figure 5 shows the corresponding activation strain diagrams (ASDs) computed for the C2-attack (dashed lines) and C3-attack (solid lines) for the nucleophilic ring-opening of substrate **1a**, from the beginning of the processes up to the corresponding TS region and projected onto the C···P bond-forming distance. According to these ASDs, the lower barrier computed for the C3-pathway does not derive from the interaction energy, which is actually more stabilizing for the C2-pathway, particularly at the transition state region. At variance, the strain energy becomes the main factor controlling the selectivity as it is clearly less destabilizing for the C3-pathway as compared to the C2-pathway along the entire reaction coordinate. For instance, at the same consistent C···P bond-forming distance of 2.6 Å,^19,20^ ΔΔ*E*_strain_ = 6.4 kcal/mol favoring the C3-approach, while ΔΔ*E*_int_ = 3.5 kcal/mol favoring the C2-approach, resulting in an overall ΔΔ*E* = 2.9 kcal/mol in favor of the C3-approach. The higher strain computed for the C2-approach mainly results from the considerable deformation of the reactants to avoid a significant increase in the destabilizing Pauli (steric) repulsion as the nucleophile approaches the most hindered position.

**Figure 5:**
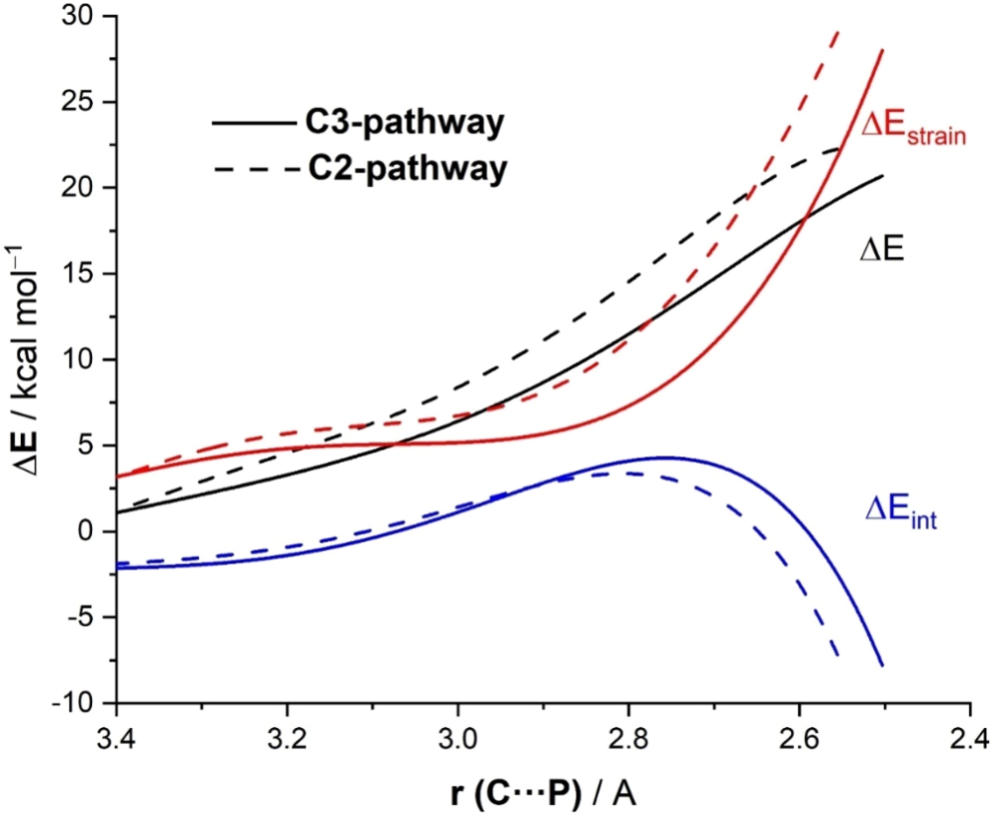
Comparative activation strain diagrams for the PCyPh_2_-catalyzed ring-opening reaction of *N*-benzoylaziridine **1a** involving a nucleophilic attack at the C3-site (solid lines) and C2-site (dotted lines), projected onto the C···P bond-forming distance. All data have been computed at the SMD­(THF)-M06-2*X*/6-31+G­(d,p) level.

## Influence of the Benzoyl Substituents

Once the mechanism and the factors controlling the selectivity of this transformation have been explored, we then focused on the influence of the substituent at the aryl ring on the reactivity. To this end, we compared the process involving **1a**, which bears an electron-withdrawing group (R = NO_2_) with the analogous reactions involving the unsubstituted aziridine **1c** (R = H) and aziridine **1d** having a donor group (R = OMe). Figure 6 shows the resulting lowest energy TS conformations for the PCyPh_2_-catalyzed ring-opening of substituted aziridines **1c** and **1d** along with the corresponding barrier heights.

**Figure 6:**
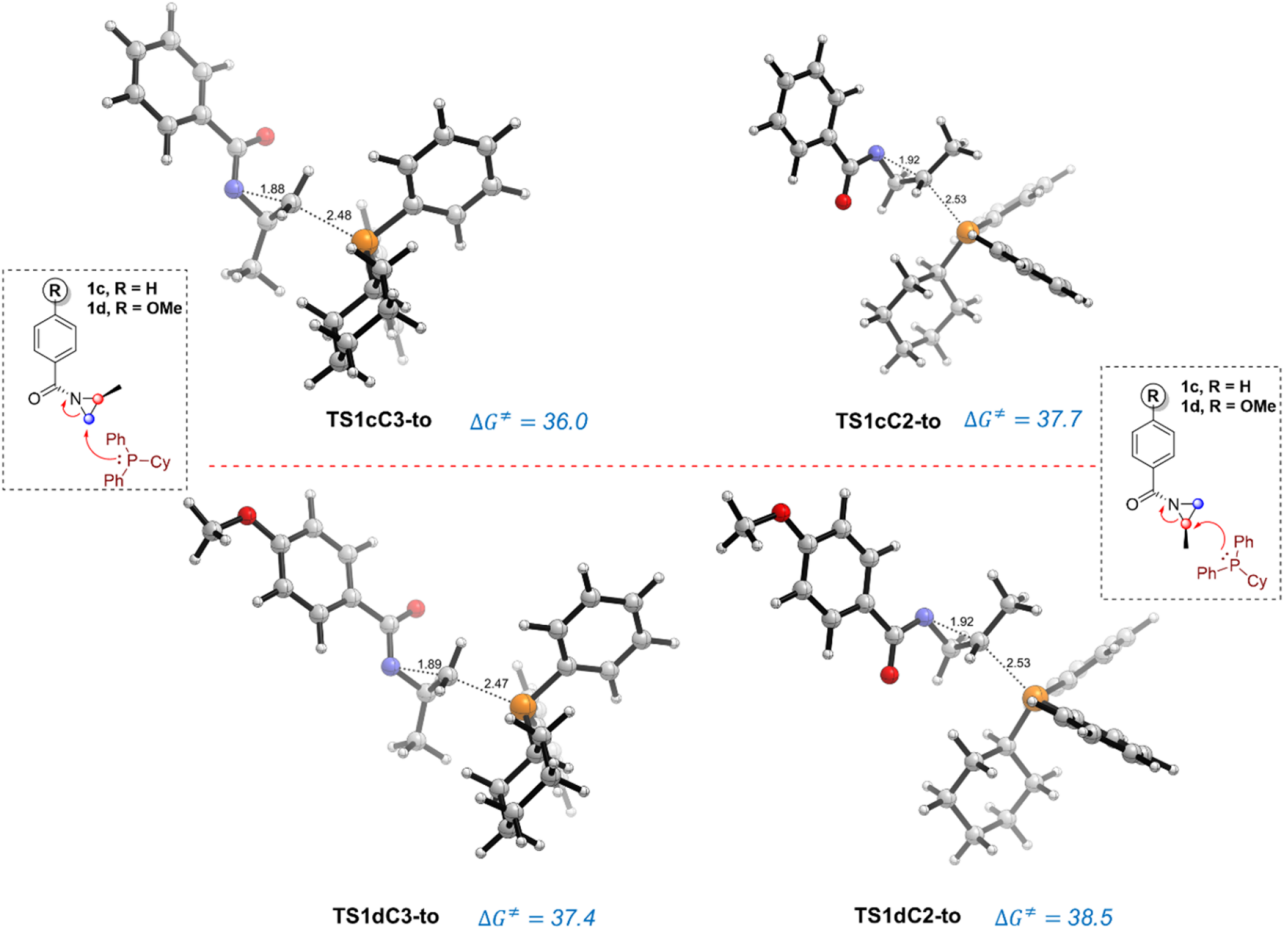
Transition state structures associated with the nucleophilic addition of phosphine catalyst to the C3 and C2 carbon atoms of aziridine derivatives **1c** (top) and **1d** (bottom). TS structures were optimized at the SMD­(THF)-M06-2*X*/6-31+G­(d,p) level. Activation Gibbs free energies (Δ*G* ^⧧^) are reported relative to the low-lying *trans* aziridine invertomer and are given in kcal/mol; whereas distances are given in Angstroms (Å) and dihedral angles in degrees.

Data in Figure 6 indicate that, regardless of the nature of the substituent, the C3-pathway is kinetically favored over the analogous ring-opening reaction involving the C2 carbon of the aziridine. Interestingly, the computed activation barriers for both competing pathways involving aziridines **1c** and **1d** are in qualitative good agreement with the experimentally observed reactivity according to the reported yields (no comparative conversion data is available). For instance, the computed free energy barrier of the C3-pathway for the parent substrate (aziridine **1a**, R = NO_2_) is 2.8 kcal/mol lower than that involving the donor methoxy group (substrate **1d**), which is consistent with the reaction yields reported for these substrates (71% for **1a** and 5% for **1d**).^9^ Similarly, for the unsubstituted benzoyl aziridine (substrate **1c**, R = H), the calculated activation barrier for the energetically favored C3-attack pathway is, not surprisingly, intermediate between the extreme situations represented by **1a** and **1d** (the associated barrier is 1.4 kcal/mol higher than that involving **1a**). Therefore, our calculations suggest that the key ring-opening reaction is facilitated by the presence of electron-withdrawing groups at the aryl group, whereas donor substituents provoke the opposite effect. Further inspection of the key bond distances for the favored C3-attack indicates that the key C3···N bond-breaking distance becomes longer and longer from **1a** (R = NO_2_) to **1c** (R = H) and to **1d** (R = OMe), which therefore follows the same trend as the reactivity trend.

Once again, we applied the ASM approach to obtain a quantitative understanding of the above-commented reactivity trend: **1a** (R = NO_2_) > **1c** (R = H) > **1d** (R = OMe). Figure 7 shows the corresponding ASDs for the corresponding ring-opening reactions involving these aziridines from the initial stages of the transformation up to the respective TS region. As depicted in Figure 7, the enhanced reactivity of aziridine **1a** does not originate from the strain energy, which is clearly less destabilizing for the processes involving **1c** and **1d**, but exclusively from a more stabilizing interaction energy between the deformed reactants, particularly in the vicinity of the TS region.

**Figure 7:**
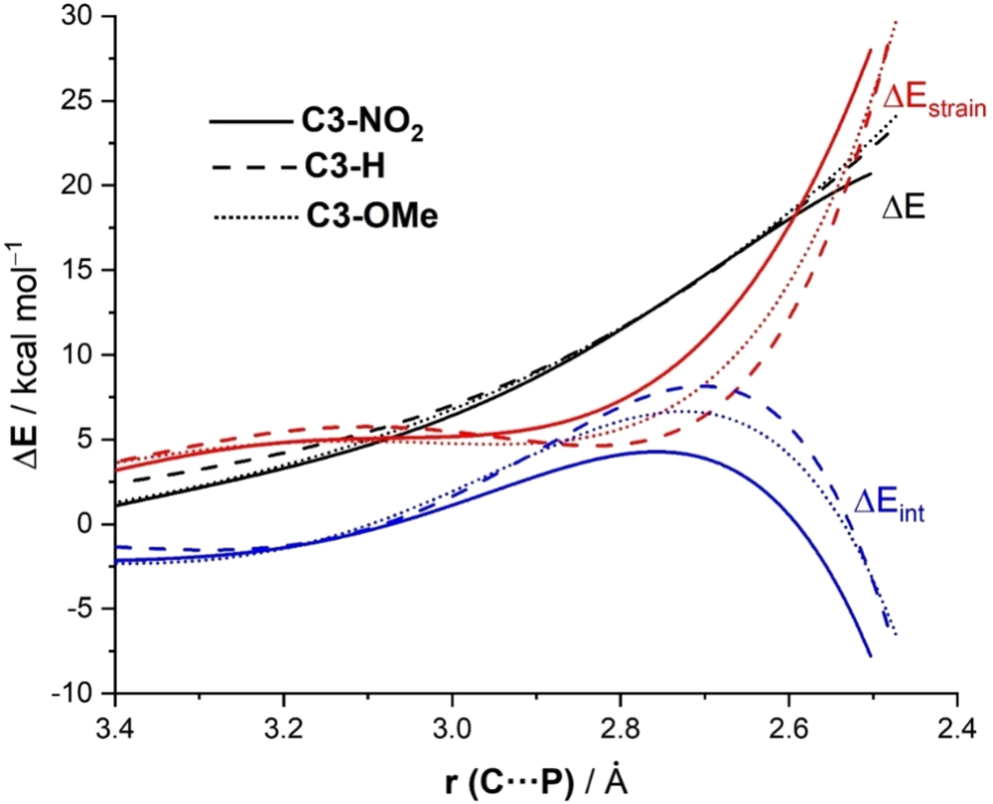
Comparative activation strain diagrams for the PCyPh_2_-catalyzed ring-opening reaction of substituted *N*-benzoylaziridines substrates **1a** (R= NO_2_, solid lines), **1c** (R= H, dotted lines) and **1d** (R= OMe, small dotted lines) involving the nucleophilic attack at the C3-site, projected onto the C···P bond-forming distance. All data have been computed at the SMD­(THF)-M06-2*X*/6-31+G­(d,p) level.

To understand the reasons behind the stronger interaction between the reactants computed for the ring-opening reaction involving **1a** in comparison with the analogous processes involving **1c** or **1d**, the EDA method was applied next. This approach involves decomposing the Δ*E*_int_ between the reactants into three chemically meaningful energy terms, namely the classical electrostatic interaction (Δ*V*_elstat_), the Pauli repulsion (Δ*E*_Pauli_) arising from the repulsion between occupied closed-shell orbitals of both deformed reactants, and the orbital interaction (Δ*E*_orb_) that accounts for charge transfer and polarization.^14^ As shown in Figure 8, which graphically shows the evolution of the EDA terms along the reaction coordinate for the extreme situations represented by the reactions involving **1a** (R = NO_2_) and **1d** (R = OMe), it becomes clear that the stronger interaction computed for the reaction involving **1a** results from both a less destabilizing Pauli repulsion and, to a higher extent, stronger electrostatic attractions between the deformed reactants. The stronger electrostatic attractions for the process involving **1a** are directly related to the electron-withdrawing nature of the NO_2_ group, which greatly facilitates the stabilization of the incipient negative charge at the transition state region, whereas the opposite is found in the electron-donor substituent. This is also reflected in the computed molecular orbital overlap (*S*) between the doubly occupied σ­(N–C3) molecular orbital in the aziridine and the lone-pair (LP) at the phosphorus atom of the phosphine, which is clearly lower in the process involving **1a** (*S* = 0.099) than in that involving **1d** (*S* = 0.116), resulting in lower (*i.e.*, less destabilizing) Pauli repulsion in the former reaction.

**Figure 8:**
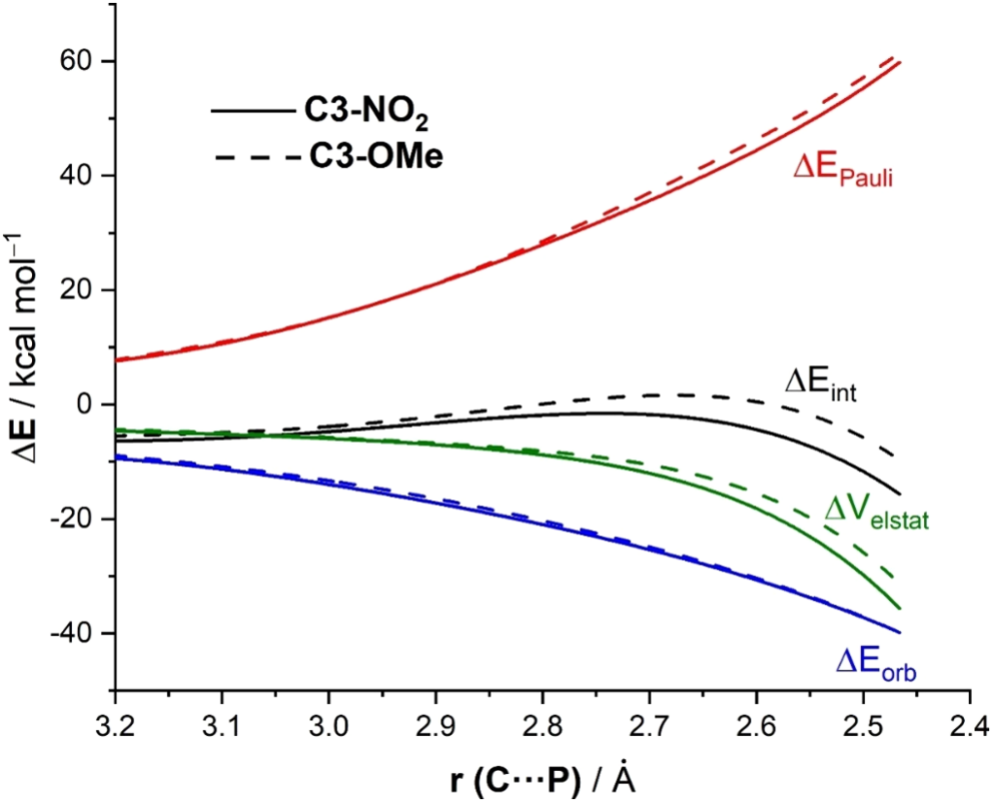
Comparative energy decomposition analyses for the PCyPh_2_-catalyzed ring-opening reaction of substituted *N*-benzoylaziridines substrates **1a** (R= NO_2_, solid lines) and **1d** (R= OMe, small dotted lines) involving the nucleophilic attack at the C3-site, projected onto the C···P bond-forming distance. All data have been computed at the ZORA-M06-2X/DZP//SMD­(THF)-M06-2*X*/6-31+G­(d,p) level.

Interestingly, the regiochemical outcome of the Heine reaction is opposite to that found in the strongly related Cloke–Wilson ring rearrangement, which occurs at the more hindered carbon atom of cyclopropanes.^23^ In this context, it is well-known that donor–acceptor cyclopropanes bearing a phenyl group as a donor fragment are prone to undergo nucleophilic ring-opening reactions involving a S_N_2 addition to the more substituted carbon atom.^24^ Encouraged by these findings, we were curious to investigate if a similar substituent effect could take place with aziridines and explored the reaction selectivity for the analogous phosphine-catalyzed Heine reaction involving *N*-benzoyl phenylaziridine **1e**. To our delight, and different from the selectivity found for its methyl-counterpart **1c** (see above), our calculations predict a reversal of regioselectivity favoring the C2-attack pathway by 0.7 kcal/mol over the expected C3-ring-opening for the PCyPh_2_-catalyzed reaction, which translates into a ratio of the corresponding oxazolines of 23:77 (see Figure 9). This predicted reversal of regioselectivity opens doors to the rational design of substituted aziridines leading to the favored (or even exclusive) formation of one or another regioisomeric oxazoline, in principle at will.

**Figure 9:**
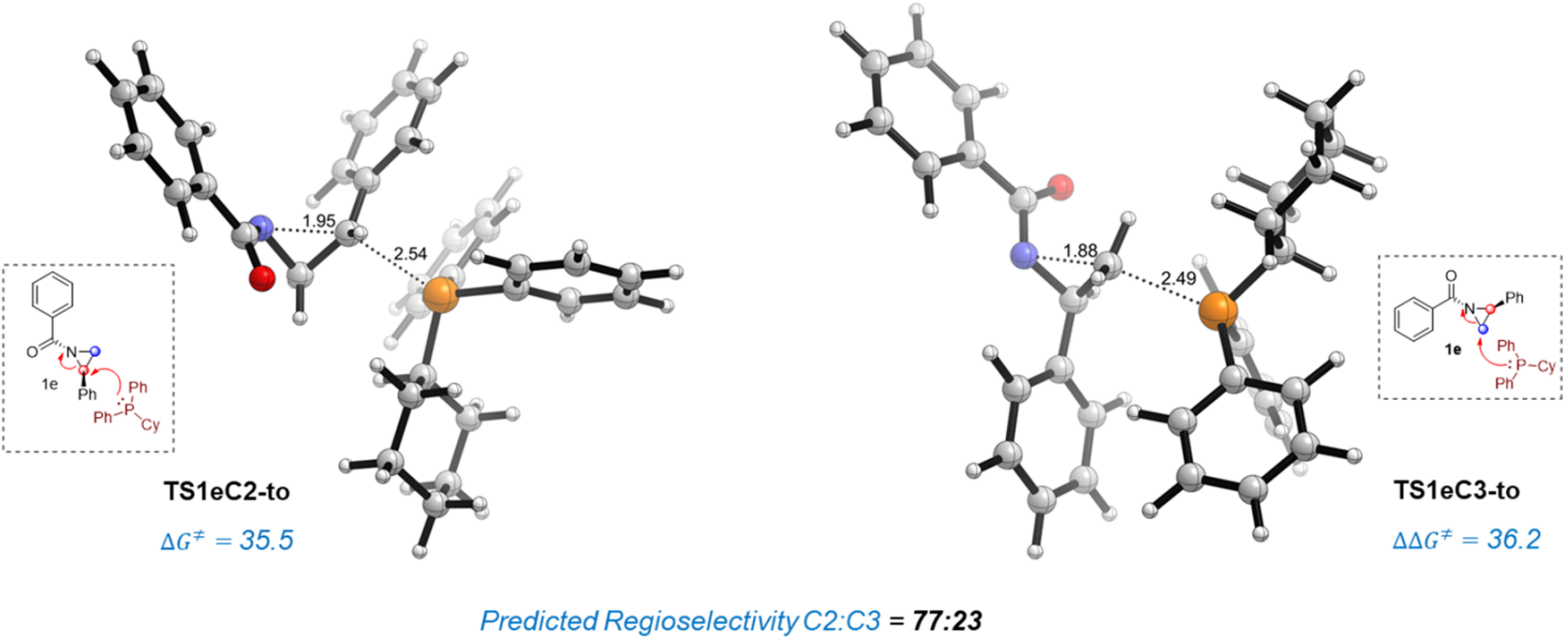
Transition state structures for the nucleophilic addition of PCyPh_2_ catalyst to the C3 and C2 carbon atoms of aziridine derivative **1e**. TS structures were optimized at the SMD­(THF)-M06-2*X*/6-31+G­(d,p) level. The relative Gibbs free energy (Δ*G* ^⧧^) value is reported in kcal/mol and key bond distances are given in Angstroms (Å). Computed selectivity was obtained from the Boltzmann factor.

We applied Conceptual-Density Functional Theory^25^ to rationalize this regioselectivity reversal. To this end, we first compared the corresponding relative electrophilicity (*s*_*k*_^+^/*s*_*k*_^–^) of C2/C3 in **1e** and in the parent system **1a**. Our calculations indicate that while the relative electrophilicity at C3 is rather similar in both species (0.47 and 0.45 in **1a** and **1e**, respectively), the C2-value in **1e** is much higher than in **1a** (0.90 vs 0.19). This simple analysis therefore suggests that the predicted regioselectivity reversal is directly related to a clear electrophilicity reversal when replacing a methyl group by a phenyl group in the initial aziridine. Further support to this explanation is provided by the Natural Orbital for Chemical Valence (NOCV)^26^ extension of the EDA method. This approach indicates that the nucleophilic attack of the phosphine to the C2 atom of **1e** is more stabilizing than the analogous addition to the C3 atom (as a consequence of its higher electrophilicity). As shown in Figure 10, the corresponding stabilization energies, ΔE­(ρ), computed at the same consistent C···P bond-forming distance of 2.55 Å, confirm that the key LP­(P)→σ*­(C–N) molecular orbital interaction is stronger when involving the C2 atom than C3 (Δ*E*(ρ) = −20.9 vs −18.1 kcal/mol).

**Figure 10:**
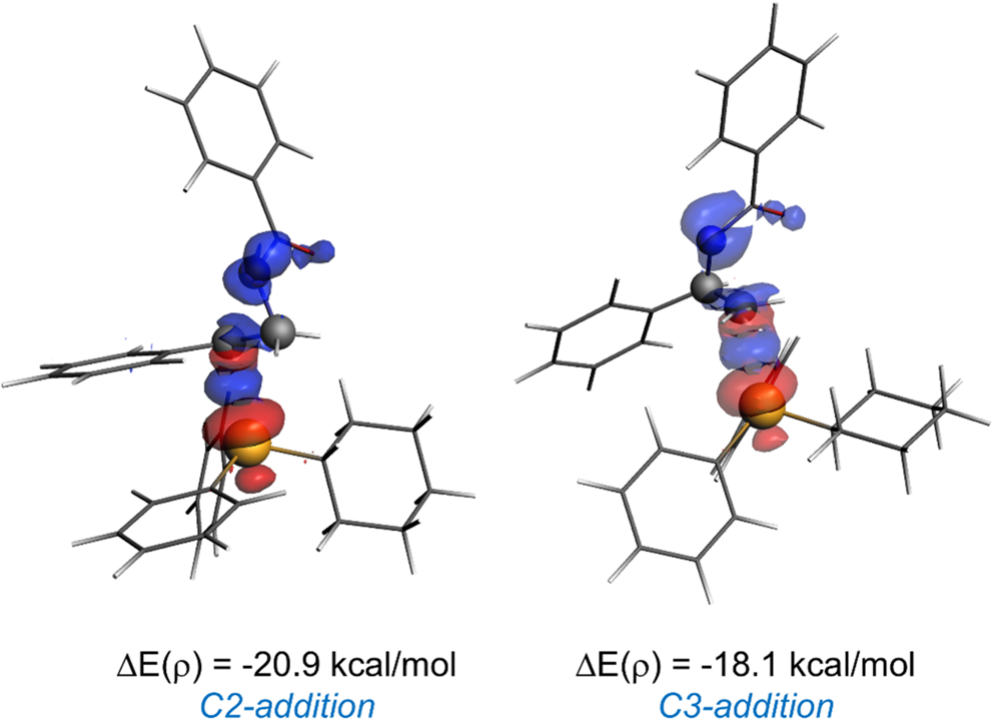
Contour plots of the main NOCV deformation densities ρ (isosurface value of 0.0015 au) and associated energies Δ*E*(ρ) for the possible nucleophilic additions to **1e**. The electronic charge flows from red to blue. All data have been computed at the ZORA-M06-2X/DZP//SMD­(THF)-M06-2*X*/6-31+G­(d,p) level.

## Conclusions

In this work, we have presented a detailed mechanistic investigation of the phosphine-catalyzed Heine reaction. DFT calculations revealed that the regioselectivity of the process takes place under kinetic control, favoring the formation of 4-substituted oxazoline derivatives as the major products. The preference for the *trans-out* arrangement of the aziridine invertomer is entirely determined by the strain energy, with the nearly coplanar arrangement of the benzoyl moiety facilitating the nucleophilic ring-opening process. Furthermore, our quantitative analysis using the ASM of reactivity in conjunction with the EDA framework has provided a deeper understanding of the factors influencing the regioselectivity and the role of the substituents on the reactivity. We have observed that aziridine distortion significantly governs the regiochemical outcome, while the reactivity is enhanced by electron-withdrawing groups at the aryl group attached to the aziridine nitrogen atom (which reduce the Pauli repulsion and increase the electrostatic interactions between the deformed reactants). Importantly, our calculations predict the possibility of reversing the regioselectivity by simply attaching a phenyl substituent at the C2-position of the aziridine. Therefore, our findings not only provide a rationale for the so far poorly understood reactivity and selectivity patterns in this organocatalytic transformation but also offer valuable mechanistic insights for the design of new regioselective organocatalyzed transformations.

## Computational Methods

All DFT calculations were performed with the Gaussian 16 suite of programs.^27^ Unless stated otherwise, all stationary points discussed in this work were optimized using the M06-2X functional^17^ together with the 6-31+G­(d,p) basis set.^28^ This level of theory is well-suited for computing activation barriers and has been proven to provide accurate results for a wide range of organic transformations.^29^ Solvation corrections were incorporated with the SMD model,^30^ which was applied to both optimizations and frequency calculations to mimic solvent effects by tetrahydrofuran (THF) used in the experimental study.^9^ Intrinsic Reaction Coordinate (IRC) calculations were performed to confirm the nature of the proposed TS structures.^31^ Thermal contributions to free energies were calculated from vibrational frequencies using the quasi-rigid rotor-harmonic oscillator (QRRHO) approach proposed by Grimme^32^ and implemented in the GoodVibes code without scaling factors.^33^ The EDA calculations were carried out with the ADF 2022.103 program package^34^ using the SMD­(THF)-M06-2*X*/6-31+G­(d,p) optimized geometries at the same M06-2X level in conjunction with a double-ζ-quality basis set using uncontracted Slater-type orbitals augmented by one set of polarization functions.^35^ Auxiliary sets of s, p, d, f, and g STOs were used to fit the molecular densities and to represent the Coulomb and exchange potentials accurately in each SCF cycle.^36^ Scalar relativistic effects were incorporated by applying the zeroth-order regular approximation (ZORA).^37^ This level of theory is denoted ZORA-M06-2X/D2P//SMD­(solvent)-M06-2*X*/6-31+G­(d,p). Molecular visualization of different competing TS structures was carried out with CYLview.^38^

## Supplementary Material



## Data Availability

The data underlying this study are available in the published article and its Supporting Information.

## Notes

3D DFT-optimized geometries of the four invertomers of aziridines **1a**–**d** (Figures S1–S4) and the TS structures for the PMe_3_-catalyzed ring-opening mechanism (Figures S5 and S6), Cartesian coordinates (in Å) and energies (in Hartrees) for all structures discussed in the text (PDF)
