# Supplementary material for: Mechanism and Origin of Regioselectivity in the Phosphine-Catalyzed Heine Reaction
Source: J Org Chem. 2025 May 7;90(19):6538–48. doi: 10.1021/acs.joc.5c00416 (PMC12123667; doi:10.1021/acs.joc.5c00416)
Supplement: Supplementary file 1 [file jo5c00416_si_001.pdf]

Supporting Information for

Mechanism and Origin of Regioselectivity in the Phosphine-Catalyzed Heine Reaction

Sebastián Gallardo-Fuentes[†]*, Lucas Lodeiro[‡] and Israel Fernández[≠]*

Table of contents

Figures S1-S4	S2-S3
Figures S5-S6	S4-S5
Cartesian coordinates, energies, and NIMAG of all structures optimized at the SMD(THF)-M06-2X/6-31+G(d,p) level of theory.	S7-S54
Summary of the energies and NIMAG of all structures optimized at the SMD(THF)-M06-2X/6-31+G(d,p) level of theory.	S57-S58
Cartesian coordinates, energies, and NIMAG of all structures optimized at the SMD(THF)-ωB97X-D/6-31+G(d,p) level of theory.	S59-S68

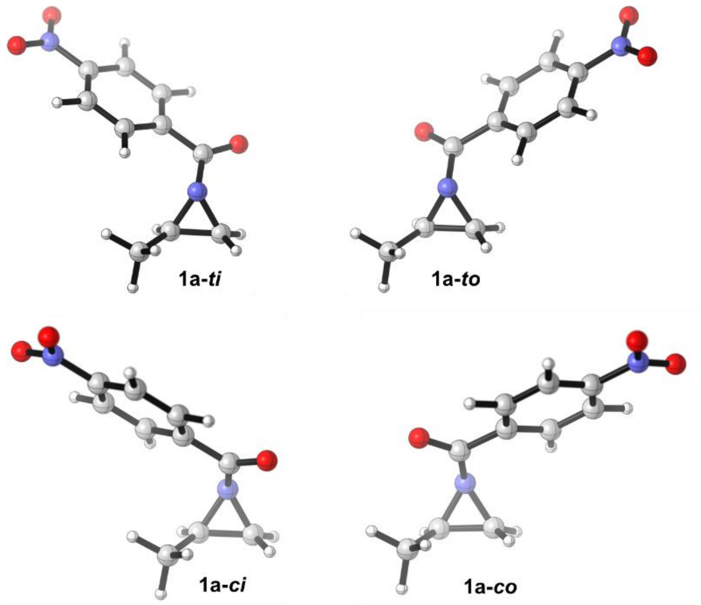

Figure S1: DFT-optimized geometries of the four invertomers (**1a-ti**, **1a-to**, **1a-ci**, and **1a-co**) of aziridine **1a**.

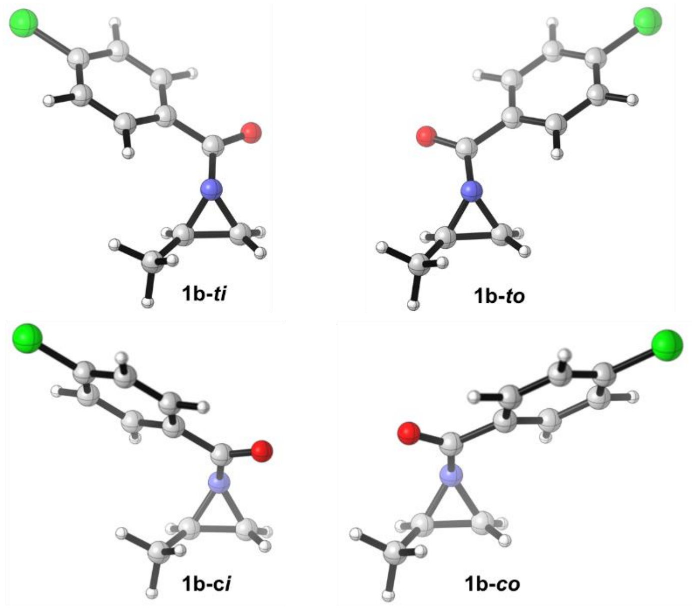

Figure S2: DFT-optimized geometries of the four invertomers (**1b-ti**, **1b-to**, **1b-ci**, and **1b-co**) of aziridine **1b**.

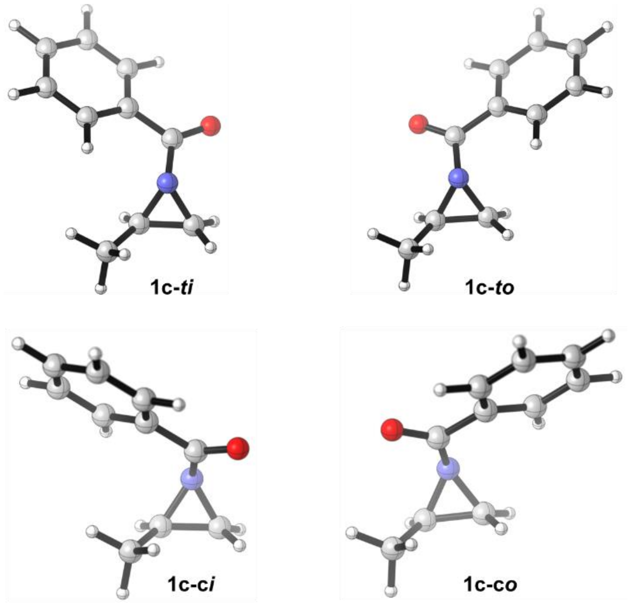

Figure S3: DFT-optimized geometries of the four invertomers (**1c-ti**, **1c-to**, **1c-ci**, and **1c-co**) of aziridine **1c**.

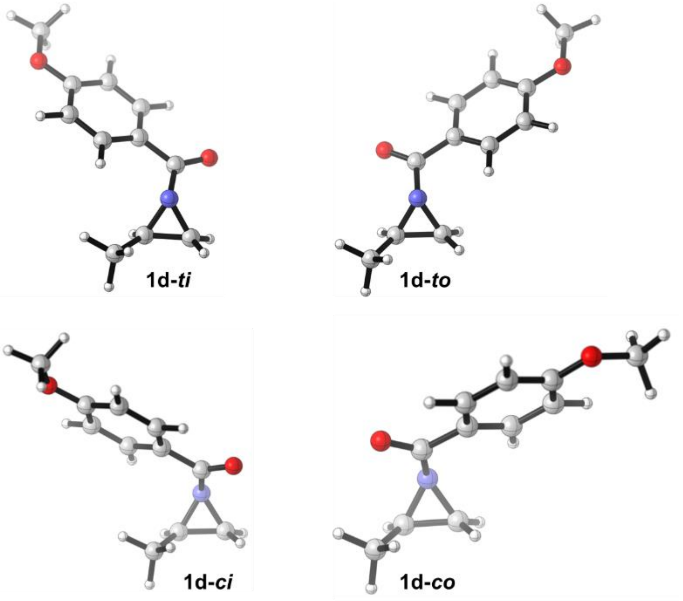

Figure S4: DFT-optimized geometries of the four invertomers (**1d-ti**, **1d-to**, **1d-ci**, and **1d-co**) of aziridine **1d**.

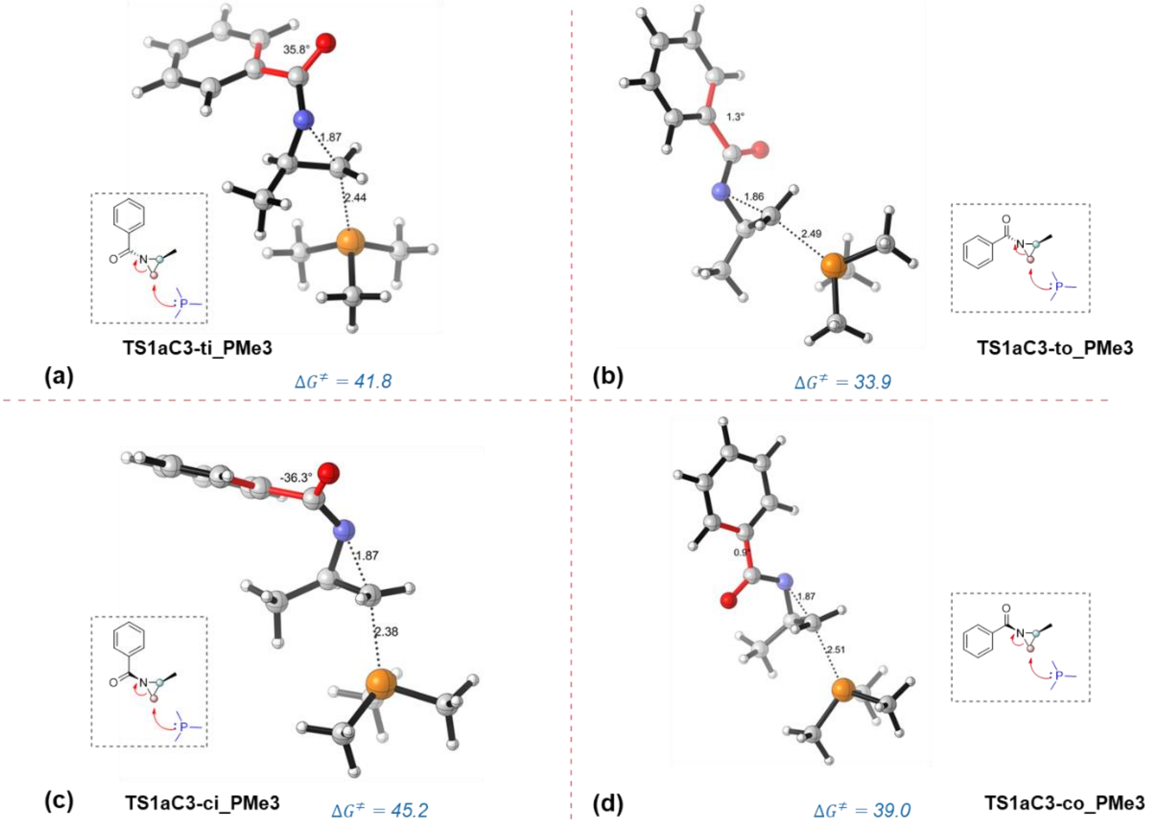

Figure S5. Transition state conformations associated with the nucleophilic addition of PMe_3 catalyst to the C3 carbon atom. TS structures were optimized at the SMD(THF)-M06-2X/6-31+G(d,p) level. Activation Gibbs free energies ($\Delta G^{\ddagger}$) are reported relative to the low-lying ***trans-in*** aziridine invertomer and are given in kcal/mol; distances are given in Angstroms (Å) and dihedral angles in degrees.

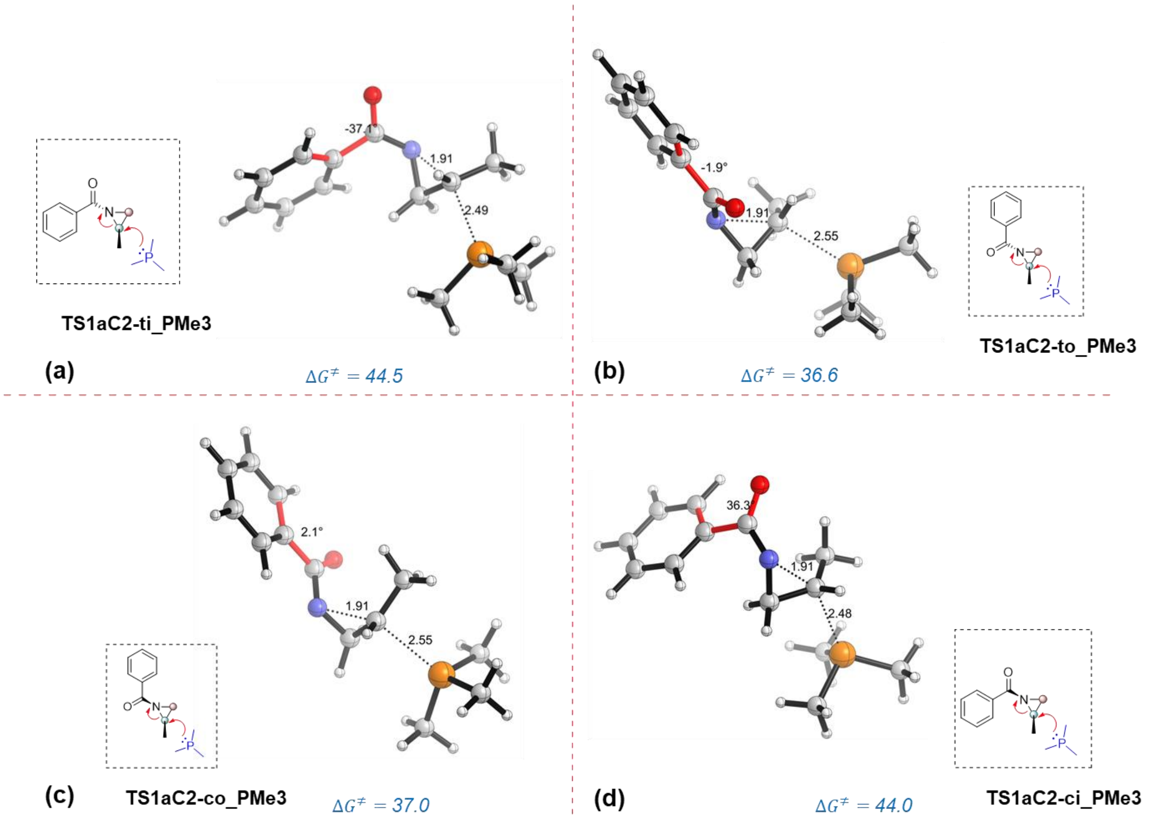

Figure S6. Transition state conformations associated with the nucleophilic addition of PMe3 catalyst to the C2 carbon atom. TS structures were optimized at the SMD(THF)-M06-2X/6-31+G(d,p) level. Activation Gibbs free energies ($\Delta G^{\ddagger}$) are reported relative to the low-lying ***trans-in*** aziridine invertomer and are given in kcal/mol; distances are given in Angstroms (Å) and dihedral angles in degrees.

Cartesian coordinates, energies, and NIMAG of all structures optimized at the SMD(THF)-M062X-D/6-31+G(d,p) level of theory.

1a-ti
E(RM062X) = -721.896203874
Sum of electronic and thermal Free Energies = -721.744131
Charge = 0 Multiplicity = 1 NIMAG = 0

C	0.2527337685	2.1810609987	-0.1899832626
C	1.3790023992	1.2068545042	-0.0309233348
H	1.8993872671	0.8648190672	-0.9216337811
H	1.3528356363	0.5130869755	0.8048100154
N	1.5536570654	2.5958183865	0.3261264252
C	2.4308449463	3.4130275363	-0.3653767896
O	3.4989585201	3.0226011794	-0.8035164732
C	2.0078074333	4.8504232982	-0.478975805
C	2.6614197912	5.6573282252	-1.4146733538
C	0.9954439243	5.3805913654	0.3269280479
C	2.3023700656	6.9915466304	-1.5608354751
H	3.4492898066	5.2317368714	-2.0268231017
C	0.6289428144	6.715768847	0.1982286278
H	0.5060478451	4.7581397616	1.0683356986
C	1.290215612	7.489970046	-0.7478080809
H	2.7917768372	7.631616141	-2.284968089
H	-0.1470734456	7.1482477231	0.8180118973
N	0.9037782311	8.9034881617	-0.8928446173
O	0.0052332287	9.325893434	-0.184193813
O	1.5019276193	9.5774459605	-1.7147808122
C	-0.9108183571	2.2066009812	0.7618700692
H	-1.3559668388	3.2050953787	0.8118430377
H	-1.6831449321	1.5074688872	0.4277418584
H	-0.5859241105	1.9165608868	1.7652148749
H	0.0478859024	2.4930791131	-1.2133961229

1a-to
E(RM062X) = -721.895485602
Sum of electronic and thermal Free Energies = -721.743408
Charge = 0 Multiplicity = 1 NIMAG = 0

C	0.3733302645	1.6166889718	0.8405184291
C	1.5512880343	1.3490920918	-0.0463865196
H	-0.3838847389	0.8349260583	0.8719251685
H	1.5886610305	0.3988268214	-0.5733303283
H	2.5092716908	1.8042010816	0.1909229073
C	0.5008317235	2.4727532411	2.071846092
H	-0.4434357922	2.9824520553	2.2842750351
H	0.7620898469	1.8564776561	2.9368843905
H	1.2800934435	3.2272436531	1.9309037361
N	0.5011081194	2.2784301233	-0.4378527495
C	-0.3798477492	2.0576634923	-1.4808233129
O	-1.5795836519	1.9212612588	-1.3140392844
C	0.2265951985	2.0789211587	-2.8564758802
C	-0.5453891677	1.602642789	-3.9198875188
C	1.5180338923	2.5653119337	-3.0836681757
C	-0.0336298261	1.59978122	-5.2115518203
H	-1.5472220036	1.2347906981	-3.7262720608
C	2.0425604882	2.5758175366	-4.3713053951

H	2.1110211093	2.9512189062	-2.261560401
C	1.253201867	2.0887639245	-5.4064539658
H	-0.6142242224	1.2297601858	-6.0478148144
H	3.0375494961	2.9548411047	-4.5706506394
N	1.804260952	2.0942497458	-6.7718743316
O	2.9405409707	2.5096899942	-6.9275902856
O	1.0956851344	1.6826211279	-7.6752311651

1a-ci
E(RM062X) = -721.895848840
Sum of electronic and thermal Free Energies = -721.743209
Charge = 0 Multiplicity = 1 NIMAG = 0

C	-3.8720702439	0.1848148189	0.4269799022
C	-2.815083739	1.1166069316	0.9369251948
N	-2.5738142966	0.2345093478	-0.2000033906
C	-1.7119716295	-0.8429553163	-0.2210522407
O	-2.0921321866	-1.9954778682	-0.3472653183
C	-0.2501715612	-0.4962228639	-0.1671060436
C	0.6602721895	-1.5283536581	0.076372542
C	0.2014175003	0.8141840469	-0.3543053341
C	2.022043722	-1.2604724732	0.1432168465
H	0.2927713176	-2.5395414253	0.2140099893
C	1.5612067962	1.0983138938	-0.296819255
H	-0.5037506461	1.6125324898	-0.5602494646
C	2.4408760661	0.0517632639	-0.0459258082
H	2.7432283109	-2.0452950667	0.3364484787
H	1.9330153034	2.1046849223	-0.4464035477
N	3.8822103902	0.3459459262	0.0173869549
O	4.2361066152	1.5034989162	-0.1338724092
O	4.6465000591	-0.5832564585	0.2176755032
H	-4.6954032386	0.5721780684	-0.1649309512
H	-4.0856579057	-0.7000389473	1.0231180936
H	-2.8955835414	2.1641816327	0.6549951617
C	-2.1377969458	0.8420809668	2.2544602029
H	-1.1208084994	1.2458567176	2.2708485042
H	-2.7034637839	1.3155716513	3.0621365303
H	-2.0886768131	-0.2337060466	2.4543428388

1a-co
E(RM062X) = -721.894030899
Sum of electronic and thermal Free Energies = -721.742087
Charge = 0 Multiplicity = 1 NIMAG = 0

C	3.0311657682	-0.8502997344	-0.5244607153
C	4.0548126612	0.1945326453	-0.1849349951
H	4.8744433062	-0.1126692259	0.4607648733
N	2.7658599776	0.1667139325	0.4726139952
C	1.8181581869	1.162561433	0.4290185417
O	2.0925450191	2.3416599012	0.5865477188
C	0.3928139766	0.7045587262	0.2743843298
C	-0.5572229906	1.6523397946	-0.1158481494
C	0.0091528371	-0.6167188499	0.5258726293
C	-1.888587288	1.2870516721	-0.2737409279
H	-0.2440891047	2.6751266495	-0.2957725915
C	-1.3211009364	-0.9958198343	0.3851930739
H	0.7412783727	-1.3492021492	0.8487863755
C	-2.2394786806	-0.0337069543	-0.0185658923

H	-2.6382950107	2.004739893	-0.5839905953
H	-1.6410667309	-2.0112965558	0.5846275161
N	-3.6476007282	-0.4308690285	-0.1811477948
O	-3.944723257	-1.5909534246	0.0515155369
O	-4.4432150456	0.4206322621	-0.5413893937
H	3.1363630198	-1.8624145947	-0.1448963468
H	2.5236087063	-0.747393576	-1.4816386824
C	4.3718231208	1.282608751	-1.177882566
H	4.6642891939	2.2061872067	-0.6725295938
H	5.1997699928	0.9581885803	-1.8150913477
H	3.5085208236	1.4973563202	-1.8144731684

TS (1a-ti to 1a-ci)
E(RM062X) = -721.8877191
Sum of electronic and thermal Free Energies = -721.735256
Charge = 0 Multiplicity = 1

C	3.172280000	1.205059000	0.200752000
C	4.165627000	0.056925000	0.228905000
H	4.643443000	-0.195011000	1.173401000
H	4.747253000	-0.134830000	-0.671077000
N	2.766485000	-0.159251000	0.137390000
C	1.843521000	-1.130144000	0.165647000
O	2.134021000	-2.324310000	0.177440000
C	3.008573000	2.058049000	-1.030357000
H	1.984933000	2.438082000	-1.105025000
H	3.689163000	2.913135000	-0.986751000
H	3.229825000	1.474452000	-1.928429000
H	2.995838000	1.699498000	1.156465000
C	0.405735000	-0.679899000	0.131647000
C	0.003216000	0.590517000	0.554547000
C	-0.542555000	-1.598316000	-0.327972000
C	-1.339519000	0.950244000	0.511882000
C	-1.886342000	-1.251547000	-0.389633000
C	-2.254988000	0.020585000	0.033643000
H	0.725336000	1.302252000	0.937833000
H	-0.215903000	-2.584865000	-0.638299000
H	-1.670759000	1.926849000	0.843966000
H	-2.631661000	-1.948303000	-0.754197000
N	-3.676136000	0.397524000	-0.022599000
O	-4.467188000	-0.420874000	-0.461351000
O	-3.989735000	1.509181000	0.370687000

TS (1a-ti to 1a-to)
E(RM062X) = -721.8883399
Sum of electronic and thermal Free Energies = -721.735006
Charge = 0 Multiplicity = 1

C	-3.911728000	-0.208856000	0.301460000
C	-3.731676000	0.023343000	-1.137154000
H	-3.774532000	1.047845000	-1.495673000
H	-3.993172000	-0.755923000	-1.846900000
N	-2.556421000	-0.319733000	-0.293558000
C	-1.702827000	0.785193000	-0.034331000
O	-2.093640000	1.917767000	0.162972000
C	-0.241179000	0.449469000	-0.020715000
C	0.674935000	1.490020000	0.171671000
C	0.206344000	-0.862883000	-0.197442000

C	2.038043000	1.228237000	0.186865000
H	0.309846000	2.502425000	0.307418000
C	1.569400000	-1.142334000	-0.180395000
H	-0.512565000	-1.659717000	-0.344881000
C	2.452952000	-0.087943000	0.009496000
H	2.764051000	2.018799000	0.332686000
H	1.939082000	-2.151938000	-0.313240000
N	3.897951000	-0.375572000	0.025445000
O	4.250729000	-1.537951000	-0.082963000
O	4.664934000	0.564965000	0.146264000
C	-4.480132000	-1.494281000	0.835749000
H	-4.056859000	-1.728137000	1.817025000
H	-5.566077000	-1.408957000	0.941876000
H	-4.260585000	-2.321023000	0.154337000
H	-4.060885000	0.685719000	0.902721000

3a
E(RM062X) = -721.914709587
Sum of electronic and thermal Free Energies = -721.762349
Charge = 0 Multiplicity = 1 NIMAG = 0

C	-0.6552111631	3.2695033434	-0.0771806187
C	0.3916729314	3.2444021898	0.8489454532
C	1.3530372732	4.2493719383	0.8404481043
C	1.2390935183	5.2610079355	-0.1044216974
C	0.2078905447	5.3105292344	-1.0380806765
C	-0.744301493	4.3014626272	-1.018741582
H	0.4554422084	2.4421870727	1.5751694077
H	2.1720092636	4.2488859238	1.5494220086
H	0.1565459454	6.1182041898	-1.7580283445
H	-1.5618343244	4.3063030169	-1.731387886
C	-1.6794085608	2.2042011791	-0.07362751
N	2.2523320092	6.3273100173	-0.1181577938
O	2.1426259599	7.2093059365	-0.9539728574
O	3.1496559368	6.2745768273	0.7068920591
O	-1.5114257527	1.2258841968	0.8472614749
N	-2.6790021896	2.1346713646	-0.857111554
C	-3.4126505139	0.9043177636	-0.5118304935
C	-2.6655991916	0.3629963898	0.7278583416
H	-2.3129401957	-0.663424295	0.6202175491
H	-3.2535182035	0.4591420269	1.6461399348
C	-4.8838386748	1.1921915064	-0.2538772738
H	-5.3570759828	1.6052337076	-1.1486955225
H	-5.4083671704	0.2723155218	0.0232824357
H	-4.9898677967	1.913644827	0.5629796239
H	-3.3208010379	0.2130260586	-1.3573315128

2a
E(RM062X) = -721.917187424
Sum of electronic and thermal Free Energies = -721.762588
Charge = 0 Multiplicity = 1 NIMAG = 0

C	-0.3970171298	3.2260001746	-0.1764473936
C	-0.4170109821	2.935939469	1.1908968094
C	0.6401854539	3.3327287679	2.0029385764
C	1.6990139962	4.0147198866	1.4173092868
C	1.7447867368	4.3168658756	0.0594162887
C	0.682935266	3.9166286444	-0.7388906851

H	-1.2567395121	2.4011897296	1.6197762381
H	0.6440609046	3.1179912303	3.0646848717
H	2.5904587202	4.8516736825	-0.3554452471
H	0.6830015203	4.1359732422	-1.8009995584
C	-1.5180395931	2.8077816785	-1.0436479866
N	2.8219658383	4.4358908477	2.2688141658
O	3.7506540005	5.024613026	1.7400324281
O	2.7668078616	4.1745612759	3.4593276681
O	-2.5430814503	2.1744301169	-0.4291968623
N	-1.5920325884	3.0025711157	-2.2995760884
C	-2.89108148	2.4670657152	-2.7246888471
C	-3.4337956151	1.7193933671	-1.4878975508
H	-4.4400064293	2.0379885804	-1.2089436976
H	-2.7634034764	1.7983694194	-3.5813952849
H	-3.5356363531	3.2960417664	-3.0343644737
C	-3.3411215748	0.211512411	-1.5933189022
H	-3.6136550394	-0.263927214	-0.6474306948
H	-4.0231945557	-0.1441241296	-2.3717441652
H	-2.3220745888	-0.0875796795	-1.8615243454

1b-ti
E(RM062X) = -977.030667360
Sum of electronic and thermal Free Energies = -976.888653
Charge = 0 Multiplicity = 1 NIMAG = 0

C	3.0798941847	-0.8813412121	-0.657208041
C	4.0665587542	0.130427712	-0.1643264902
H	4.2699802535	1.0004400446	-0.7830070983
H	4.8790276983	-0.201944738	0.4759621477
N	2.7469271172	0.0466580914	0.4191054465
C	1.8364366148	1.0852342679	0.2732345846
O	2.1733263962	2.2574660729	0.2629713484
C	0.4001620428	0.6698795945	0.1984465513
C	-0.5436116148	1.6157649567	-0.2112182435
C	-0.0126335838	-0.6239658356	0.530345824
C	-1.888971775	1.2764270511	-0.3038985444
H	-0.2156058724	2.6198944397	-0.4603419778
C	-1.3569995887	-0.9757046927	0.4489784332
H	0.7110494677	-1.3566000299	0.8730773165
C	-2.276712786	-0.019484942	0.0279399615
H	-2.6251980469	2.0044991471	-0.6273943565
H	-1.6856558578	-1.9751274619	0.7132104875
C	3.1982813093	-2.3386721102	-0.3064160928
H	2.2193932394	-2.8278321839	-0.3232134389
H	3.8434927519	-2.8470732039	-1.0291847737
H	3.6311005084	-2.4539583362	0.6915092861
H	2.6175650585	-0.6518847552	-1.6167196928
Cl	-3.9653861818	-0.4561070965	-0.0836975374

1b-to
E(RM062X) = -977.029964349
Sum of electronic and thermal Free Energies = -976.888358
Charge = 0 Multiplicity = 1 NIMAG = 0

C	-3.8843349282	0.2217619509	0.4341255977
C	-2.8044765481	1.1519606526	0.8954172369
H	-4.0504549243	-0.6562402017	1.0563629645
H	-2.2822113438	0.9100745461	1.8179147495

H	-2.8669646545	2.2075075836	0.643585173
C	-5.0730824243	0.7246674525	-0.3401555269
H	-5.4484420382	-0.0509976673	-1.014100226
H	-5.8795562181	1.0076504583	0.342567828
H	-4.7958979432	1.5989720559	-0.9361615743
N	-2.602050993	0.2533396458	-0.2316970111
C	-1.7313270256	-0.8264483566	-0.2070752288
O	-2.1210959636	-1.979576462	-0.2894846502
C	-0.2743292204	-0.483559137	-0.1515844853
C	0.6367783126	-1.5112738119	0.1067953127
C	0.1897158414	0.8196953949	-0.3548033319
C	2.0003145792	-1.2467494776	0.1743482626
H	0.2685330162	-2.5209745646	0.2570963497
C	1.5523394295	1.0976843272	-0.2980064751
H	-0.5076267037	1.6217390923	-0.5740409144
C	2.439227867	0.0589182586	-0.0293677588
H	2.7115924782	-2.0392373622	0.3807040566
H	1.9201233368	2.1048658949	-0.4621806576
Cl	4.150537838	0.4025854274	0.0527250393

1b-ci
E(RM062X) = -977.030279388
Sum of electronic and thermal Free Energies = -976.887783
Charge = 0 Multiplicity = 1 NIMAG = 0

C	4.1205830954	-0.2533588628	-0.2009435924
C	3.0908211572	-1.2899511635	0.1306123697
N	2.7738565898	-0.1372365062	-0.7048299638
C	1.9380096962	0.9090626125	-0.3556359607
O	2.3463472015	2.0492637847	-0.1987533001
C	0.4822139619	0.5679542891	-0.2685534304
C	-0.3869365088	1.5118395744	0.2851667454
C	-0.0204554159	-0.6554154207	-0.7219431469
C	-1.7464105047	1.2406870298	0.3974821985
H	0.0109484511	2.4606596279	0.6303854201
C	-1.3795487357	-0.9380721322	-0.6221153945
H	0.6439095881	-1.3882641371	-1.1689276123
C	-2.2240023681	0.0151380512	-0.0597972209
H	-2.4249441582	1.9674025188	0.831524809
H	-1.7777173976	-1.88199875	-0.9785624366
H	4.8847359718	-0.465658436	-0.9423203784
H	4.400775136	0.4401131109	0.5894867873
H	3.1251219323	-2.2242783873	-0.4258937888
C	2.5342967947	-1.3827644021	1.5276735712
H	1.5128022625	-1.7752984079	1.523692602
H	3.1548800919	-2.058185212	2.123880935
H	2.5274551865	-0.4021535963	2.0152660747
Cl	-3.9307945879	-0.3360028651	0.0726392229

1b-co
E(RM062X) = -977.028365990
Sum of electronic and thermal Free Energies = -976.885987
Charge = 0 Multiplicity = 1 NIMAG = 0

C	-2.7612029453	-1.5750980864	0.446625334
C	-3.9132001525	-0.690640472	0.0678888583
H	-4.6730426361	-1.1215084763	-0.5803853668
N	-2.6217695056	-0.5547477552	-0.5717165954

C	-1.8255904048	0.5710624863	-0.532027594
O	-2.2762309836	1.6909158111	-0.7214134577
C	-0.3564894769	0.3350569126	-0.341841927
C	0.4489714216	1.4341174439	-0.0319716931
C	0.222899822	-0.9298834538	-0.4813466971
C	1.8187285574	1.2785255102	0.1524717295
H	-0.006949452	2.4141728013	0.06492736
C	1.5940059674	-1.0989554878	-0.3107010305
H	-0.3874569773	-1.7885645324	-0.7418169614
C	2.3729558519	0.0093059198	0.0094117535
H	2.4468606771	2.127623083	0.3999534471
H	2.0506816696	-2.0760251105	-0.4270577451
H	-2.7178598199	-2.6010406837	0.0924232335
H	-2.2905271435	-1.3810749966	1.4086559933
C	-4.3934974668	0.3624562856	1.0330831722
H	-4.8008548069	1.227581948	0.5044010661
H	-5.1794724909	-0.0604142933	1.6658738279
H	-3.5782118808	0.7062611574	1.6763170816
Cl	4.0942013258	-0.1973936111	0.233041581

1c-ti
E(RM062X) = -517.461014458
Sum of electronic and thermal Free Energies = -517.307272
Charge = 0 Multiplicity = 1 NIMAG = 0

C	3.0762129555	-0.885884747	-0.6519020119
C	4.0667557678	0.1312730675	-0.1770004511
H	4.262850963	0.9963767349	-0.8049984966
H	4.8862096784	-0.1943332793	0.4579461187
N	2.7527060255	0.0499366954	0.4187236067
C	1.8388683204	1.0874485245	0.2745230748
O	2.1795879506	2.2590411263	0.2664499734
C	0.4024753198	0.6723286305	0.1979556825
C	-0.5421139903	1.6235523043	-0.2002110075
C	-0.0076943355	-0.6271965348	0.5147106977
C	-1.8868354728	1.2755194957	-0.2915954397
H	-0.2095862584	2.6292210862	-0.4378207907
C	-1.3552129329	-0.9711039588	0.4284783082
H	0.7217569096	-1.3598917568	0.8454483923
C	-2.2942863988	-0.0223719634	0.0223608947
H	-2.6172618303	2.0145260159	-0.6062211546
H	-1.6726285134	-1.9777701756	0.6825092318
C	3.2004037211	-2.340075143	-0.2892440971
H	2.221902231	-2.8302626561	-0.2871659032
H	3.8361100642	-2.8556060684	-1.0154097116
H	3.6467390436	-2.4451180118	0.7039280276
H	2.6051498111	-0.6654765745	-1.6092192426
H	-3.3436880393	-0.2931791417	-0.0463297018

1c-to
E(RM062X) = -517.460277380
Sum of electronic and thermal Free Energies = -517.306597
Charge = 0 Multiplicity = 1 NIMAG = 0

C	-3.8850911119	0.219945352	0.4321205845
C	-2.805000779	1.1480066055	0.8973548375
H	-4.0492122739	-0.6622072446	1.0490659374
H	-2.2818314349	0.9007807986	1.8179625032

H	-2.8682127826	2.2052702507	0.6529333654
C	-5.0760082586	0.7263756039	-0.3367075814
H	-5.4545878168	-0.0468390025	-1.0117482302
H	-5.8800567675	1.0086828574	0.3492458976
H	-4.799555639	1.6018375445	-0.9314561978
N	-2.6040093716	0.2568219742	-0.2352488042
C	-1.732541521	-0.8246798505	-0.214937374
O	-2.1280094029	-1.9756676041	-0.304423713
C	-0.275440015	-0.4839300135	-0.1538393057
C	0.633892879	-1.5124101184	0.1125604069
C	0.1882201233	0.8198378632	-0.3611309022
C	1.9971227938	-1.2385827851	0.1806644701
H	0.2598120031	-2.519844252	0.2661232043
C	1.5538372929	1.0893668152	-0.3009648073
H	-0.5139182574	1.6169372215	-0.5846591151
C	2.4581134308	0.0626337379	-0.0266332889
H	2.7001248819	-2.0380406473	0.3933173076
H	1.9122122274	2.099938733	-0.4709478888
H	3.5217617098	0.2758883405	0.023013804

1c-ci
E(RM062X) = -517.460579644
Sum of electronic and thermal Free Energies = -517.306460
Charge = 0 Multiplicity = 1 NIMAG = 0

C	4.1223336162	-0.2599990195	-0.2043828722
C	3.089452176	-1.2907146474	0.1350238567
N	2.7758547703	-0.1439503008	-0.7090678138
C	1.9402297728	0.9045804341	-0.3581488743
O	2.3543896839	2.0425319728	-0.1983508292
C	0.4841033514	0.5656531844	-0.2715208506
C	-0.3818426207	1.5057144695	0.2955177393
C	-0.0196377686	-0.6535360151	-0.7376552072
C	-1.7413554722	1.226021963	0.4045068649
H	0.0233896248	2.4486538876	0.6490705511
C	-1.3816651629	-0.9280241509	-0.6341254154
H	0.6481407604	-1.3786826447	-1.192937692
C	-2.242242428	0.0088915217	-0.0605483413
H	-2.4103591904	1.9555466075	0.8504863019
H	-1.7717965814	-1.8712379521	-1.0039314536
H	4.8859433009	-0.4796706784	-0.9442615158
H	4.405159633	0.4376393043	0.5814489739
H	3.1206880678	-2.2306435845	-0.4121811985
C	2.5346861777	-1.3668178116	1.5338455334
H	1.5116553836	-1.7552431969	1.5354738654
H	3.1538064926	-2.0369984154	2.1375363039
H	2.5320517196	-0.379905762	2.0088213422
H	-3.3031362066	-0.2080964457	0.0215400109

1c-co
E(RM062X) = -517.458661781
Sum of electronic and thermal Free Energies = -517.304245
Charge = 0 Multiplicity = 1 NIMAG = 0

C	-2.7599096099	-1.5733293243	0.4531759859
C	-3.9140908144	-0.6919590352	0.0735875043
H	-4.6780408323	-1.1278900952	-0.5665533139
N	-2.6261003287	-0.5616614636	-0.5737737171

C	-1.8286835502	0.5656043484	-0.5441050106
O	-2.2840765333	1.6820606305	-0.7439433001
C	-0.3597779565	0.332811908	-0.3493212335
C	0.4409813534	1.4325955973	-0.0261516915
C	0.2219429966	-0.9312115094	-0.4969337245
C	1.8107128972	1.2690578657	0.1626468056
H	-0.0226220618	2.4089277664	0.0751729186
C	1.5947628004	-1.0900594924	-0.3192819487
H	-0.3905393608	-1.7854446248	-0.7682561085
C	2.3890063587	0.0072731939	0.0157130411
H	2.4273443499	2.1244261845	0.4211008744
H	2.0447170531	-2.070073272	-0.4447764865
H	-2.7181996311	-2.6022120287	0.1073750733
H	-2.2840886222	-1.3719574546	1.4112064631
C	-4.389713199	0.3685262347	1.0331800661
H	-4.8016186788	1.228770405	0.4999757376
H	-5.171006144	-0.049729009	1.6748773042
H	-3.5707757929	0.7191495517	1.668048766
H	3.4579925667	-0.1201948168	0.1583428845

1d-ti
E(RM062X) = -631.947169087
Sum of electronic and thermal Free Energies = -631.763647
Charge = 0 Multiplicity = 1 NIMAG = 0

C	3.1010034715	-0.8980489904	-0.6370037303
C	4.0750949191	0.1327761659	-0.1605653801
H	4.2747724118	0.9904115825	-0.7974798128
H	4.8870228753	-0.1775087515	0.4916303888
N	2.7522866575	0.0496479976	0.4162098596
C	1.8350782388	1.083067051	0.2308365201
O	2.1802825235	2.2538374319	0.1869801594
C	0.4068895436	0.6655396998	0.1593149848
C	-0.5482483244	1.6059156606	-0.2292860009
C	-0.009675329	-0.6373940003	0.4731012897
C	-1.8967013969	1.270214878	-0.3182486481
H	-0.2265000388	2.615103984	-0.4675592128
C	-1.3475113437	-0.9854967544	0.3940985623
H	0.716242197	-1.3749272793	0.8006203347
C	-2.2990570144	-0.0339520808	-0.0045907189
H	-2.6152753862	2.0195799147	-0.6278790164
H	-1.6835975034	-1.9869239872	0.6434160566
C	3.2319758691	-2.3468978846	-0.2551220807
H	2.2585085409	-2.8470989391	-0.2656455667
H	3.8863393868	-2.8642801713	-0.9632820154
H	3.6606961629	-2.4362431694	0.7472887075
H	2.643744717	-0.694491132	-1.6048665697
O	-3.5791581427	-0.4643406767	-0.0534721237
C	-4.5827812018	0.4708124323	-0.4284338247
H	-4.4144361908	0.8424245304	-1.444825463
H	-5.5260319245	-0.0733954953	-0.3927851652
H	-4.6184430083	1.3108427737	0.2734794659

1d-to
E(RM062X) = -631.946501104
Sum of electronic and thermal Free Energies = -631.763131
Charge = 0 Multiplicity = 1 NIMAG = 0

C	-3.8910038573	0.2310481555	0.4308642945
C	-2.8174178072	1.1782642199	0.8699085668
H	-4.050408323	-0.6337990048	1.0730209986
H	-2.293598083	0.9604337475	1.7976481622
H	-2.8862168493	2.2280592266	0.5960915181
C	-5.0848346062	0.7066516429	-0.3530650871
H	-5.4557834243	-0.0877768774	-1.0075103512
H	-5.8928929005	1.0009146808	0.3231491676
H	-4.8144911273	1.5677201353	-0.9712419563
N	-2.6099478512	0.2565320934	-0.2373047321
C	-1.733265417	-0.8235912263	-0.1758349227
O	-2.1327801502	-1.9771853927	-0.2206511202
C	-0.2838992226	-0.4798143248	-0.1283196893
C	0.6366747134	-1.4997996175	0.1157163604
C	0.1869111599	0.8281460726	-0.3238242523
C	2.003190322	-1.2397638573	0.1770829448
H	0.272939351	-2.5122570018	0.2619010868
C	1.5432223348	1.1015832722	-0.2724929564
H	-0.510972049	1.63287146	-0.5327184534
C	2.4597556964	0.069429723	-0.0179050001
H	2.6941139618	-2.0507435989	0.3730046992
H	1.919733008	2.1071530541	-0.4306543627
O	3.7613837692	0.4307329429	0.0187294451
C	4.7278705391	-0.583874703	0.2619631724
H	5.6959071653	-0.0841420732	0.2442751325
H	4.5735225969	-1.0470038291	1.2425019836
H	4.6975600301	-1.3505241897	-0.5196120288

1d-ci
E(RM062X) = -631.946751582
Sum of electronic and thermal Free Energies = -631.762400
Charge = 0 Multiplicity = 1 NIMAG = 0

C	4.1298378802	-0.247749234	-0.2034731745
C	3.1041090773	-1.2901252644	0.1191771676
N	2.7833577557	-0.1332160882	-0.7093471227
C	1.9424692848	0.9065745083	-0.3315873741
O	2.3608745808	2.0391474376	-0.1387577039
C	0.4939256031	0.5641120414	-0.2586229507
C	-0.3876109194	1.4960362541	0.2902733236
C	-0.0121543628	-0.6618805125	-0.71797075
C	-1.7497436428	1.2260877411	0.3970731777
H	0.0038327097	2.4457597585	0.6417815506
C	-1.3644354464	-0.9433986687	-0.6236771101
H	0.6549425164	-1.393216622	-1.164437321
C	-2.241167823	-0.0022144626	-0.0615584038
H	-2.4093485401	1.9673593187	0.832202338
H	-1.7689625951	-1.8847375104	-0.9817606058
H	4.8966051772	-0.4509511139	-0.9448686218
H	4.4067367023	0.4402307944	0.5928833878
H	3.1417848981	-2.2219737673	-0.441454152
C	2.5479317415	-1.3920977652	1.515981024
H	1.5271215475	-1.7863894145	1.5095661763
H	3.1699794935	-2.0682735095	2.109950305
H	2.5379298511	-0.4133936591	2.0074446532
O	-3.5411899485	-0.3673330085	-0.0057705483
C	-4.468511006	0.5522630474	0.5568712813
H	-5.4417595511	0.0653231163	0.50284054

H	-4.2242457346	0.7659099151	1.6029958993
H	-4.4941776693	1.485172938	-0.0163651558

1d-co
E(RM062X) = -631.944629870
Sum of electronic and thermal Free Energies = -631.760431
Charge = 0 Multiplicity = 1 NIMAG = 0

C	-2.7545074215	-1.5833440288	0.4193417436
C	-3.9050804104	-0.6919930651	0.0549738259
H	-4.6688677821	-1.1119435779	-0.5960624801
N	-2.6151308588	-0.5515165536	-0.5870700031
C	-1.8170080038	0.5780980323	-0.5240607822
O	-2.2805508067	1.6973392594	-0.6945288515
C	-0.3542263837	0.3474571274	-0.3350411138
C	0.4595628328	1.4501464495	-0.0350576367
C	0.2361160536	-0.9108064876	-0.4651244339
C	1.8225832015	1.2950470962	0.1446890559
H	0.0032200866	2.4308227272	0.0562876204
C	1.6095938518	-1.0820307484	-0.2979739905
H	-0.37022483	-1.7746273241	-0.7193351404
C	2.4067968138	0.0252583719	0.0137646992
H	2.4594074147	2.1408977404	0.3837731476
H	2.0408567836	-2.0688908969	-0.4152100411
H	-2.7132406244	-2.6057002665	0.0541994826
H	-2.2814898569	-1.4020071358	1.3828719696
C	-4.382644907	0.3470705655	1.0372930664
H	-4.7926388447	1.2192969679	0.522502383
H	-5.1659298716	-0.0854326875	1.6671449445
H	-3.5657057774	0.6833739088	1.6823684458
O	3.744028779	-0.0318389323	0.2029884207
C	4.3833246579	-1.2949956462	0.0722566353
H	4.2563192751	-1.6970006986	-0.9386624361
H	5.4411936026	-1.1153735422	0.2616617053
H	3.9965360057	-2.0094241948	0.8069564133

1e-ti
E(RM062X) = -709.138723826
Sum of electronic and thermal Free Energies = -708.936975
Charge = 0 Multiplicity = 1 NIMAG = 0

C	3.0426558738	-0.9178022834	-0.6924899015
C	4.0707279350	0.0986924238	-0.2723568531
H	4.2610538807	0.9372497916	-0.9375738373
H	4.9051624652	-0.2365098485	0.3378409558
N	2.7806689891	0.0482418976	0.3626539260
C	1.8624462896	1.0876382593	0.2206551797
O	2.2129997893	2.2539603158	0.1753868215
C	0.4249982518	0.6749511913	0.1985565578
C	-0.5321867060	1.6317710663	-0.1539817961
C	0.0254009314	-0.6262525624	0.5232468954
C	-1.8805065564	1.2881288091	-0.1906489430
H	-0.2068763124	2.6380318263	-0.3990637442
C	-1.3256525338	-0.9650758441	0.4915090517
H	0.7636915291	-1.3676836591	0.8146204267
C	-2.2779966859	-0.0106799937	0.1322286491
H	-2.6212952000	2.0312100938	-0.4692841916
H	-1.6344669467	-1.9730300737	0.7505974651

H	2.5557035729	-0.7214688291	-1.6466822712
H	-3.3302143003	-0.2779509790	0.1062002968
C	3.1519141283	-2.3569932498	-0.3173185860
C	3.0225545284	-3.3368478100	-1.3052689683
C	3.3996836023	-2.7432628087	1.0042353135
C	3.1504984201	-4.6872902349	-0.9803329498
H	2.8203300696	-3.0400482628	-2.3314990672
C	3.5264128477	-4.0923552674	1.3283563507
H	3.4819493246	-1.9823562848	1.7764311464
C	3.4036559729	-5.0680830341	0.3370590812
H	3.0477955810	-5.4405719331	-1.7557588527
H	3.7168086092	-4.3835015811	2.3571481541
H	3.5006183195	-6.1190913253	0.5920332906

1e-to
E(RM062X) = -709.137041928
Sum of electronic and thermal Free Energies = -708.935640
Charge = 0 Multiplicity = 1 NIMAG = 0

C	-3.7803351145	0.7816797499	0.5099540788
C	-2.5799033780	1.6780256764	0.3552205242
H	-3.9341284979	0.3600196786	1.5016373789
H	-1.9672435801	1.8565796798	1.2358285820
H	-2.6137241349	2.4759477244	-0.3825459801
N	-2.5969369455	0.3325068068	-0.1814455911
C	-1.8168513878	-0.7087215450	0.3165356390
O	-2.3124630872	-1.6824391496	0.8562447711
C	-0.3450156406	-0.5834734426	0.0779291200
C	0.5077921660	-1.4750431149	0.7365068101
C	0.1863880902	0.3847133189	-0.7812878607
C	1.8839824879	-1.3943633692	0.5446781416
H	0.0802668846	-2.2238706989	1.3959289076
C	1.5638113617	0.4579099027	-0.9770888678
H	-0.4733866442	1.0673715751	-1.3077538118
C	2.4128705607	-0.4278122690	-0.3125919817
H	2.5439372644	-2.0846517160	1.0608779185
H	1.9739782290	1.2041619117	-1.6504168965
C	-5.0215911107	1.0050314878	-0.2880850529
C	-6.2546181267	1.0798598965	0.3652120495
C	-4.9710078442	1.1674843917	-1.6763840038
C	-7.4230590576	1.3202111691	-0.3574733266
H	-6.2988724127	0.9448681842	1.4431217390
C	-6.1384883228	1.4069278408	-2.3981744190
H	-4.0150410588	1.0930318374	-2.1883285599
C	-7.3677778501	1.4855578662	-1.7410443668
H	-8.3760629832	1.3735397133	0.1605194748
H	-6.0898169408	1.5274516956	-3.4764211810
H	-8.2770665630	1.6692645958	-2.3054377777
H	3.4863788369	-0.3668615775	-0.4646808076

1e-ci
E(RM062X) = -709.136353293
Sum of electronic and thermal Free Energies = -708.934048
Charge = 0 Multiplicity = 1 NIMAG = 0

C	4.1380826639	-0.3075944681	-0.2292129375
C	3.0822755331	-1.3059670578	0.1561427887
N	2.7911593958	-0.1669785131	-0.7091771653

C	1.9788369210	0.9178459063	-0.4136594613
O	2.4249210308	2.0523965699	-0.3420315361
C	0.5206636489	0.6170484439	-0.2720135969
C	-0.3108701322	1.6193407787	0.2368809687
C	-0.0184416374	-0.6256163348	-0.6219788762
C	-1.6719678701	1.3793057268	0.4025159859
H	0.1227522680	2.5781980565	0.5031204769
C	-1.3820248135	-0.8608836245	-0.4613296083
H	0.6210592366	-1.4022706844	-1.0303722489
C	-2.2083986279	0.1387306818	0.0534316334
H	-2.3141940559	2.1572282974	0.8037052445
H	-1.7997115013	-1.8237200713	-0.7387501523
H	4.8792710826	-0.5881481276	-0.9715165192
H	4.4688351619	0.4011883057	0.5266506250
H	3.0800428357	-2.2569102423	-0.3718250031
C	2.5233137044	-1.3818183808	1.5397765794
C	1.9675007102	-2.5918440640	1.9642168405
C	2.5116164107	-0.2865097071	2.4133029742
C	1.4169194683	-2.7142308948	3.2402188896
H	1.9653059487	-3.4430300901	1.2878131446
C	1.9556682068	-0.4062452823	3.6841347516
H	2.9322744794	0.6690778677	2.1097260004
C	1.4082608606	-1.6206455961	4.1032472011
H	0.9917303943	-3.6624925244	3.5551264469
H	1.9510039387	0.4515586883	4.3498239953
H	0.9769478423	-1.7101444754	5.0956133468
H	-3.2703647044	-0.0487848242	0.1815291718

1e-co
E(RM062X) = -709.133822851
Sum of electronic and thermal Free Energies = -708.931730
Charge = 0 Multiplicity = 1 NIMAG = 0

C	-2.8580262227	-1.4352389925	0.1443665028
C	-3.9146979515	-0.4099503849	-0.1801573161
H	-4.6652320129	-0.6851462030	-0.9173390433
N	-2.5730498253	-0.3158634144	-0.7140590488
C	-1.6829750937	0.7274227245	-0.5451921648
O	-2.0241205393	1.8899997846	-0.6922914997
C	-0.2633370168	0.3427322400	-0.2559305140
C	0.5907618212	1.3315039523	0.2426909637
C	0.2241359024	-0.9493609086	-0.4814063435
C	1.9190891225	1.0279870398	0.5287021337
H	0.2001950386	2.3315742102	0.4034298366
C	1.5575219578	-1.2464950954	-0.2073289761
H	-0.4271219492	-1.7167182277	-0.8883050259
C	2.4038059806	-0.2612619515	0.3028470881
H	2.5767465078	1.7960707103	0.9238794735
H	1.9362863919	-2.2465824606	-0.3942211093
H	-2.8927421196	-2.4060562644	-0.3422658738
H	-2.4443735996	-1.4202735702	1.1506939438
H	3.4412174290	-0.4970165601	0.5206864837
C	-4.4006981151	0.5398432323	0.8670422387
C	-5.7200941931	0.9938247139	0.7956069418
C	-3.5909605832	0.9747675833	1.9234990184
C	-6.2281528716	1.8618388903	1.7626608705
H	-6.3532654952	0.6650366297	-0.0244995436
C	-4.0959738803	1.8421430069	2.8879509242

H	-2.5576705446	0.6430138710	2.0022197667
C	-5.4169822857	2.2890594677	2.8114447006
H	-7.2561245226	2.2046400318	1.6920364282
H	-3.4556650933	2.1728270440	3.7002084158
H	-5.8076673063	2.9671798205	3.5640645482

P(Me)3
E(RM062X) = -460.997595762
Sum of electronic and thermal Free Energies = -460.913376
Charge = 0 Multiplicity = 1 NIMAG = 0

P	-1.6897330797	2.4034647164	-0.8636808965
C	-0.8101051669	3.2174611237	0.5461446864
H	-1.1016725573	4.2707388144	0.602905544
H	-1.0981720321	2.736237962	1.48589063
H	0.2783867908	3.1548448706	0.433195796
C	-0.8126684215	3.2192742575	-2.2740540775
H	-1.1024777057	2.7392962733	-3.2139001024
H	-1.1043063176	4.2726325424	-2.3289014797
H	0.2760273863	3.1564771809	-2.1631895395
C	-0.8076183672	0.7770879242	-0.8655100488
H	-1.0958242592	0.2016923461	0.0196513348
H	-1.0973598465	0.2028316811	-1.7509095451
H	0.2806266867	0.9081373273	-0.8663696716

PCyPh2
E(RM062X) = -1039.63406280
Sum of electronic and thermal Free Energies = -1039.335352
Charge = 0 Multiplicity = 1 NIMAG = 0

P	-2.0317484314	0.766618241	-0.0687174608
C	-1.1926105287	1.6857423725	-1.4596061879
C	-1.5892347672	1.0743247124	-2.8118646965
C	-1.5807273319	3.1718604853	-1.4148487094
H	-0.1017244735	1.6194532065	-1.3313281987
C	-0.9534235783	1.8405680284	-3.9757311593
H	-2.6842270693	1.1163509023	-2.907569085
H	-1.3087210892	0.0159846365	-2.8578110158
C	-0.9517862663	3.9413451838	-2.581013409
H	-2.6762013035	3.2601241075	-1.469068308
H	-1.2695640656	3.6176807429	-0.4632910719
C	-1.3274053911	3.3233293292	-3.9292687702
H	-1.2662275016	1.396649511	-4.9275236243
H	0.1398009943	1.7394385997	-3.9190880809
H	-1.2630510284	4.991326854	-2.5419972377
H	0.1415369424	3.9280848709	-2.469235832
H	-0.8377981549	3.8659811101	-4.7457193009
H	-2.4112371659	3.4243316252	-4.0819296773
C	-1.2609565482	1.5989004914	1.3926768521
C	-2.0735313164	2.4066610623	2.1966484301
C	0.1013606332	1.4900972986	1.702852641
C	-1.5372476475	3.1002039289	3.2836934825
H	-3.1335459011	2.4955763273	1.9700545351
C	0.6385946417	2.1790974226	2.7885417327
H	0.747554118	0.8595940326	1.0959723138
C	-0.1804458923	2.9868485602	3.5807821563
H	-2.1804318916	3.7236162565	3.8979198751
H	1.6959557951	2.0846624962	3.0186732077

H	0.2389218158	3.5217407576	4.4278263715
C	-1.147714274	-0.8568533464	-0.0353778191
C	-1.724461311	-1.8660910028	0.7495113399
C	0.0384306247	-1.1438934168	-0.7235110778
C	-1.1247813632	-3.1193502703	0.8625978728
H	-2.6534203322	-1.6669285534	1.2800190411
C	0.633740911	-2.402315832	-0.6226712583
H	0.5123447016	-0.3904988923	-1.3463520026
C	0.0569852631	-3.3915615928	0.1728763398
H	-1.5850862775	-3.884200628	1.4811963256
H	1.5520490037	-2.6066357097	-1.1657110363
H	0.5226937773	-4.3694637589	0.2512044626

TS1a-C2-to
E(RM062X) = -1761.49548630
Sum of electronic and thermal Free Energies = -1761.026016
Charge = 0 Multiplicity = 1 NIMAG = 1

C	0.2276747094	0.1608705039	0.3402399666
C	1.1360918392	0.5676112389	1.417440315
H	2.1635939652	0.7872573771	1.1200511483
H	1.0649822042	-0.0341358781	2.3252335177
N	0.2354969118	1.7136718186	1.4489740201
C	0.6582207611	2.7535609098	0.6805536302
O	1.6833902624	2.7909353296	-0.0143112387
C	-0.2562052096	3.9581073274	0.7119644815
C	0.1309490123	5.1077455134	0.0158350197
C	-1.467140501	3.9447495521	1.4134797134
C	-0.6745512621	6.2403196769	0.0155077183
H	1.0715145701	5.1016881289	-0.5240276762
C	-2.2893080219	5.065676146	1.4206326563
H	-1.7580259159	3.0522992861	1.9553207276
C	-1.872548758	6.1932573543	0.7208572693
H	-0.3861267879	7.1388643286	-0.5168389675
H	-3.2302965608	5.071136908	1.9574191234
N	-2.7333260286	7.3839133608	0.7285074567
O	-3.7833202629	7.3291789924	1.3491381074
O	-2.3562055838	8.3690561346	0.1139710731
H	0.3974654665	0.6258277375	-0.6260264776
C	-1.0890285626	-0.5187166231	0.601840443
H	-1.8594125659	0.2328726383	0.7929325052
H	-1.3999407487	-1.1034116163	-0.2671698417
H	-1.023483106	-1.178573437	1.4720897526
P	1.4705187798	-1.9279417386	-0.448954112
C	1.1979999583	-2.7886604124	-2.0449320791
C	1.2346888157	-3.2207186394	0.8220065485
C	0.6746845391	-2.0327847577	-3.1017017286
C	1.5103489504	-4.138704984	-2.2610358142
C	0.12119267	-4.0717928926	0.7370503188
C	2.042788611	-3.2825592911	1.9659374916
C	0.4790904432	-2.6088482511	-4.3578118092
H	0.4188969074	-0.9872850026	-2.9430507616
C	1.3071298418	-4.7149215424	-3.5129266482
H	1.912421536	-4.7413195283	-1.4505021148
C	-0.1616922142	-4.9753898269	1.7585758116
H	-0.5274277087	-4.0329112053	-0.135179862
C	1.7556885266	-4.1867957039	2.9890970481
H	2.9054053398	-2.6301322258	2.0688441818

C	0.7935357608	-3.9507419212	-4.5632414716
H	0.0755214055	-2.0108929413	-5.16931147
H	1.5523788053	-5.7611140778	-3.6701034906
C	0.6557478022	-5.0367224289	2.888323552
H	-1.0233728602	-5.6306600286	1.6723089189
H	2.3966640328	-4.2250001695	3.8649018114
H	0.6365437351	-4.4034780581	-5.5377374865
H	0.4346866203	-5.740722171	3.6847337281
C	3.2805942479	-1.5127187549	-0.4338096195
C	4.2323559121	-2.6754071608	-0.7337189862
C	3.5295451598	-0.3349471446	-1.3905970851
H	3.4706832737	-1.1495770714	0.5874949421
C	5.6910330916	-2.2119996133	-0.6476892436
H	4.0334172205	-3.0455430811	-1.7487180757
H	4.0541266792	-3.5092939241	-0.0446139841
C	4.9901842088	0.1208416247	-1.315441693
H	3.3003454759	-0.6517397252	-2.4185860761
H	2.8619734431	0.5017336502	-1.1480761282
C	5.9529986439	-1.0360847602	-1.591636776
H	6.3593157023	-3.0475799276	-0.8840339298
H	5.912665106	-1.9066832877	0.3847067588
H	5.1591674188	0.9378014489	-2.0254435721
H	5.1843458779	0.5248996164	-0.3117581956
H	6.9902525809	-0.6962936869	-1.4961128029
H	5.820725833	-1.3739361128	-2.6291665392

TS1a-C2-ci
E(RM062X) = -1761.49151331
Sum of electronic and thermal Free Energies = -1761.021647
Charge = 0 Multiplicity = 1 NIMAG = 1

C	0.0879759353	0.2527391876	0.0199081499
C	0.0766870279	0.0743860008	1.4824724488
N	1.5231147102	0.1375890526	0.2093347844
C	2.0539904868	-1.0631947385	-0.1311990949
O	1.4766800504	-2.0075146508	-0.6881789271
C	3.5196890606	-1.2125946706	0.2204232964
C	4.1861570011	-2.3751903859	-0.1805200147
C	4.2092459055	-0.2322162358	0.9432358608
C	5.527692359	-2.5658765217	0.1279589919
H	3.6373738119	-3.1270295204	-0.7370605816
C	5.5501507213	-0.406319744	1.266347226
H	3.6879686298	0.6676067348	1.2484734835
C	6.1826757899	-1.572882949	0.848972979
H	6.0570008537	-3.4599940504	-0.1787196717
H	6.0967096528	0.3428293375	1.8265631085
N	7.6003221599	-1.7642460984	1.1833529783
O	8.163785468	-0.8883410863	1.8207247822
O	8.1455189675	-2.789944473	0.8074260033
H	-0.2156962023	1.2402162297	-0.3320741359
H	-0.3821216773	-0.5545312502	-0.545668507
H	0.3885011452	0.9261240042	2.0775382865
C	0.1228187444	-1.2883433359	2.1261209662
H	-0.1387628947	-2.0687966947	1.4091300328
H	1.1277033919	-1.4860090227	2.5130299017
H	-0.5622630123	-1.3418833604	2.9746697491
P	-2.3442627452	0.7109163852	1.9542081115
C	-3.3205577156	0.1041576	0.5286778234

C	-3.2257264759	-0.0093388903	3.3935008438
C	-3.1601338228	-1.2477650601	0.1873139389
C	-4.1865270091	0.8964268803	-0.2334205053
C	-2.5156194914	-0.152388254	4.593911366
C	-4.5682886441	-0.4060371295	3.3439907239
C	-3.8581666374	-1.7979937945	-0.8841898586
H	-2.4870338955	-1.8756760854	0.7683571472
C	-4.8775386139	0.3463070568	-1.3149244676
H	-4.3349487106	1.9451670091	0.0057500273
C	-3.1395306139	-0.6684662806	5.728988912
H	-1.4699559666	0.1453790632	4.6435218675
C	-5.189522875	-0.9268174453	4.478626471
H	-5.1315719541	-0.3098874776	2.4193308445
C	-4.7170365157	-0.9988613099	-1.6410804257
H	-3.7253970986	-2.8465524692	-1.1327060338
H	-5.5441512371	0.9733273459	-1.8995928162
C	-4.4775827603	-1.0580629785	5.6714502719
H	-2.5791442259	-0.7719739822	6.6532298585
H	-6.2310268352	-1.2304614983	4.4297749859
H	-5.2556663918	-1.4233875809	-2.4829081426
H	-4.9634270689	-1.4667339991	6.5523541539
C	-2.692864708	2.5337890177	2.0630511483
C	-2.2921423529	3.0909478515	3.4377390512
C	-1.9342308798	3.2741287936	0.9482133134
H	-3.7759657294	2.6805532247	1.9379344972
C	-2.5371902966	4.6021691053	3.5046679182
H	-1.2257622672	2.8859655415	3.6169729746
H	-2.8553822653	2.5901622794	4.2317184436
C	-2.1969016505	4.7811557618	1.0138150041
H	-0.8569846652	3.0930200332	1.0769807199
H	-2.2051684328	2.8831233512	-0.038922697
C	-1.8106204233	5.3447994684	2.3821835886
H	-2.2206786356	4.9813053817	4.4825388813
H	-3.6167304297	4.7903721083	3.4231448267
H	-1.6387389006	5.2873668474	0.2187430931
H	-3.2635992382	4.9701982463	0.8281807763
H	-2.03407357	6.4161839344	2.430150538
H	-0.7255993166	5.2372052714	2.5207361893

TS1a-C2-co
E(RM062X) = -1761.48155892
Sum of electronic and thermal Free Energies = -1761.010800
Charge = 0 Multiplicity = 1 NIMAG = 1

C	-0.0014179188	-0.0643093979	0.0046374763
C	-0.0150284769	-0.0569559159	1.4853487873
H	0.9512766472	-0.0554517435	1.9778983707
N	0.3420671225	-1.4537468004	0.1985413267
C	-0.5960736769	-2.41245149	0.0179038095
O	-0.5583124285	-3.5082499522	0.5910159981
C	-1.7319157384	-2.1905689001	-0.967713727
C	-3.0205888092	-2.6003380504	-0.6125411928
C	-1.5031023354	-1.6356269642	-2.2314596215
C	-4.0830109465	-2.4379644223	-1.4943572884
H	-3.186234255	-3.0492057107	0.3618878425
C	-2.5472478983	-1.4863477059	-3.1370931497
H	-0.5007903917	-1.3246375413	-2.5121609458
C	-3.8208737967	-1.8832741019	-2.7429438498

H	-5.0892185852	-2.7394300535	-1.2284334425
H	-2.3833177535	-1.068383244	-4.1232191905
N	-4.9341834519	-1.7122276581	-3.6854517041
O	-4.6871997404	-1.250022566	-4.7881378186
O	-6.0521788969	-2.0382290582	-3.3186508991
H	0.7957511344	0.5250685446	-0.4549004414
H	-0.9714964615	0.1646541703	-0.4467392019
C	-1.2023878875	-0.5784503398	2.2554618985
H	-1.0144476579	-1.6083337557	2.5765003209
H	-1.3919850302	0.0173887869	3.1505307553
H	-2.1023915502	-0.5713528113	1.6319674112
P	-0.2386346893	2.3940706153	1.9045952052
C	-1.2749117291	3.0621056948	0.5500423782
C	-1.087224163	2.9944264781	3.4155684468
C	-2.5048471607	2.4248633983	0.3224960042
C	-0.9114693393	4.1405111674	-0.2643819931
C	-0.8217727268	2.3350292595	4.6243718957
C	-1.9762131631	4.0769211418	3.4100676929
C	-3.3546388203	2.8589710751	-0.6911007286
H	-2.8013946738	1.5850725412	0.9478393254
C	-1.7604181232	4.5693858024	-1.286935091
H	0.0302900027	4.6593515431	-0.1123642639
C	-1.4226780848	2.7585686911	5.8087039464
H	-0.1379968933	1.4885333597	4.6416994923
C	-2.5813823672	4.495111695	4.5949268952
H	-2.1992174749	4.595888633	2.4815008067
C	-2.9807922556	3.9323552048	-1.5020821875
H	-4.3043748154	2.3572762145	-0.8510373258
H	-1.4647869834	5.4063196977	-1.9125360894
C	-2.3058220048	3.8383802819	5.7944092516
H	-1.2064304863	2.2410137555	6.7383953033
H	-3.2694209477	5.3351316806	4.5792355193
H	-3.6385529704	4.2685276182	-2.2978544846
H	-2.7811481919	4.1647029112	6.7145576433
C	1.3537107293	3.3497123768	1.8404864721
C	2.145693896	3.201932451	3.1488325874
C	2.1985163185	2.8638706877	0.6504859146
H	1.1043989811	4.4131201589	1.7109910725
C	3.4768737458	3.9568346064	3.0648498113
H	2.3417083227	2.1356932065	3.3378096874
H	1.560767779	3.5785800602	3.9939197563
C	3.5186972824	3.6347082017	0.5657907274
H	2.4159949856	1.7946021059	0.7881990528
H	1.643353454	2.9565709684	-0.2898830823
C	4.3124341266	3.5039593778	1.8665091187
H	4.0358090625	3.8161884897	3.9964657282
H	3.269539702	5.0322600803	2.9756070796
H	4.1068179303	3.2659906591	-0.2815721698
H	3.3051762574	4.6953875876	0.3722372168
H	5.2381795829	4.0868166545	1.8086609195
H	4.6031955826	2.4531481565	2.0047578403

TS1a-C2-ti
E(RM062X) = -1761.49553227
Sum of electronic and thermal Free Energies = -1761.025505
Charge = 0 Multiplicity = 1 NIMAG = 1

C	-0.0215490953	-0.0070317982	0.0188702727

C	-0.0301809056	-0.0116593425	1.4858226156
H	0.9472724767	-0.02220547	1.9724869794
H	-0.7461317712	-0.7026638521	1.9341272885
N	-0.6084293376	1.308771597	1.2672157768
C	0.3155031068	2.3071778121	1.2576154672
O	1.5393513792	2.1829560465	1.4059744761
C	-0.2632946617	3.6859595207	1.0296570581
C	0.6040895464	4.7830468929	1.0421055462
C	-1.6304571834	3.8830059219	0.8039826024
C	0.122076496	6.0694533537	0.8321642286
H	1.6607562682	4.6135699157	1.2178616317
C	-2.1314341161	5.1616581164	0.588246979
H	-2.2975474389	3.0288821529	0.8010507349
C	-1.2413612803	6.2304702379	0.608105268
H	0.7820545506	6.9286317697	0.8397228075
H	-3.1867670691	5.3309805389	0.4110996598
N	-1.7635742066	7.5853469021	0.3837474366
O	-2.9604341792	7.7126514904	0.1789975294
O	-0.9749799976	8.5168085187	0.4130366921
H	0.7890957012	0.5404022963	-0.4519364537
C	-1.2315464378	-0.36822943	-0.7993446272
H	-1.8779648916	0.5068537711	-0.9058153603
H	-0.9369476854	-0.6923167277	-1.8002880778
H	-1.8047770901	-1.1666915575	-0.3188005997
P	1.2487794723	-2.1885576576	-0.3788496133
C	2.0258765471	-2.6840463195	-1.9637650738
C	0.1346752014	-3.5747533186	0.0440706665
C	2.4153917622	-1.6607191349	-2.8373220797
C	2.2674709422	-4.0176137376	-2.3238614134
C	-0.7382700898	-4.0733200854	-0.9362243733
C	0.0145728106	-4.0499316091	1.3571936724
C	3.0488652764	-1.9610203953	-4.0441285663
H	2.222854873	-0.6228462311	-2.5736740162
C	2.8919298353	-4.3166081298	-3.5326745271
H	1.9677377517	-4.8237713183	-1.6590667128
C	-1.6898809655	-5.0380732119	-0.615059485
H	-0.6714745999	-3.7084920067	-1.9588347337
C	-0.9418183659	-5.0145027043	1.6771252401
H	0.6673787758	-3.6755748356	2.140900816
C	3.2853054201	-3.2892867021	-4.3932016813
H	3.3484481927	-1.1584620924	-4.711266307
H	3.0744906652	-5.3526371946	-3.8023599972
C	-1.7940098084	-5.5130524224	0.693490997
H	-2.3523834201	-5.4170867232	-1.387514329
H	-1.016382072	-5.3764091728	2.6983136771
H	3.7717254829	-3.5262271692	-5.3347240405
H	-2.5361449941	-6.2649372651	0.9438384233
C	2.6246711167	-2.2443338774	0.8665079579
C	3.4573220408	-3.5308606327	0.8766821014
C	3.5225815192	-1.0097294424	0.6804240037
H	2.1163684763	-2.1393990791	1.8371978692
C	4.5234209864	-3.4727476924	1.9764912562
H	3.952910686	-3.6407211118	-0.0976101635
H	2.8144828646	-4.4084804102	1.0123223751
C	4.5973991741	-0.9565135849	1.7709003154
H	4.0079256521	-1.0644818118	-0.3051470495
H	2.9222247537	-0.0912660493	0.7006014239
C	5.4219246952	-2.2450731704	1.8088657414

H	5.1229480245	-4.3897900667	1.9601119064
H	4.0285539616	-3.4305467645	2.9571081358
H	5.2476450753	-0.0902464798	1.6069496995
H	4.1079208856	-0.8085446925	2.7440154475
H	6.1581814852	-2.199888526	2.619046387
H	5.9846444093	-2.3393279099	0.8694034143

TS1a-C3-to
E(RM062X) = -1761.49829016
Sum of electronic and thermal Free Energies = -1761.027998
Charge = 0 Multiplicity = 1 NIMAG = 1

C	0.8745901627	0.2457582763	0.0408766278
C	0.1046309275	-0.0259483198	1.2607974731
H	1.9404549352	0.4027503069	0.2137160038
H	0.4367203021	0.4044506049	2.1996818989
H	-0.9238430773	-0.3553013122	1.1725125107
C	0.5756566677	-0.5899012745	-1.1804385548
H	0.9579449044	-0.0923827214	-2.0765452723
H	1.0583855085	-1.5700729577	-1.0985313788
H	-0.5034265282	-0.7332115499	-1.2967859604
N	0.0740593882	1.4652052066	0.1350429966
C	0.6924555909	2.4945797541	0.7800951545
O	1.8178774401	2.4751316555	1.2939755561
C	-0.1264934174	3.7649564958	0.8521875274
C	0.4368013097	4.8832552486	1.4753334197
C	-1.4177147161	3.8432441854	0.3182300747
C	-0.274165538	6.0732884938	1.5721254944
H	1.4389877665	4.8067075426	1.8825066211
C	-2.1460094197	5.0241381902	0.4073648838
H	-1.8462405077	2.9743261032	-0.1674334605
C	-1.5562900501	6.1167025018	1.0346060573
H	0.1496794224	6.9477393727	2.050985447
H	-3.1472380204	5.1008474646	0.0007205019
N	-2.319222939	7.3684172297	1.1341144243
O	-3.4381598281	7.3993640234	0.6466308532
O	-1.7967220228	8.3154351602	1.7003277754
P	0.8016756193	-2.2859676148	2.0801599771
C	2.4468215819	-2.6615561029	1.371430585
C	1.1208122568	-2.3360818805	3.8850421561
C	3.4327278598	-1.671229488	1.503889076
C	2.7620419779	-3.845923418	0.6956580808
C	0.3523889515	-1.5106290678	4.7149261715
C	2.0753317458	-3.1869865131	4.4574303076
C	4.7069502109	-1.8636614299	0.9772302902
H	3.2033681639	-0.7458184426	2.0296397034
C	4.0381347629	-4.0336482348	0.1605325156
H	2.0239885423	-4.6346961853	0.5835243966
C	0.5269151713	-1.5416931563	6.0989038477
H	-0.3870680126	-0.8418279431	4.2795118394
C	2.250878681	-3.2147073563	5.8396282522
H	2.6853450671	-3.8254944328	3.8227269136
C	5.0111028512	-3.0458120368	0.2991512352
H	5.4594999515	-1.0893238907	1.091808545
H	4.2694917484	-4.9573113766	-0.3617121199
C	1.4767117513	-2.3931971501	6.661487411
H	-0.074011007	-0.8976739986	6.733832467
H	2.9933804207	-3.8765373526	6.2755198169

H	6.0024122594	-3.1947616218	-0.1180956859
H	1.6183517479	-2.4142406812	7.7378844231
C	-0.2449381705	-3.7873702479	1.7581829659
C	-1.4634758126	-3.8126139047	2.6941473144
C	-0.6996045801	-3.7982728372	0.2893020107
H	0.3663851415	-4.6783224068	1.9657029439
C	-2.3563568645	-5.0209000198	2.3933263158
H	-2.0437639424	-2.8868341426	2.56093873
H	-1.1395910368	-3.8414808087	3.739701047
C	-1.5805784075	-5.0154369217	-0.0048575804
H	-1.2754766835	-2.88173998	0.0942321761
H	0.1607347975	-3.779457407	-0.388982217
C	-2.7924598637	-5.0530369121	0.9276007664
H	-3.2309685769	-5.0030417287	3.0528210215
H	-1.7999909161	-5.9396671839	2.6253876829
H	-1.9024174103	-4.9931626457	-1.0517090739
H	-0.9876608035	-5.9310983247	0.1294259824
H	-3.3965634537	-5.9456327677	0.7315737246
H	-3.4308259815	-4.182450067	0.7210873095

TS1a-C3-to
E(RM062X) = -1761.49829016
Sum of electronic and thermal Free Energies = -1761.027998
Charge = 0 Multiplicity = 1 NIMAG = 1

C	0.8745901627	0.2457582763	0.0408766278
C	0.1046309275	-0.0259483198	1.2607974731
H	1.9404549352	0.4027503069	0.2137160038
H	0.4367203021	0.4044506049	2.1996818989
H	-0.9238430773	-0.3553013122	1.1725125107
C	0.5756566677	-0.5899012745	-1.1804385548
H	0.9579449044	-0.0923827214	-2.0765452723
H	1.0583855085	-1.5700729577	-1.0985313788
H	-0.5034265282	-0.7332115499	-1.2967859604
N	0.0740593882	1.4652052066	0.1350429966
C	0.6924555909	2.4945797541	0.7800951545
O	1.8178774401	2.4751316555	1.2939755561
C	-0.1264934174	3.7649564958	0.8521875274
C	0.4368013097	4.8832552486	1.4753334197
C	-1.4177147161	3.8432441854	0.3182300747
C	-0.274165538	6.0732884938	1.5721254944
H	1.4389877665	4.8067075426	1.8825066211
C	-2.1460094197	5.0241381902	0.4073648838
H	-1.8462405077	2.9743261032	-0.1674334605
C	-1.5562900501	6.1167025018	1.0346060573
H	0.1496794224	6.9477393727	2.050985447
H	-3.1472380204	5.1008474646	0.0007205019
N	-2.319222939	7.3684172297	1.1341144243
O	-3.4381598281	7.3993640234	0.6466308532
O	-1.7967220228	8.3154351602	1.7003277754
P	0.8016756193	-2.2859676148	2.0801599771
C	2.4468215819	-2.6615561029	1.371430585
C	1.1208122568	-2.3360818805	3.8850421561
C	3.4327278598	-1.671229488	1.503889076
C	2.7620419779	-3.845923418	0.6956580808
C	0.3523889515	-1.5106290678	4.7149261715
C	2.0753317458	-3.1869865131	4.4574303076
C	4.7069502109	-1.8636614299	0.9772302902

H	3.2033681639	-0.7458184426	2.0296397034
C	4.0381347629	-4.0336482348	0.1605325156
H	2.0239885423	-4.6346961853	0.5835243966
C	0.5269151713	-1.5416931563	6.0989038477
H	-0.3870680126	-0.8418279431	4.2795118394
C	2.250878681	-3.2147073563	5.8396282522
H	2.6853450671	-3.8254944328	3.8227269136
C	5.0111028512	-3.0458120368	0.2991512352
H	5.4594999515	-1.0893238907	1.091808545
H	4.2694917484	-4.9573113766	-0.3617121199
C	1.4767117513	-2.3931971501	6.661487411
H	-0.074011007	-0.8976739986	6.733832467
H	2.9933804207	-3.8765373526	6.2755198169
H	6.0024122594	-3.1947616218	-0.1180956859
H	1.6183517479	-2.4142406812	7.7378844231
C	-0.2449381705	-3.7873702479	1.7581829659
C	-1.4634758126	-3.8126139047	2.6941473144
C	-0.6996045801	-3.7982728372	0.2893020107
H	0.3663851415	-4.6783224068	1.9657029439
C	-2.3563568645	-5.0209000198	2.3933263158
H	-2.0437639424	-2.8868341426	2.56093873
H	-1.1395910368	-3.8414808087	3.739701047
C	-1.5805784075	-5.0154369217	-0.0048575804
H	-1.2754766835	-2.88173998	0.0942321761
H	0.1607347975	-3.779457407	-0.388982217
C	-2.7924598637	-5.0530369121	0.9276007664
H	-3.2309685769	-5.0030417287	3.0528210215
H	-1.7999909161	-5.9396671839	2.6253876829
H	-1.9024174103	-4.9931626457	-1.0517090739
H	-0.9876608035	-5.9310983247	0.1294259824
H	-3.3965634537	-5.9456327677	0.7315737246
H	-3.4308259815	-4.182450067	0.7210873095

TS1a-C3-ci
E(RM062X) = -1761.47896717
Sum of electronic and thermal Free Energies = -1761.006883
Charge = 0 Multiplicity = 1 NIMAG = 1

C	-0.0002627987	-0.0275396261	0.0648643864
N	0.0564741784	0.0910132787	1.506981016
C	1.1868863365	0.1441396509	2.2346015369
O	1.3391609269	0.8509059911	3.2408184889
C	2.3056312534	-0.8316570705	1.9053993163
C	3.6342929976	-0.4333625572	2.0783274286
C	2.019017358	-2.1363579464	1.4919108384
C	4.6762734183	-1.3125284272	1.8079932183
H	3.8456134337	0.5734434949	2.4237338964
C	3.0455036566	-3.0399175279	1.2409937223
H	0.9853796246	-2.4467440569	1.3696866401
C	4.3568739284	-2.6024076005	1.3942684247
H	5.711804457	-1.0139618152	1.9202255858
H	2.8394696176	-4.0569676453	0.9293800818
N	5.4494297451	-3.5424445543	1.1125750261
O	5.1569612358	-4.6791308855	0.7757652502
O	6.5967403791	-3.1406992331	1.2269406273
H	-0.6597853394	-0.837122334	-0.2664673898
C	1.2513317481	0.0566646704	-0.7936983978
H	1.7926148403	-0.8908282801	-0.8313934664

H	0.9687605695	0.32012779	-1.8192823654
H	1.9309573854	0.8272942059	-0.4104896159
H	-0.1109690204	2.1513322061	0.4323790575
H	-1.7214081242	1.2569819967	0.6511940614
C	-0.7145650988	1.2664623281	0.2536226333
P	-1.4819304652	2.1103871704	-1.8291577469
C	-1.4526093933	0.991725668	-3.2806397683
C	-0.5463082242	3.5852853997	-2.3720054503
C	-1.7972005858	-0.353951408	-3.0848240929
C	-1.0739491775	1.41852358	-4.55941501
C	0.8202382656	3.6354936957	-2.0683314091
C	-1.1259102042	4.6452652389	-3.082949012
C	-1.7839156138	-1.2502601515	-4.1512786445
H	-2.0751935318	-0.7043923262	-2.093645972
C	-1.0542866822	0.5170526861	-5.6242393301
H	-0.7886363766	2.4527804393	-4.7297713154
C	1.5982463311	4.7179358586	-2.4787466277
H	1.2815339855	2.8246853014	-1.5098385703
C	-0.3491755828	5.7303002007	-3.4848554865
H	-2.1835144857	4.6305643715	-3.3316169996
C	-1.4106384137	-0.8157993378	-5.4237204394
H	-2.0559561891	-2.2883632786	-3.9858081357
H	-0.7571457706	0.8594065703	-6.610996383
C	1.0137154426	5.7671132149	-3.1861086612
H	2.6569531224	4.7423248629	-2.2392794124
H	-0.8091020092	6.5460828109	-4.0344744071
H	-1.3915371215	-1.5155131367	-6.2538158649
H	1.6164369114	6.6134985544	-3.5014423048
C	-3.2450925262	2.6685505598	-1.6657093627
C	-4.1636683892	1.4728535836	-1.3678107613
C	-3.3454390255	3.7245245927	-0.5519398668
H	-3.5575800476	3.1112699763	-2.6223322562
C	-5.6096374935	1.933379892	-1.1557918921
H	-3.8101044721	0.9583822437	-0.4617425386
H	-4.1244894973	0.7495823288	-2.1887502438
C	-4.7951417402	4.1761101139	-0.3520619491
H	-2.9722907758	3.2908511597	0.3873222526
H	-2.7105168457	4.5873109468	-0.7803066153
C	-5.7084394206	2.9869206693	-0.0519674066
H	-6.2371491838	1.0675478295	-0.9180870189
H	-5.990180649	2.3568080224	-2.0956795443
H	-4.8411245879	4.9107006211	0.4591804226
H	-5.1433788377	4.6816303572	-1.263690082
H	-6.7453115634	3.322018942	0.0605715089
H	-5.4108066585	2.5369609141	0.905597667

TS1a-C3-co
E(RM062X) = -1761.48936588
Sum of electronic and thermal Free Energies = -1761.018937
Charge = 0 Multiplicity = 1 NIMAG = 1

C	-0.0298563861	-0.09385221	-0.0262430578
H	-0.0376305398	-0.1728929289	1.0625475794
N	1.374051521	-0.1289681902	-0.4612492639
C	1.6503070998	-0.419672897	-1.7624364162
O	0.8586110807	-0.5391905661	-2.7057002807
C	3.1334221436	-0.5849249476	-2.0398503559
C	3.5303487756	-0.8983331213	-3.3440706511

C	4.0996109432	-0.4340940764	-1.0387418747
C	4.8744672965	-1.0596942344	-3.6575774537
H	2.7701699176	-1.0125658746	-4.1089386365
C	5.449876054	-0.5866390144	-1.3329403938
H	3.7851529291	-0.1967864261	-0.0294187397
C	5.8089957452	-0.8976796163	-2.6401636392
H	5.1952613122	-1.3029891826	-4.6634201073
H	6.2095561892	-0.4708764169	-0.5692015946
N	7.233688349	-1.0614258781	-2.9590286182
O	8.045228031	-0.9015570854	-2.0608995462
O	7.5359614099	-1.3485310644	-4.1065290146
C	-1.0930813324	-0.9930337113	-0.6254324455
H	-0.828623782	-2.0400655316	-0.4448348681
H	-2.0459435444	-0.7946701448	-0.1211142731
H	-1.2083608743	-0.8484645288	-1.697599596
H	0.6521552296	2.0031547557	0.1543414083
H	0.0729528773	1.4972040432	-1.5437982291
C	0.1460093383	1.2885221463	-0.4821520456
P	-2.0349444305	2.501532081	-0.0991213153
C	-3.1786820161	2.537896994	-1.5310163335
C	-2.9547509273	1.6087926965	1.2131107344
C	-2.887246028	1.7177597688	-2.6275726106
C	-4.3144944511	3.3598988872	-1.5792185544
C	-2.2331372079	1.219396067	2.3527169591
C	-4.3002003696	1.2355853599	1.1054240072
C	-3.7207410158	1.7069018973	-3.7474374216
H	-2.0027645244	1.0863555406	-2.6131197188
C	-5.1431586249	3.3523511106	-2.698699389
H	-4.5614026591	4.0055655625	-0.7402696019
C	-2.8490237289	0.4968525836	3.372364258
H	-1.1793930498	1.475235892	2.4409249053
C	-4.9134780442	0.5049255861	2.1238952495
H	-4.8746832242	1.5037791565	0.2238292899
C	-4.8484847655	2.5238576202	-3.7838385657
H	-3.4821245972	1.0648152191	-4.5898404766
H	-6.020393308	3.9919882826	-2.7237128778
C	-4.1925038151	0.1370559147	3.2590352528
H	-2.276934139	0.2061478408	4.248346632
H	-5.9570313916	0.2207819854	2.025279143
H	-5.4959084476	2.5199272649	-4.6556318877
H	-4.6720677448	-0.4341502734	4.0481471799
C	-1.9712944651	4.2774570937	0.4629662004
C	-1.192651952	4.4200612767	1.7786238933
C	-1.3256474571	5.1218738453	-0.6477378304
H	-3.0007307302	4.6237322261	0.6318874585
C	-1.0686973209	5.8919725715	2.1865380011
H	-0.1859941987	3.9931356496	1.6545877316
H	-1.6877983543	3.8580057553	2.5769030426
C	-1.2027995393	6.5896326231	-0.2286252648
H	-0.3228854837	4.7217192176	-0.8613596599
H	-1.9056092874	5.042814797	-1.5740559144
C	-0.4250848974	6.7291077963	1.0805678437
H	-0.4883468967	5.9673664023	3.1126688403
H	-2.0705792211	6.2877749685	2.4035232061
H	-0.7167403453	7.160266353	-1.027545198
H	-2.2092615603	7.0117188665	-0.1000032752
H	-0.3727780192	7.7810287347	1.3821005649
H	0.6082732615	6.3885789204	0.9243134206

TS1a-C3-ti
E(RM062X) = -1761.48852090
Sum of electronic and thermal Free Energies = -1761.014755
Charge = 0 Multiplicity = 1 NIMAG = 1

C	-0.2698674879	0.0616652822	0.2188299979
N	-0.0819423988	0.043403273	1.6685422357
C	1.1666267912	0.2762675455	2.1699659954
O	1.4238699151	0.1938736887	3.3724659698
C	2.3078373764	0.6406365057	1.2350776491
C	3.2959653138	-0.3107720934	0.9633933865
C	2.3799509667	1.9078203562	0.644717146
C	4.3302105811	-0.0225362923	0.0788140441
H	3.2524386743	-1.2880227147	1.4362954567
C	3.4114644973	2.2173779918	-0.2331593829
H	1.6159942217	2.6497291349	0.8597303566
C	4.360791024	1.2374474691	-0.5065104163
H	5.0911102525	-0.7566774518	-0.1589023318
H	3.4747700393	3.1891875019	-0.7083465574
N	5.4165439666	1.5317684926	-1.4805787427
O	5.3509596297	2.5839584448	-2.097604346
O	6.3012516928	0.7055092807	-1.6376365547
C	-1.6719662933	0.4397440211	-0.1987294823
H	-1.8449329836	1.5026622185	-0.0059174293
H	-1.8181325857	0.2521625598	-1.2677699127
H	-2.4088280973	-0.1389360869	0.3670877051
H	0.4883571382	0.5933339423	-0.3587077359
H	1.0433871727	-1.6936838841	0.5179950715
H	-0.7675539607	-2.0162083329	0.7739853985
C	0.0167275569	-1.3640561506	0.4057363269
P	0.2001167529	-2.4165917053	-1.8321886382
C	1.6398005498	-1.5489923681	-2.5683545511
C	-1.062987989	-2.3242115706	-3.1557926742
C	2.8766311662	-2.1585757324	-2.8133222087
C	1.4906956869	-0.1839137972	-2.8647313206
C	-2.4108233558	-2.2817686115	-2.7793338512
C	-0.7290570115	-2.3400142847	-4.5163820235
C	3.9358788941	-1.4216970461	-3.3481792044
H	3.0285177134	-3.2127039307	-2.6021043608
C	2.5450048115	0.5472552053	-3.4067170628
H	0.5348669471	0.3072874875	-2.6914170449
C	-3.4139472926	-2.2648414102	-3.7487762227
H	-2.6776644473	-2.2628950848	-1.7246840486
C	-1.7324089659	-2.3191508694	-5.483530888
H	0.3143815454	-2.368090215	-4.8212822633
C	3.7723866792	-0.0719763491	-3.6514837836
H	4.8887525532	-1.9092661082	-3.5323363547
H	2.4087564248	1.6002039307	-3.6367370878
C	-3.0747193077	-2.2821811133	-5.1010597986
H	-4.4564751293	-2.2318591114	-3.4471538739
H	-1.4661589321	-2.3312935002	-6.5362557787
H	4.5962212257	0.4963354622	-4.0745595687
H	-3.853805992	-2.2633554229	-5.8572313217
C	0.6877942141	-4.2088908212	-1.7650081539
C	1.5706580003	-4.4578220118	-0.5286123578
C	-0.5500118681	-5.117641375	-1.7201975389
H	1.2513037854	-4.4423128198	-2.6806589745

C	1.9956531206	-5.9264069098	-0.4475915976
H	0.9907396876	-4.2046652985	0.3707359513
H	2.4513024028	-3.8044033641	-0.5304926262
C	-0.1380460259	-6.5899746826	-1.6177565921
H	-1.1684229305	-4.8475694192	-0.8506880293
H	-1.1660778146	-4.966772393	-2.6126357575
C	0.7728979708	-6.8450795628	-0.4154780353
H	2.6157622609	-6.082916561	0.4417299124
H	2.6166320736	-6.1722713443	-1.3203854939
H	-1.0342664979	-7.2171504003	-1.5579269164
H	0.3901412024	-6.874631527	-2.5383403974
H	1.0856218599	-7.8947407746	-0.3941596405
H	0.2104572357	-6.6594146738	0.510266058

TS1a-C3-ci
E(RM062X) = -1761.47896717
Sum of electronic and thermal Free Energies = -1761.006883
Charge = 0 Multiplicity = 1 NIMAG = 1

C	-0.0002627987	-0.0275396261	0.0648643864
N	0.0564741784	0.0910132787	1.506981016
C	1.1868863365	0.1441396509	2.2346015369
O	1.3391609269	0.8509059911	3.2408184889
C	2.3056312534	-0.8316570705	1.9053993163
C	3.6342929976	-0.4333625572	2.0783274286
C	2.019017358	-2.1363579464	1.4919108384
C	4.6762734183	-1.3125284272	1.8079932183
H	3.8456134337	0.5734434949	2.4237338964
C	3.0455036566	-3.0399175279	1.2409937223
H	0.9853796246	-2.4467440569	1.3696866401
C	4.3568739284	-2.6024076005	1.3942684247
H	5.711804457	-1.0139618152	1.9202255858
H	2.8394696176	-4.0569676453	0.9293800818
N	5.4494297451	-3.5424445543	1.1125750261
O	5.1569612358	-4.6791308855	0.7757652502
O	6.5967403791	-3.1406992331	1.2269406273
H	-0.6597853394	-0.837122334	-0.2664673898
C	1.2513317481	0.0566646704	-0.7936983978
H	1.7926148403	-0.8908282801	-0.8313934664
H	0.9687605695	0.32012779	-1.8192823654
H	1.9309573854	0.8272942059	-0.4104896159
H	-0.1109690204	2.1513322061	0.4323790575
H	-1.7214081242	1.2569819967	0.6511940614
C	-0.7145650988	1.2664623281	0.2536226333
P	-1.4819304652	2.1103871704	-1.8291577469
C	-1.4526093933	0.991725668	-3.2806397683
C	-0.5463082242	3.5852853997	-2.3720054503
C	-1.7972005858	-0.353951408	-3.0848240929
C	-1.0739491775	1.41852358	-4.55941501
C	0.8202382656	3.6354936957	-2.0683314091
C	-1.1259102042	4.6452652389	-3.082949012
C	-1.7839156138	-1.2502601515	-4.1512786445
H	-2.0751935318	-0.7043923262	-2.093645972
C	-1.0542866822	0.5170526861	-5.6242393301
H	-0.7886363766	2.4527804393	-4.7297713154
C	1.5982463311	4.7179358586	-2.4787466277
H	1.2815339855	2.8246853014	-1.5098385703
C	-0.3491755828	5.7303002007	-3.4848554865

H	-2.1835144857	4.6305643715	-3.3316169996
C	-1.4106384137	-0.8157993378	-5.4237204394
H	-2.0559561891	-2.2883632786	-3.9858081357
H	-0.7571457706	0.8594065703	-6.610996383
C	1.0137154426	5.7671132149	-3.1861086612
H	2.6569531224	4.7423248629	-2.2392794124
H	-0.8091020092	6.5460828109	-4.0344744071
H	-1.3915371215	-1.5155131367	-6.2538158649
H	1.6164369114	6.6134985544	-3.5014423048
C	-3.2450925262	2.6685505598	-1.6657093627
C	-4.1636683892	1.4728535836	-1.3678107613
C	-3.3454390255	3.7245245927	-0.5519398668
H	-3.5575800476	3.1112699763	-2.6223322562
C	-5.6096374935	1.933379892	-1.1557918921
H	-3.8101044721	0.9583822437	-0.4617425386
H	-4.1244894973	0.7495823288	-2.1887502438
C	-4.7951417402	4.1761101139	-0.3520619491
H	-2.9722907758	3.2908511597	0.3873222526
H	-2.7105168457	4.5873109468	-0.7803066153
C	-5.7084394206	2.9869206693	-0.0519674066
H	-6.2371491838	1.0675478295	-0.9180870189
H	-5.990180649	2.3568080224	-2.0956795443
H	-4.8411245879	4.9107006211	0.4591804226
H	-5.1433788377	4.6816303572	-1.263690082
H	-6.7453115634	3.322018942	0.0605715089
H	-5.4108066585	2.5369609141	0.905597667

TS1a-C3-co
E(RM062X) = -1761.48936588
Sum of electronic and thermal Free Energies = -1761.018937
Charge = 0 Multiplicity = 1 NIMAG = 1

C	-0.0298563861	-0.09385221	-0.0262430578
H	-0.0376305398	-0.1728929289	1.0625475794
N	1.374051521	-0.1289681902	-0.4612492639
C	1.6503070998	-0.419672897	-1.7624364162
O	0.8586110807	-0.5391905661	-2.7057002807
C	3.1334221436	-0.5849249476	-2.0398503559
C	3.5303487756	-0.8983331213	-3.3440706511
C	4.0996109432	-0.4340940764	-1.0387418747
C	4.8744672965	-1.0596942344	-3.6575774537
H	2.7701699176	-1.0125658746	-4.1089386365
C	5.449876054	-0.5866390144	-1.3329403938
H	3.7851529291	-0.1967864261	-0.0294187397
C	5.8089957452	-0.8976796163	-2.6401636392
H	5.1952613122	-1.3029891826	-4.6634201073
H	6.2095561892	-0.4708764169	-0.5692015946
N	7.233688349	-1.0614258781	-2.9590286182
O	8.045228031	-0.9015570854	-2.0608995462
O	7.5359614099	-1.3485310644	-4.1065290146
C	-1.0930813324	-0.9930337113	-0.6254324455
H	-0.828623782	-2.0400655316	-0.4448348681
H	-2.0459435444	-0.7946701448	-0.1211142731
H	-1.2083608743	-0.8484645288	-1.697599596
H	0.6521552296	2.0031547557	0.1543414083
H	0.0729528773	1.4972040432	-1.5437982291
C	0.1460093383	1.2885221463	-0.4821520456
P	-2.0349444305	2.501532081	-0.0991213153

C	-3.1786820161	2.537896994	-1.5310163335
C	-2.9547509273	1.6087926965	1.2131107344
C	-2.887246028	1.7177597688	-2.6275726106
C	-4.3144944511	3.3598988872	-1.5792185544
C	-2.2331372079	1.219396067	2.3527169591
C	-4.3002003696	1.2355853599	1.1054240072
C	-3.7207410158	1.7069018973	-3.7474374216
H	-2.0027645244	1.0863555406	-2.6131197188
C	-5.1431586249	3.3523511106	-2.698699389
H	-4.5614026591	4.0055655625	-0.7402696019
C	-2.8490237289	0.4968525836	3.372364258
H	-1.1793930498	1.475235892	2.4409249053
C	-4.9134780442	0.5049255861	2.1238952495
H	-4.8746832242	1.5037791565	0.2238292899
C	-4.8484847655	2.5238576202	-3.7838385657
H	-3.4821245972	1.0648152191	-4.5898404766
H	-6.020393308	3.9919882826	-2.7237128778
C	-4.1925038151	0.1370559147	3.2590352528
H	-2.276934139	0.2061478408	4.248346632
H	-5.9570313916	0.2207819854	2.025279143
H	-5.4959084476	2.5199272649	-4.6556318877
H	-4.6720677448	-0.4341502734	4.0481471799
C	-1.9712944651	4.2774570937	0.4629662004
C	-1.192651952	4.4200612767	1.7786238933
C	-1.3256474571	5.1218738453	-0.6477378304
H	-3.0007307302	4.6237322261	0.6318874585
C	-1.0686973209	5.8919725715	2.1865380011
H	-0.1859941987	3.9931356496	1.6545877316
H	-1.6877983543	3.8580057553	2.5769030426
C	-1.2027995393	6.5896326231	-0.2286252648
H	-0.3228854837	4.7217192176	-0.8613596599
H	-1.9056092874	5.042814797	-1.5740559144
C	-0.4250848974	6.7291077963	1.0805678437
H	-0.4883468967	5.9673664023	3.1126688403
H	-2.0705792211	6.2877749685	2.4035232061
H	-0.7167403453	7.160266353	-1.027545198
H	-2.2092615603	7.0117188665	-0.1000032752
H	-0.3727780192	7.7810287347	1.3821005649
H	0.6082732615	6.3885789204	0.9243134206

TS1a-C3-ti
E(RM062X) = -1761.48852090
Sum of electronic and thermal Free Energies = -1761.014755
Charge = 0 Multiplicity = 1 NIMAG = 1

C	-0.2698674879	0.0616652822	0.2188299979
N	-0.0819423988	0.043403273	1.6685422357
C	1.1666267912	0.2762675455	2.1699659954
O	1.4238699151	0.1938736887	3.3724659698
C	2.3078373764	0.6406365057	1.2350776491
C	3.2959653138	-0.3107720934	0.9633933865
C	2.3799509667	1.9078203562	0.644717146
C	4.3302105811	-0.0225362923	0.0788140441
H	3.2524386743	-1.2880227147	1.4362954567
C	3.4114644973	2.2173779918	-0.2331593829
H	1.6159942217	2.6497291349	0.8597303566
C	4.360791024	1.2374474691	-0.5065104163
H	5.0911102525	-0.7566774518	-0.1589023318

H	3.4747700393	3.1891875019	-0.7083465574
N	5.4165439666	1.5317684926	-1.4805787427
O	5.3509596297	2.5839584448	-2.097604346
O	6.3012516928	0.7055092807	-1.6376365547
C	-1.6719662933	0.4397440211	-0.1987294823
H	-1.8449329836	1.5026622185	-0.0059174293
H	-1.8181325857	0.2521625598	-1.2677699127
H	-2.4088280973	-0.1389360869	0.3670877051
H	0.4883571382	0.5933339423	-0.3587077359
H	1.0433871727	-1.6936838841	0.5179950715
H	-0.7675539607	-2.0162083329	0.7739853985
C	0.0167275569	-1.3640561506	0.4057363269
P	0.2001167529	-2.4165917053	-1.8321886382
C	1.6398005498	-1.5489923681	-2.5683545511
C	-1.062987989	-2.3242115706	-3.1557926742
C	2.8766311662	-2.1585757324	-2.8133222087
C	1.4906956869	-0.1839137972	-2.8647313206
C	-2.4108233558	-2.2817686115	-2.7793338512
C	-0.7290570115	-2.3400142847	-4.5163820235
C	3.9358788941	-1.4216970461	-3.3481792044
H	3.0285177134	-3.2127039307	-2.6021043608
C	2.5450048115	0.5472552053	-3.4067170628
H	0.5348669471	0.3072874875	-2.6914170449
C	-3.4139472926	-2.2648414102	-3.7487762227
H	-2.6776644473	-2.2628950848	-1.7246840486
C	-1.7324089659	-2.3191508694	-5.483530888
H	0.3143815454	-2.368090215	-4.8212822633
C	3.7723866792	-0.0719763491	-3.6514837836
H	4.8887525532	-1.9092661082	-3.5323363547
H	2.4087564248	1.6002039307	-3.6367370878
C	-3.0747193077	-2.2821811133	-5.1010597986
H	-4.4564751293	-2.2318591114	-3.4471538739
H	-1.4661589321	-2.3312935002	-6.5362557787
H	4.5962212257	0.4963354622	-4.0745595687
H	-3.853805992	-2.2633554229	-5.8572313217
C	0.6877942141	-4.2088908212	-1.7650081539
C	1.5706580003	-4.4578220118	-0.5286123578
C	-0.5500118681	-5.117641375	-1.7201975389
H	1.2513037854	-4.4423128198	-2.6806589745
C	1.9956531206	-5.9264069098	-0.4475915976
H	0.9907396876	-4.2046652985	0.3707359513
H	2.4513024028	-3.8044033641	-0.5304926262
C	-0.1380460259	-6.5899746826	-1.6177565921
H	-1.1684229305	-4.8475694192	-0.8506880293
H	-1.1660778146	-4.966772393	-2.6126357575
C	0.7728979708	-6.8450795628	-0.4154780353
H	2.6157622609	-6.082916561	0.4417299124
H	2.6166320736	-6.1722713443	-1.3203854939
H	-1.0342664979	-7.2171504003	-1.5579269164
H	0.3901412024	-6.874631527	-2.5383403974
H	1.0856218599	-7.8947407746	-0.3941596405
H	0.2104572357	-6.6594146738	0.510266058

TS1cC2-to_P(Me)3
E(RM062X) = -1182.85771319
Sum of electronic and thermal Free Energies = -1182.605713
Charge = 0 Multiplicity = 1 NIMAG = 1

C	3.3978219074	-1.3966039316	-1.1131590011
C	3.8041260515	-0.1755642547	-0.4100591707
H	4.0462631826	0.6803925387	-1.0416994569
H	4.5148880895	-0.3283729915	0.4040068813
N	2.4230035681	-0.2526331918	0.0481787813
C	1.556611334	0.4743294467	-0.7082280979
O	1.8368282446	1.1675934789	-1.6951686869
C	0.1154511851	0.388046452	-0.2553067424
C	-0.8383381103	1.1649347532	-0.920758905
C	-0.2791705911	-0.4464812205	0.7966886798
C	-2.1760063918	1.1176229917	-0.5474490454
H	-0.5167984535	1.8056224715	-1.7345712542
C	-1.613656933	-0.5125998456	1.1807544396
H	0.4657343444	-1.0409783867	1.3127626171
C	-2.5356474289	0.2748301213	0.4990366449
H	-2.925300825	1.7155695964	-1.0519853302
H	-1.9357908484	-1.1556833292	1.9909357011
N	-3.9474786407	0.214236558	0.9012878092
O	-4.2516393339	-0.5319663803	1.8185475355
O	-4.7466382236	0.9128735755	0.2981439068
H	2.8452885267	-1.2533940886	-2.0356326339
C	3.3660574954	-2.748124086	-0.4501063973
H	2.3990265194	-2.8965841314	0.037270465
H	3.4934523825	-3.5428892933	-1.1880684439
H	4.15125005	-2.8326685599	0.3067974506
P	5.5482552441	-1.8338762062	-2.4616536463
C	5.7593471334	-2.6924712498	-4.0752773837
C	6.7324685324	-2.746050232	-1.3912308068
C	6.4418724385	-0.2535307753	-2.7457761612
H	5.3573938641	-3.7074577577	-4.0051587987
H	5.2057179997	-2.1544488231	-4.8499796426
H	6.8167417753	-2.7429350147	-4.3574460254
H	6.7976265942	-2.2570109739	-0.4142786751
H	6.3824072026	-3.7719256024	-1.2417932184
H	7.7306108505	-2.7725436413	-1.8420741805
H	5.8643989301	0.3791449854	-3.4262880295
H	6.5587327048	0.2775050612	-1.796276049
H	7.4329707193	-0.4336263531	-3.1762668395

TS1cC2-ti_P(Me)3
E(RM062X) = -1182.84374146
Sum of electronic and thermal Free Energies = -1182.590992
Charge = 0 Multiplicity = 1 NIMAG = 1

C	-4.2992038474	0.4658006461	0.291550704
C	-3.0729695379	1.2759696968	0.1408421798
H	-4.1674446936	-0.51996539	0.7250517179
H	-2.3450246089	1.1576490241	0.9499268951
H	-3.2453162082	2.3312918102	-0.0930602581
N	-2.934199713	0.4320667368	-1.0224781407
C	-1.9936756533	-0.5404655089	-1.0602453209
O	-2.120519068	-1.5899766281	-1.7018369757
C	-0.6603134409	-0.3128779747	-0.3641797664
C	-0.0074527732	-1.404511726	0.2161462212
C	-0.049525953	0.9461404004	-0.3541356808
C	1.2320522382	-1.2465719839	0.8255881844
H	-0.4767168759	-2.3822173842	0.1809351125
C	1.1988338961	1.1215333019	0.2318098027

H	-0.5468847249	1.7919925841	-0.8199884487
C	1.8101802555	0.0185654326	0.818730088
H	1.7446681154	-2.0812564114	1.2886942957
H	1.6901049991	2.0871517811	0.2357698888
N	3.1227810016	0.1974820399	1.4534637423
O	3.6189216045	1.313002817	1.4412834067
O	3.6513296084	-0.7774997435	1.9635256197
C	-5.5594514466	0.7391324125	-0.4936175364
H	-5.5468459599	0.172776893	-1.4264973296
H	-6.443938186	0.4358898724	0.0719883085
H	-5.6397653441	1.8035593865	-0.7347637323
P	-5.1498619219	1.364019848	2.4603434669
C	-6.0653040554	2.9312211943	2.1846043807
C	-3.8259152865	1.8827275244	3.6214160313
C	-6.2964557577	0.4274853621	3.5475082107
H	-5.4423033137	3.6262797004	1.6135812059
H	-6.9756745159	2.7308844887	1.6118446755
H	-6.3392936271	3.3974954921	3.137222919
H	-3.2311673163	1.0127331069	3.9148936348
H	-3.1671398451	2.6028993725	3.127064554
H	-4.2501844682	2.3465359112	4.5185223747
H	-7.1892883456	0.146010869	2.9813766903
H	-5.8060247555	-0.4855051144	3.8968322121
H	-6.5951059352	1.0299027102	4.4122919366

TS1cC2-co_P(Me)3
E(RM062X) = -1182.85629715
Sum of electronic and thermal Free Energies = -1182.603997
Charge = 0 Multiplicity = 1 NIMAG = 1

C	3.7937763948	-0.8227259643	-0.0211648054
C	3.2528663721	-1.7798774613	0.9539786732
N	2.3887011005	-0.7422294571	-0.3797503413
C	1.7357267953	0.3505348177	0.0942671995
O	2.2317840278	1.3132991687	0.6931115619
C	0.2445660011	0.3405166793	-0.1648412713
C	-0.5105003144	1.4450912726	0.243207666
C	-0.3888439236	-0.7436213516	-0.7829951589
C	-1.8847054915	1.4784821313	0.0392102242
H	-0.0054947559	2.2763178162	0.7230284593
C	-1.7630646953	-0.7311590052	-0.9925558579
H	0.2035827696	-1.5942581215	-1.0992477677
C	-2.4830837233	0.3840692194	-0.5767576768
H	-2.4814080352	2.3281082296	0.3490413901
H	-2.2680251395	-1.563308676	-1.46825226
N	-3.9357053582	0.4064911404	-0.7971237835
O	-4.4497064096	-0.5603133683	-1.3372776386
O	-4.5569926554	1.3908103352	-0.4290873217
H	4.4042107583	-1.2580073443	-0.813634236
H	4.2313865279	0.0826842892	0.4056160238
H	2.9893641769	-2.7609541161	0.5775433156
C	2.7245514879	-1.3783314176	2.3090804585
H	3.2671113483	-0.5208338017	2.7128391622
H	1.6627614374	-1.1196744351	2.2508826495
H	2.8108342686	-2.2181812	3.0015508136
P	5.4872997777	-2.6973334022	1.8495098388
C	5.8482859477	-4.3679530655	2.5300669572
C	6.0737512386	-1.6015161809	3.2031688668

C	6.8201239557	-2.4924785399	0.6005895815
H	5.1777958435	-4.5734706627	3.369684974
H	5.6757763332	-5.1225635512	1.7573622419
H	6.886129041	-4.4358895566	2.8742659998
H	6.0181156524	-0.5570967484	2.8810780146
H	5.4366850076	-1.7263321645	4.0838886081
H	7.1086798081	-1.834784771	3.4763664636
H	6.6338558491	-3.1523291644	-0.2518345052
H	6.8313817692	-1.4584487297	0.2430551293
H	7.7995271117	-2.7297769627	1.0302646514

TS1cC2-ci_P(Me)3
E(RM062X) = -1182.84596665
Sum of electronic and thermal Free Energies = -1182.593573
Charge = 0 Multiplicity = 1 NIMAG = 1

C	-1.2727772563	0.5410396851	-1.0632671918
C	-2.2997293891	1.1196806386	-0.1705033702
H	-3.0830636163	1.6991032187	-0.6434596087
N	-0.8016502986	1.904705351	-1.0252946995
C	0.2974923196	2.2386673571	-0.3112465628
O	0.4472758646	3.3457003679	0.2200196392
C	1.4476192676	1.2521954688	-0.2010687049
C	2.1661425156	1.1855162376	0.9963578307
C	1.831054519	0.4579269388	-1.2871297062
C	3.246280537	0.3199738389	1.1258128432
H	1.8734937127	1.8206706792	1.8262985727
C	2.9220899875	-0.3975481536	-1.1856942958
H	1.2809411396	0.5179603434	-2.2218252787
C	3.6026094429	-0.4537155507	0.0261372344
H	3.8061124247	0.2488268447	2.0506531507
H	3.2403309357	-1.0084584271	-2.0219303676
N	4.7471662029	-1.3665088654	0.1478883709
O	5.0574592067	-2.0309727323	-0.8282129139
O	5.3297179154	-1.4167486009	1.2194606478
H	-1.633722113	0.2227727726	-2.0445407627
H	-0.6518147716	-0.2301128683	-0.5929178352
C	-2.0497124849	1.3266242285	1.3028835857
H	-1.639065357	2.3260216775	1.4791604128
H	-2.9808642764	1.2467130852	1.8686402408
H	-1.3376853672	0.5871697691	1.685464294
P	-3.8242485625	-0.8472848414	0.0620446635
C	-3.2398856008	-1.947785965	1.4108068472
C	-3.7832809323	-1.9656242059	-1.3937208529
C	-5.6222290057	-0.7168562686	0.4155148995
H	-2.1833844082	-2.1885733604	1.2577909188
H	-3.3439222183	-1.4404121378	2.374465675
H	-3.8173771951	-2.8783525346	1.433896462
H	-4.1785084954	-1.4446515951	-2.2706242442
H	-2.7499345026	-2.2593037106	-1.6008894304
H	-4.3795533971	-2.8664874482	-1.2123450408
H	-5.772965311	-0.1492023103	1.3384065911
H	-6.1178856343	-0.1858878661	-0.4020406459
H	-6.0725967976	-1.7092540604	0.5266506324

TS1cC3-ti_P(Me)3
E(RM062X) = -1182.84934362
Sum of electronic and thermal Free Energies = -1182.595704

Charge = 0 Multiplicity = 1 NIMAG = 1

C	2.9114459542	-1.254799961	-0.6621208857
N	2.922973255	-1.0161676385	0.7663632373
C	2.215985063	0.0055867581	1.3100287173
O	2.5626330281	0.6066319196	2.3320429678
C	0.8792676292	0.3912549251	0.6976082067
C	0.4952916258	1.73558207	0.7123440092
C	0.0076809767	-0.5740389938	0.1813392617
C	-0.7347699746	2.1262278746	0.1956486841
H	1.167257803	2.474070583	1.1371162613
C	-1.235261036	-0.2069436579	-0.321601309
H	0.2963505067	-1.6209863076	0.1878890476
C	-1.5763446461	1.1414083142	-0.3109892522
H	-1.0411776837	3.1654730476	0.1902651216
H	-1.9264539372	-0.9441303234	-0.7122095676
N	-2.8822224464	1.5403171742	-0.8530164118
O	-3.6102792373	0.6666440035	-1.2970611819
O	-3.1734814153	2.7255794137	-0.8343316448
C	2.8212447569	-2.708996134	-1.0682854632
H	1.8129553356	-3.0933359045	-0.8860883416
H	3.0472658371	-2.8342022023	-2.1324429452
H	3.5281086897	-3.3063757013	-0.4832490102
H	2.2531901331	-0.6089012428	-1.2541630044
H	4.3866287233	0.3834266865	-0.3914104907
H	5.0462503072	-1.324193967	-0.0714696036
C	4.2652743918	-0.6900859411	-0.4714883939
P	5.2877589731	-0.7082153878	-2.6979131195
C	6.0684658523	-2.2882670251	-3.2095994334
H	6.9475114562	-2.4768960914	-2.5864818102
H	5.3632381856	-3.1125297716	-3.0695023932
H	6.3740665297	-2.251884054	-4.2608375277
C	3.9974854929	-0.4602951448	-3.9789052129
H	3.5248569263	0.5161842194	-3.8363738445
H	4.4301332165	-0.5054783441	-4.9841599388
H	3.2294328012	-1.2338759363	-3.8866907111
C	6.5553729886	0.5327426174	-3.1718667069
H	6.839275325	0.4189235358	-4.2236655423
H	6.15820791	1.5395733031	-3.014041008
H	7.442208363	0.4078627247	-2.5442386701

TS1cC3-to_P(Me)3
E(RM062X) = -1182.86183824
Sum of electronic and thermal Free Energies = -1182.610234
Charge = 0 Multiplicity = 1 NIMAG = 1

C	-3.3181866902	0.712462621	0.7758189072
C	-2.6081806567	1.7636997432	1.5098519307
H	-3.5880693458	-0.155439072	1.3803526447
H	-1.9898900717	1.4964219984	2.3604713005
H	-2.4705303292	2.7324740497	1.0476451062
C	-4.3923493509	1.148189831	-0.1917572411
H	-4.6225333264	0.3406387742	-0.8926556096
H	-5.3099670653	1.4086648654	0.3472878229
H	-4.0613226231	2.0210332523	-0.7630618099
N	-1.9835058685	0.637606311	0.1864295993
C	-1.1645071151	-0.2912612432	0.7601514372
O	-1.4454633528	-1.0373370471	1.7053610132

C	0.214066584	-0.3706373548	0.1430359909
C	1.1132455728	-1.3169453262	0.6452724727
C	0.6047253864	0.4744366762	-0.9016305122
C	2.3943214859	-1.4260557815	0.1181366056
H	0.7951533334	-1.9647161345	1.4547734422
C	1.8835341555	0.3837174483	-1.4393044996
H	-0.0992050005	1.2014823157	-1.2894055584
C	2.7528776382	-0.5676351727	-0.915856471
H	3.1013057041	-2.1546059004	0.4965394242
H	2.2025749121	1.0318237422	-2.2467062409
N	4.1069866375	-0.6683628299	-1.477648204
O	4.4150840097	0.1010004978	-2.3740865706
O	4.8571171079	-1.515391092	-1.0191705997
P	-4.2842423959	2.6333636632	3.1683545774
C	-5.145067726	1.094005363	3.6787478655
H	-5.819179265	1.280969455	4.521593883
H	-5.7258155863	0.6968152929	2.8405101164
H	-4.4051961303	0.343108898	3.973482674
C	-3.5970883724	3.227394312	4.7659031069
H	-3.1463554841	4.2142722308	4.6277321634
H	-4.378293263	3.2920882532	5.5310655161
H	-2.8198258244	2.5368734229	5.1067245066
C	-5.6875463952	3.78964336	2.893922542
H	-6.347460448	3.8165326168	3.7680249464
H	-5.3015728339	4.7950443894	2.7036317025
H	-6.2629493068	3.4695115105	2.0205336496

TS1cC3-ci_P(Me)3
E(RM062X) = -1182.84445226
Sum of electronic and thermal Free Energies = -1182.591788
Charge = 0 Multiplicity = 1 NIMAG = 1

C	3.0190946752	-1.1792345291	-0.0182310312
N	2.8697122149	-0.0754425312	-0.9450549371
C	1.9677959965	0.9175357605	-0.8236417278
O	2.1947724473	2.1039637169	-1.0959066482
C	0.5343240416	0.5525672387	-0.4732487588
C	-0.2254494629	1.4355147092	0.2994894822
C	-0.0478637591	-0.6204822233	-0.9636021075
C	-1.5472565792	1.1421006566	0.6133885756
H	0.2283511437	2.354737346	0.6553165081
C	-1.3759727277	-0.9209733332	-0.6822341041
H	0.5409298916	-1.2987686997	-1.5741913394
C	-2.0957225517	-0.0343471361	0.1117442256
H	-2.1449122272	1.807729468	1.2247139924
H	-1.8448116953	-1.8201020241	-1.0637691195
N	-3.494920484	-0.3497372058	0.4280033466
O	-3.9720444079	-1.3735715036	-0.0358224132
O	-4.1115035916	0.426555811	1.1405741891
H	3.0547752286	-2.1530034221	-0.5197157719
C	2.2407858574	-1.2470690283	1.2848422865
H	1.2199830971	-1.6082466599	1.1449972677
H	2.7468738411	-1.9388083611	1.9677286459
H	2.1990225031	-0.2614700803	1.7632909752
H	4.4250246797	0.3550942777	0.7233494597
H	5.073149061	-0.6404055214	-0.704521826
C	4.347565888	-0.5215896206	0.0903556559
P	5.689753018	-1.8982977611	1.5112472719

C	5.6318521962	-1.8628452558	3.3443123785
H	4.6162845873	-2.0803048139	3.6867858939
H	6.3203711602	-2.6018092697	3.7682398023
H	5.9113067032	-0.8664529573	3.6980480716
C	5.3717223169	-3.6605199565	1.1188271151
H	5.4731138031	-3.8160346289	0.0404953403
H	6.0796934966	-4.3121348857	1.6420353808
H	4.3534084908	-3.9297474741	1.4147626681
C	7.4776096648	-1.7188194386	1.1507902315
H	8.0645163402	-2.4699659838	1.6898414293
H	7.6455538939	-1.8361411123	0.0761319014
H	7.8116469486	-0.7202704072	1.4467417395

TS1cC3-co_P(Me)3
E(RM062X) = -1182.85405092
Sum of electronic and thermal Free Energies = -1182.600242
Charge = 0 Multiplicity = 1 NIMAG = 1

C	-3.2896753576	-1.2845424845	0.3898120916
H	-3.7769590239	-1.7955412211	-0.4435407482
N	-1.8991875576	-1.0204729023	-0.001770056
C	-1.2020013146	-0.0544024475	0.6609103396
O	-1.5555833055	0.597860304	1.6491690301
C	0.1783027485	0.1968520427	0.08310788
C	0.9829990316	1.1647680988	0.6934346218
C	0.6591444169	-0.5035571774	-1.0288967144
C	2.2562113841	1.4376602636	0.2084001538
H	0.596322559	1.7000316672	1.5536548117
C	1.93151393	-0.2474469928	-1.5276356075
H	0.0296910161	-1.2492607101	-1.4996703191
C	2.7043727662	0.7215882771	-0.896673796
H	2.8895213925	2.1848650878	0.671398249
H	2.3182812693	-0.7829910997	-2.3863572609
N	4.0492714989	1.0002057846	-1.4186907843
O	4.4257103982	0.3757804227	-2.3979957171
O	4.7239831261	1.8420256361	-0.8471864427
C	-4.2021195244	-0.2283107855	0.9839227548
H	-4.2619935361	0.6374962415	0.3171579018
H	-5.2086864902	-0.6517308615	1.0732748398
H	-3.862048239	0.1125908418	1.960867051
H	-2.1952002623	-3.149402424	0.8851172785
H	-2.1401107942	-1.8095693012	2.1848267862
P	-4.3625559061	-3.4554720916	2.5131727404
C	-5.5936195747	-3.8164851724	1.1979201363
H	-6.4318135364	-4.401377941	1.5917493034
H	-5.9791535581	-2.880179428	0.7831351235
H	-5.1128561321	-4.3798173904	0.3920398506
C	-5.3974628302	-2.6410527517	3.7960218522
H	-6.2705068977	-3.2558076673	4.041445208
H	-4.8019634161	-2.4856373929	4.700194399
H	-5.7358768236	-1.665587165	3.4352047534
C	-4.0937237995	-5.1225790934	3.2418660147
H	-3.5882083637	-5.7627401098	2.5129633103
H	-3.4571205724	-5.0375927263	4.1270825991
H	-5.0456318021	-5.5844797763	3.5258645142
C	-2.5555970994	-2.2015646243	1.2640482316

TS1bC2-to

E(RM062X) = -2016.62822451
Sum of electronic and thermal Free Energies = -2016.168666
Charge = 0 Multiplicity = 1 NIMAG = 1

C	-0.0201190965	-0.0233727305	0.0271947924
C	-0.0466102918	-0.0153005577	1.4945496733
H	0.9260094663	-0.0287759499	1.9912839163
H	-0.7688772918	-0.7027526832	1.9387907166
N	-0.6167327347	1.3069688558	1.2693711297
C	0.3144241869	2.3009767679	1.2572047906
O	1.5375239413	2.1605556687	1.4106783176
C	-0.2481821677	3.680375381	1.0188558287
C	0.6232187004	4.7730386208	1.0342112946
C	-1.6087111258	3.8952191234	0.7757430473
C	0.1535625206	6.064421989	0.8116711153
H	1.6776541416	4.59906598	1.2225798661
C	-2.0951992768	5.1798282409	0.5465671019
H	-2.2881894659	3.0499888712	0.7674274117
C	-1.204441843	6.2490666964	0.5687448273
H	0.8288673454	6.9136675498	0.8251394646
H	-3.1499132099	5.3487045526	0.3555697343
H	0.7932592097	0.5270861951	-0.4352871966
C	-1.2263926971	-0.377251717	-0.8007597052
H	-1.871262852	0.499195501	-0.9045586923
H	-0.9271419615	-0.6954811535	-1.8022849202
H	-1.8033820546	-1.1783030241	-0.3288832171
P	1.2390242363	-2.1910854748	-0.3631205256
C	2.0149383411	-2.6786189343	-1.9516696224
C	0.1308372971	-3.5844194381	0.0519890634
C	2.3944294817	-1.6531790078	-2.8270381061
C	2.2650268935	-4.0106178463	-2.3121461283
C	-0.7408092007	-4.0763082291	-0.9329824923
C	0.0139271792	-4.0743368274	1.3598679949
C	3.0255152467	-1.9497357091	-4.0360884258
H	2.1964508051	-0.6162021569	-2.56424547
C	2.8876622599	-4.3058828743	-3.5228159331
H	1.973764891	-4.8188926724	-1.6461553589
C	-1.6880764006	-5.0484271436	-0.6215269137
H	-0.6757183553	-3.7003403889	-1.9516861008
C	-0.9382697903	-5.046244427	1.6701732708
H	0.6660205263	-3.7072433673	2.1476122995
C	3.2704386723	-3.2763125809	-4.385392045
H	3.3167314986	-1.1451156947	-4.7044115648
H	3.0772784825	-5.3407686491	-3.7919379614
C	-1.7892778147	-5.5376992243	0.6820070784
H	-2.3493223451	-5.4220876857	-1.3976995631
H	-1.0103465829	-5.4191696118	2.687534333
H	3.7555405139	-3.5100932121	-5.3284047022
H	-2.5280347281	-6.29533544	0.9248588606
C	2.6199114261	-2.241402438	0.8768629797
C	3.4566706954	-3.5252188712	0.8842351964
C	3.5125549286	-1.003994376	0.683230849
H	2.1175802205	-2.1341085	1.8502060115
C	4.5297323341	-3.4620726257	1.9768425738
H	3.9463546804	-3.6353304992	-0.0929538795
H	2.8179152174	-4.4049529484	1.025320166
C	4.5950429373	-0.9462770956	1.7659650019
H	3.9910379305	-1.0575233742	-0.3057514912

H	2.9091009324	-0.0876939006	0.7081270703
C	5.4237099269	-2.2322875919	1.8009134876
H	5.1315717141	-4.3775504619	1.9582403183
H	4.0412421864	-3.419379501	2.9605953724
H	5.2414851066	-0.0784336475	1.5953222048
H	4.1121155359	-0.797287396	2.7421194053
H	6.1650617479	-2.1833627862	2.6062031932
H	5.9805434067	-2.3268124603	0.8579516772
Cl	-1.8079708237	7.8681854993	0.2845996236

TS1bC3-to
E(RM062X) = -2016.63096574
Sum of electronic and thermal Free Energies = -2016.172479
Charge = 0 Multiplicity = 1 NIMAG = 1

C	0.0438817885	0.0145357795	0.0060369425
C	0.014323752	-0.0162012658	1.4734804438
H	1.049872627	0.0375281364	-0.4165043408
H	0.810401649	0.4740901003	2.0233335348
H	-0.92883858	-0.1990649868	1.9749042595
C	-0.9013354575	-0.9023252426	-0.733431425
H	-1.0101791049	-0.5696476477	-1.7699456555
H	-0.514121071	-1.9273598206	-0.7395414246
H	-1.8899411956	-0.8983497769	-0.2630762148
N	-0.5116877207	1.3377748965	0.2794605856
C	0.4169572883	2.3357036325	0.3463790931
O	1.6441973062	2.2037099465	0.2387768778
C	-0.1603204388	3.7105173222	0.5796781698
C	0.7108503279	4.8017199125	0.641584907
C	-1.5339065067	3.9236995579	0.7336858073
C	0.2281999528	6.0900433483	0.853916019
H	1.7753581511	4.6292756415	0.5206108264
C	-2.033914306	5.205198136	0.9506348873
H	-2.2124167613	3.0794244453	0.6816654992
C	-1.1430325903	6.2730560205	1.0068304748
H	0.903372742	6.9382085243	0.9000025765
H	-3.0990830683	5.3729709172	1.0717555892
P	0.8816132039	-2.2356477647	2.1935342493
C	1.8281407505	-2.9721428481	0.8103553762
C	2.1312778864	-2.1483266448	3.5325673199
C	2.7990395683	-2.1580417362	0.2066512276
C	1.6270071413	-4.2662587945	0.3168442177
C	2.0305234947	-1.110133752	4.4664724353
C	3.1518617501	-3.0982699549	3.669765975
C	3.5554835311	-2.6292984964	-0.8628553097
H	2.9696989574	-1.1498911017	0.5801686627
C	2.3809438263	-4.7345422196	-0.7612914011
H	0.8892552326	-4.9241171189	0.7664531403
C	2.9301679409	-1.0273663381	5.5298462645
H	1.2445414186	-0.3647422947	4.3654899795
C	4.0512335211	-3.0129925485	4.7307321241
H	3.2471642489	-3.9037071083	2.9452915118
C	3.3442161721	-3.919623971	-1.3526015869
H	4.3052295435	-1.9877512999	-1.3159145582
H	2.2148747112	-5.7409283731	-1.1340513105
C	3.9408436784	-1.9785310891	5.6620264817
H	2.8434472957	-0.2182691465	6.248787045
H	4.8398185388	-3.7530532297	4.8300381911

H	3.9287230471	-4.2867042494	-2.1908003143
H	4.6448992824	-1.9125144954	6.4861327044
C	-0.3004810352	-3.5512303719	2.7619840542
C	-0.812648723	-3.2418386579	4.1775979926
C	-1.4791538705	-3.6561642356	1.7799056944
H	0.244522589	-4.5065815176	2.794775784
C	-1.8267095936	-4.2962935123	4.6328201641
H	-1.2894743918	-2.2498644061	4.183014965
H	0.0226947411	-3.2008337261	4.8843537054
C	-2.4805710411	-4.7211503836	2.2359179464
H	-1.9862733868	-2.6812286043	1.735239053
H	-1.1284331436	-3.8752262093	0.7652906068
C	-2.9896705326	-4.4266188269	3.6477925689
H	-2.1974646721	-4.0405176789	5.6314521503
H	-1.3160279104	-5.2653964149	4.7199476309
H	-3.3158987923	-4.7684104269	1.5287972802
H	-1.9916428527	-5.7054007919	2.2216679833
H	-3.6781403709	-5.2128183295	3.9764606273
H	-3.5592041466	-3.4866829508	3.6346854438
Cl	-1.7633886892	7.8883657797	1.2762318885

TS1cC2-to
E(RM062X) = -1557.05746132
Sum of electronic and thermal Free Energies = -1556.585985
Charge = 0 Multiplicity = 1 NIMAG = 1

C	-0.025050379	-0.0332754696	0.0266288047
C	-0.0567326805	-0.0183677938	1.493930155
H	0.9144262089	-0.0342966673	1.993658332
H	-0.7820171296	-0.7030669402	1.9378063476
N	-0.6221569784	1.3051935622	1.2657707143
C	0.3138797159	2.2959343213	1.2502507147
O	1.5364795904	2.1448416416	1.4026900928
C	-0.2397904081	3.6792315346	1.0107659014
C	0.6382102195	4.7676810275	1.037893501
C	-1.5981219038	3.9011254788	0.7562045568
C	0.1679613028	6.0602364988	0.8153877328
H	1.6894479697	4.5824950442	1.2349419141
C	-2.0681500402	5.1936627387	0.5290468978
H	-2.278402769	3.0563046133	0.7391885421
C	-1.1872172816	6.2758296862	0.5594855483
H	0.8570572334	6.8994131	0.8404824354
H	-3.1233570325	5.3572288621	0.3303653838
H	0.788382792	0.5178807716	-0.4348414432
C	-1.2307302621	-0.3866030389	-0.8027300674
H	-1.8776660046	0.4888026311	-0.9023270412
H	-0.9312319012	-0.7000643824	-1.8057385371
H	-1.8058200093	-1.1909861277	-0.334097249
P	1.2323736797	-2.1952716184	-0.3581900807
C	2.0086175253	-2.6763524302	-1.9484584393
C	0.1303862038	-3.593677899	0.0558191271
C	2.3845878654	-1.6473704885	-2.8212198473
C	2.262604851	-4.0065925111	-2.3126454567
C	-0.7402520271	-4.0876024471	-0.9290237876
C	0.0169573847	-4.0861790286	1.3630376614
C	3.0159537935	-1.9387637914	-4.031387558
H	2.1837944705	-0.6116703169	-2.555548616
C	2.8854897602	-4.2966914037	-3.5244378565

H	1.9741788803	-4.8175670477	-1.6487065511
C	-1.6829093796	-5.0643778629	-0.6181828098
H	-0.6779937972	-3.7095525576	-1.9471285687
C	-0.9306645369	-5.0627208383	1.6727245599
H	0.6681021503	-3.7172584818	2.1507227331
C	3.2646933752	-3.2636442178	-4.3844290227
H	3.3044250499	-1.1314438439	-4.6976339913
H	3.0781058448	-5.3302796123	-3.7964113046
C	-1.7805350401	-5.5562915517	0.6846278541
H	-2.3434191501	-5.4395458261	-1.3942559282
H	-1.0001371316	-5.4375104725	2.6895785703
H	3.7500206278	-3.4934094007	-5.3283147711
H	-2.5156959448	-6.317573677	0.9269944368
C	2.6147711562	-2.2412680603	0.8801239249
C	3.4566834728	-3.5217396401	0.8845491628
C	3.5022573694	-0.9999723216	0.6874069421
H	2.1129846584	-2.1373998495	1.854113118
C	4.5304853002	-3.4561415472	1.9762999293
H	3.9459390859	-3.6281317862	-0.0932878628
H	2.8217238504	-4.4043755615	1.0246700054
C	4.585472053	-0.939785178	1.7693080269
H	3.9801520647	-1.0503486136	-0.3020414739
H	2.8955497004	-0.0859118576	0.714534572
C	5.4193607748	-2.2224779016	1.8016375038
H	5.1359404578	-4.3692006618	1.9556363783
H	4.0426941896	-3.4170401711	2.9605475645
H	5.2282225453	-0.0690434559	1.5994964604
H	4.1027347508	-0.7941727171	2.7460651399
H	6.1611486062	-2.171863668	2.6064363767
H	5.9758527419	-2.3133052739	0.8581014353
H	-1.5553396285	7.2827931093	0.3853443503

TS1cC3-to
E(RM062X) = -1557.06019108
Sum of electronic and thermal Free Energies = -1556.589029
Charge = 0 Multiplicity = 1 NIMAG = 1

C	0.0414480399	0.0128795861	0.0094280645
C	0.009968594	-0.0255140525	1.4767382055
H	1.0483904296	0.0375908778	-0.4109601019
H	0.8023148079	0.4680636599	2.0290238433
H	-0.9347467511	-0.2098207835	1.9748748587
C	-0.9008182222	-0.9034675822	-0.7348047918
H	-1.008387013	-0.5675833082	-1.77046111
H	-0.5123127187	-1.928072883	-0.7436911141
H	-1.8904368176	-0.90243986	-0.2664627737
N	-0.5161729728	1.3349382452	0.2829426178
C	0.4132876682	2.3326268841	0.3611839472
O	1.6416275364	2.1933283087	0.2691865615
C	-0.1618293282	3.7100587678	0.5871095773
C	0.7166386953	4.7931899642	0.6941734936
C	-1.5396527638	3.9328481867	0.6903875179
C	0.227997522	6.081275528	0.9025243935
H	1.7828648779	4.607635509	0.611175713
C	-2.0288083288	5.2212156322	0.9003137649
H	-2.2192803801	3.092097315	0.6023152509
C	-1.1470042927	6.2978439572	1.0065573366
H	0.9181274839	6.9160159138	0.9836183621

H	-3.0994955708	5.3860447639	0.9787614312
P	0.8807376852	-2.2381522096	2.1914268207
C	1.8260082103	-2.9738078405	0.8071406031
C	2.1321479733	-2.1482061968	3.5285346984
C	2.7959147259	-2.15915263	0.2025568691
C	1.6245295988	-4.267773278	0.3134256875
C	2.0301325262	-1.1104836113	4.4628549355
C	3.1558231314	-3.0951319644	3.6634715008
C	3.5508950703	-2.6297774316	-0.8682470427
H	2.9666889987	-1.1510762232	0.5761990263
C	2.3769703461	-4.7353619974	-0.7660471842
H	0.8874713867	-4.9259361319	0.7637295248
C	2.9316495978	-1.0253133974	5.5244378162
H	1.2418714796	-0.3672966399	4.3634542232
C	4.0570446048	-3.0074244878	4.7226725649
H	3.2521163454	-3.9000769268	2.9385898573
C	3.3392301997	-3.9199343916	-1.358293078
H	4.2997751274	-1.9878080608	-1.3221333252
H	2.2105542983	-5.7415897489	-1.139086119
C	3.9454499028	-1.9734624409	5.6543710779
H	2.8440323587	-0.2165239835	6.2436073821
H	4.8480559886	-3.7451288827	4.8202022415
H	3.922559289	-4.286432697	-2.1975679833
H	4.6510429665	-1.9054199636	6.4769994799
C	-0.2999509433	-3.554021853	2.7617974335
C	-0.8096338683	-3.2446986501	4.1783273483
C	-1.4803110864	-3.6585274552	1.7816507724
H	0.2450176128	-4.509433449	2.793449385
C	-1.8233008749	-4.2988421505	4.6351279165
H	-1.2860717582	-2.2525567287	4.1846996215
H	0.026935411	-3.2041020186	4.88365527
C	-2.4813013336	-4.7232252891	2.2392846819
H	-1.987198058	-2.6834513698	1.7378580995
H	-1.1313104586	-3.877630513	0.7664401998
C	-2.9879520441	-4.4285839008	3.6520254021
H	-2.1922778456	-4.0430104204	5.6344072583
H	-1.3128498972	-5.2681610383	4.7213015782
H	-3.3178329876	-4.7701517261	1.5335695805
H	-1.99272859	-5.7076482061	2.2241930598
H	-3.6762205764	-5.2145137415	3.9817813656
H	-3.5570552791	-3.4883865406	3.6399290059
H	-1.5298036413	7.3014325656	1.1681228141

TS1dC2-to
E(RM062X) = -1671.54277554
Sum of electronic and thermal Free Energies = -1671.041161
Charge = 0 Multiplicity = 1 NIMAG = 1

C	0.0118199667	0.0011126767	0.0605513079
C	0.0357802649	-0.0146966741	1.5276540967
H	1.0260511603	-0.0194543701	1.9889141716
H	-0.6548519031	-0.7272453815	1.9833115669
N	-0.5685162864	1.2998304581	1.3568630636
C	0.3438952034	2.3138856069	1.3259187261
O	1.5768741435	2.1829699574	1.4082551656
C	-0.251224062	3.6851871203	1.1670103464
C	0.5944979084	4.7941921167	1.1482889519
C	-1.6327340418	3.8941553924	1.0335114784

C	0.096624905	6.0890081331	1.0010268735
H	1.6633095939	4.6340196677	1.2510713585
C	-2.1450288034	5.173350152	0.8836930527
H	-2.3017650213	3.0403302792	1.0512297902
C	-1.2827961553	6.2795514031	0.8681248783
H	0.7823609189	6.9281309266	0.9914517941
H	-3.2123872109	5.3432365149	0.7799895976
H	0.7926536162	0.5840552757	-0.4180021795
C	-1.2189828478	-0.3577322327	-0.7289328249
H	-1.882622311	0.5087279767	-0.7888953341
H	-0.9532500852	-0.6497367627	-1.7479121506
H	-1.7634732829	-1.1790437583	-0.2530735179
P	1.2941259291	-2.1202840127	-0.422933128
C	2.0451465309	-2.5188492652	-2.0471266465
C	0.2281699992	-3.5549164453	-0.0415333956
C	2.3684220286	-1.4465223896	-2.8883602044
C	2.3309912066	-3.8260277362	-2.4669485333
C	-0.6404498951	-4.0426655187	-1.0310824976
C	0.1370036218	-4.0824675467	1.2538892532
C	2.9804012091	-1.6726085888	-4.122217316
H	2.141459598	-0.4285090507	-2.5787819806
C	2.9342866696	-4.0509468601	-3.7022476264
H	2.082012939	-4.6698030891	-1.8283061388
C	-1.5588550438	-5.0474532269	-0.7369751699
H	-0.5963852646	-3.637886018	-2.0397309947
C	-0.7860633032	-5.0871411145	1.5466808882
H	0.7862559713	-3.7179787446	2.0453651134
C	3.2617553289	-2.9749592917	-4.5303819295
H	3.2284438983	-0.8324288683	-4.7637014253
H	3.1519459934	-5.0670673419	-4.0176639495
C	-1.6338071601	-5.5743891153	0.5534946469
H	-2.2181174708	-5.4171708384	-1.5167267582
H	-0.8384903968	-5.4884729181	2.5543839925
H	3.7321438037	-3.1539383912	-5.4926662231
H	-2.3498878785	-6.3576634471	0.7825428641
C	2.7031143707	-2.1854451583	0.7836348185
C	3.5598676881	-3.4551047396	0.7353138509
C	3.5728840967	-0.9291833048	0.6077217374
H	2.2207690791	-2.1154591279	1.7704840741
C	4.6525261489	-3.4071262926	1.8092291011
H	4.03223045	-3.5273174139	-0.2538957444
H	2.9382080743	-4.3491357674	0.8617408668
C	4.6741918369	-0.8851783381	1.671928781
H	4.0336809239	-0.9489399241	-0.3908662391
H	2.9571428486	-0.0227220391	0.6685311911
C	5.5234905354	-2.158078741	1.6545728588
H	5.2683158207	-4.3116685216	1.7518394269
H	4.1819960937	-3.4022155746	2.8026108462
H	5.303724345	-0.002437157	1.5150655604
H	4.2069793575	-0.7720121737	2.6604705859
H	6.2780809289	-2.1207934784	2.448133895
H	6.0650652267	-2.2157479799	0.6998265478
C	-1.0389894794	8.6392573435	0.7128364355
H	-0.3317636242	8.6075362183	-0.1233591669
H	-1.7048500496	9.4935448047	0.5918971627
H	-0.4906751719	8.735126142	1.6565100926
O	-1.8742451333	7.4908688018	0.7210662668

TS1dC3-to
E(RM062X) = -1671.54539905
Sum of electronic and thermal Free Energies = -1671.042476
Charge = 0 Multiplicity = 1 NIMAG = 1

C	0.0270604939	-0.0242746071	-0.0058060089
C	0.0080266287	-0.0337921735	1.4628842976
H	1.0308236017	-0.0049418593	-0.4344298069
H	0.8005085825	0.4798213671	1.9965376379
H	-0.9339851564	-0.20539372	1.9707161922
C	-0.915895492	-0.9610574942	-0.7240479635
H	-1.0340822768	-0.6451542825	-1.7648935201
H	-0.5225408353	-1.9838383243	-0.7173734
H	-1.9017548132	-0.9560780396	-0.2477378765
N	-0.5349404356	1.3007347038	0.2387037533
C	0.3906235126	2.3055654308	0.2917564546
O	1.6203197817	2.165592518	0.2013001281
C	-0.1868676066	3.6802627779	0.4863890241
C	0.6829238333	4.7765362739	0.5790848306
C	-1.5602926536	3.9102677478	0.5797884986
C	0.1953326397	6.0604331356	0.7627406017
H	1.751563049	4.6010261014	0.5048700063
C	-2.0695291765	5.1963196799	0.7650477763
H	-2.2431343705	3.0706968493	0.5039878231
C	-1.1871940271	6.2776364639	0.8583268042
H	0.863945999	6.9128042836	0.8357034679
H	-3.141490927	5.3403105801	0.8318157325
P	0.8770557842	-2.2200604303	2.2258331633
C	1.777779782	-3.0106329697	0.8429920429
C	2.1574428246	-2.115305681	3.5338207555
C	2.7476477503	-2.2295335697	0.1959125173
C	1.5408629702	-4.3122423892	0.3865854245
C	2.0809204006	-1.0613551643	4.4525801477
C	3.1824839448	-3.0620085933	3.6593388199
C	3.4674762963	-2.7398712496	-0.8808620139
H	2.9468441651	-1.2165145849	0.5413531791
C	2.2580291549	-4.8197498534	-0.6988300453
H	0.8031037183	-4.9454839054	0.8701800064
C	3.0095983316	-0.9594959501	5.4888165856
H	1.2917542291	-0.3181206464	4.3606334042
C	4.110897717	-2.9574683209	4.6933547054
H	3.2593026526	-3.8795284201	2.9464296604
C	3.2199098657	-4.0370093548	-1.3342228793
H	4.2168202416	-2.1233765793	-1.3680765705
H	2.0639621244	-5.8313229161	-1.0429454063
C	4.0253232125	-1.9070032081	5.6089844858
H	2.9421486051	-0.1378351768	6.195429935
H	4.9030729448	-3.6949082237	4.7831366267
H	3.7752590882	-4.4344484169	-2.1783752576
H	4.752536357	-1.82532751	6.4113053768
C	-0.3211757016	-3.4929434538	2.8538215784
C	-0.7937431686	-3.138089484	4.2724975102
C	-1.5234313406	-3.5908552328	1.8999502017
H	0.2008478538	-4.4605282333	2.8972433248
C	-1.8245544998	-4.1546958927	4.7741947682
H	-1.2446620425	-2.134043545	4.2654281953
H	0.0579453377	-3.1031179821	4.9598055034
C	-2.5414966215	-4.6183645527	2.4027528

H	-2.0066076736	-2.6045165748	1.8428373705
H	-1.2004566265	-3.8421932391	0.8835158406
C	-3.0117781527	-4.2771457224	3.8175962704
H	-2.1664220997	-3.8658467146	5.7741251125
H	-1.3373968762	-5.1346669212	4.8736537869
H	-3.3930617668	-4.660342094	1.7149378801
H	-2.0785190213	-5.6151980033	2.4020819367
H	-3.713168936	-5.0367490502	4.1798152838
H	-3.5567238944	-3.3229543879	3.7939762189
O	-1.574968181	7.5642740866	1.0399422733
C	-2.9661651243	7.8286256809	1.1464176359
H	-3.0568589485	8.9049521943	1.2918495763
H	-3.4947829423	7.5390565859	0.2313852934
H	-3.4024652758	7.307123512	2.0055177309

TS1eC2-to
E(RM062X) = -1748.74021886
Sum of electronic and thermal Free Energies = -1748.218793
Charge = 0 Multiplicity = 1 NIMAG = 1

C	0.1135464521	0.070277429	0.0174317572
C	0.3271264969	0.1941316763	1.4657044199
H	1.3722787125	0.3163010371	1.757999365
H	-0.1961402771	-0.5343034299	2.0869495759
N	-0.4276797694	1.4271865473	1.3024565344
C	0.3553064863	2.5186782373	1.0703567332
O	1.5946287555	2.5259944219	1.0005785101
C	-0.4091240228	3.8055994832	0.8847840567
C	0.3047512302	5.0047036053	0.792203718
C	-1.8054786538	3.8292142919	0.7917299665
C	-0.3656018365	6.2131973986	0.6145786306
H	1.3875403736	4.9723967951	0.8617067286
C	-2.4755836697	5.0373284226	0.6061829188
H	-2.3557868776	2.8968130442	0.8629506632
C	-1.7581392042	6.2315314469	0.5196943873
H	0.1966039246	7.1401358265	0.5483757319
H	-3.5589682173	5.0473683929	0.5300542732
H	0.7795540616	0.6596603156	-0.605353122
P	1.4327176415	-2.0448878475	-0.4753534558
C	2.1947295744	-2.4563647336	-2.089466278
C	0.2296586169	-3.3760540645	-0.154998926
C	2.6089881456	-1.3918313474	-2.9002660965
C	2.4051252026	-3.7716270811	-2.5284896541
C	-0.6069297556	-3.8205392988	-1.1903645902
C	-0.0234125062	-3.8132514658	1.1529463971
C	3.2333590532	-1.6345382338	-4.1243968662
H	2.4461285043	-0.367135441	-2.5730140819
C	3.0220696003	-4.0123874435	-3.7541334488
H	2.0854626766	-4.6088784063	-1.913348204
C	-1.6599967442	-4.6923162545	-0.9248898737
H	-0.4422960174	-3.4780897044	-2.2092359131
C	-1.0797291329	-4.6859501166	1.416004752
H	0.6002550957	-3.476893782	1.9773696324
C	3.437901631	-2.9448477491	-4.5527287962
H	3.5522279518	-0.8006747261	-4.7423387027
H	3.1802489323	-5.0344371679	-4.0853368484
C	-1.901091295	-5.1270242753	0.3792822253
H	-2.2987763723	-5.0241913329	-1.7382700438

H	-1.2588932685	-5.0189111553	2.4340371407
H	3.9180770334	-3.1362420853	-5.5077504717
H	-2.7249355239	-5.8034478621	0.5859706066
C	2.7923379617	-2.2436709241	0.7733104613
C	3.4757287515	-3.6156950556	0.7992932124
C	3.8259622528	-1.1234985379	0.5698737695
H	2.2978274241	-2.0691956745	1.7416510572
C	4.5445398103	-3.6624091031	1.8977829819
H	3.9531242748	-3.7929619453	-0.1742372129
H	2.7391871047	-4.4132252688	0.949130288
C	4.8965632149	-1.1726263445	1.664281494
H	4.3059473261	-1.253073289	-0.410746599
H	3.3341522553	-0.1427667064	0.5622518063
C	5.5734673814	-2.5440319951	1.7179661838
H	5.0383293474	-4.6405180924	1.891447379
H	4.0582210002	-3.555104455	2.8776289063
H	5.6387191758	-0.385937759	1.490427255
H	4.4256455355	-0.9600936816	2.6343267582
H	6.3093289748	-2.5716082334	2.5291993876
H	6.1230215516	-2.7099167281	0.7806917135
H	-2.2820455022	7.1725469094	0.3788266431
C	-1.1386092805	-0.4132774993	-0.5991466561
C	-1.2759030227	-0.3418508438	-1.9912532731
C	-2.1730501334	-0.9743031364	0.1609809115
C	-2.4227690735	-0.8264425737	-2.615554935
H	-0.4721360415	0.0892795508	-2.5841588818
C	-3.3220554584	-1.4521172329	-0.4634973766
H	-2.0889980152	-1.0282268776	1.2423235327
C	-3.4493455101	-1.3838547219	-1.8521836867
H	-2.5157202467	-0.7666208795	-3.6957419742
H	-4.118277532	-1.8836923235	0.1357307681
H	-4.3448982719	-1.7618251746	-2.3362310246

TS1eC3-to
E(RM062X) = -1748.73923795
Sum of electronic and thermal Free Energies = -1748.217593
Charge = 0 Multiplicity = 1 NIMAG = 1

C	0.5416456847	0.2425159604	0.2353719622
C	-0.3271368829	-0.1925598763	1.3443824324
H	1.5744153752	0.4384222913	0.5278297931
H	-0.1366801426	0.1974227157	2.3380063793
H	-1.3117747118	-0.5815938089	1.1121198717
N	-0.3483976567	1.3914123508	0.3362729127
C	0.1344925301	2.3927239135	1.1326138578
O	1.1985209061	2.3649844265	1.7664026497
C	-0.7538310948	3.6081687611	1.2216211348
C	-0.3307162149	4.6893633655	2.0016071319
C	-1.9808500361	3.6834919065	0.553002376
C	-1.1211683904	5.8309591272	2.1137657535
H	0.6230866952	4.6188106571	2.5147565524
C	-2.773460417	4.8243000715	0.6677862094
H	-2.3050709211	2.845754557	-0.05490807
C	-2.345671415	5.9001573073	1.4472192439
H	-0.7837054967	6.6662941771	2.720308305
H	-3.7248642665	4.8751743645	0.1464725581
P	0.4173343356	-2.4415134568	2.0976079151
C	-0.3386648667	-3.9260385199	1.3314178734

C	2.2046996749	-2.6218389086	1.7379651067
C	-0.7986726258	-3.8175277141	0.0124617369
C	-0.4564459975	-5.1565500492	1.9922645854
C	3.037245562	-1.5190677479	1.9850194711
C	2.7520532038	-3.7663218701	1.1453054167
C	-1.3523694963	-4.9196616573	-0.6390599689
H	-0.7207197353	-2.8688933458	-0.512897872
C	-1.0193355368	-6.2546139229	1.3435708103
H	-0.1074608979	-5.270882355	3.014509918
C	4.3915322195	-1.5664303532	1.6611908412
H	2.6246195481	-0.6096972317	2.4170584152
C	4.1065946407	-3.8080362997	0.8127428569
H	2.1221921151	-4.624158026	0.9267858829
C	-1.4653818369	-6.1395268731	0.0261534779
H	-1.6994385556	-4.8188455485	-1.6633443747
H	-1.1058506642	-7.201783994	1.8676789153
C	4.9283021218	-2.7109756467	1.0695883533
H	5.021953315	-0.7044784216	1.8579498932
H	4.5175379459	-4.699583867	0.348279276
H	-1.901948142	-6.99709591	-0.4770319563
H	5.9808577706	-2.7445815296	0.8048856908
C	0.2390609812	-2.7087770012	3.9307960564
C	1.028509964	-1.6611537	4.7308652867
C	-1.254552999	-2.6552749579	4.292497994
H	0.6441562307	-3.7000466017	4.1792127458
C	0.8047236614	-1.8416144726	6.2362405134
H	0.7051909123	-0.6517656562	4.4359871736
H	2.0970351939	-1.7381278664	4.5067602626
C	-1.4673851193	-2.8333284687	5.7983933609
H	-1.6581783417	-1.6791304901	3.9841069082
H	-1.8125978852	-3.4198075126	3.7406550652
C	-0.6808690383	-1.7914841486	6.5947477188
H	1.3578448997	-1.06961411	6.7822485721
H	1.2191000354	-2.81146778	6.545023569
H	-2.5363611619	-2.7673360002	6.0287557172
H	-1.1375694897	-3.84000081	6.0912814934
H	-0.8175775077	-1.9511611881	7.669949526
H	-1.0740822949	-0.7908535656	6.3663779545
H	-2.9634349434	6.7893483586	1.5334499392
C	0.4388344907	-0.5483539085	-1.0381085817
C	1.5046417181	-1.366739248	-1.4249759442
C	-0.7327483842	-0.5457459347	-1.8016209382
C	1.3930637278	-2.1916916389	-2.5445049419
H	2.4225430777	-1.3651986271	-0.8406703987
C	-0.8427191848	-1.3652866633	-2.9247349481
H	-1.5544699264	0.1012909081	-1.5080603117
C	0.2167819929	-2.1963099237	-3.2945292058
H	2.2253787363	-2.8289242342	-2.8292147575
H	-1.7567120729	-1.3562368156	-3.5116793483
H	0.1281178802	-2.8381168171	-4.1661759473

Int1a-C2
E(RM062X) = -1761.54964732
Sum of electronic and thermal Free Energies = -1761.070569
Charge = 0 Multiplicity = 1 NIMAG = 0

C	-0.9186676672	-1.3509309929	-0.4966977097
C	-0.1382072008	-0.0908813162	-0.9714623518

H	-0.5452368663	0.7626552594	-0.4131262552
N	-2.3470033764	-1.1552638633	-0.6246994336
C	-2.8567793242	-0.367961832	0.3019042639
O	-2.2504674351	0.2186168676	1.244115428
C	-4.3602065249	-0.1532120332	0.2060532032
C	-4.9850504246	0.655857887	1.1609169442
C	-5.1326709231	-0.738198517	-0.8058696831
C	-6.3556624495	0.8862247852	1.117542997
H	-4.3733639671	1.1001541127	1.9384754511
C	-6.5033875406	-0.5221675652	-0.869186853
H	-4.6435517262	-1.3656173092	-1.542227692
C	-7.0907022252	0.2901254926	0.098055984
H	-6.8479973023	1.5109212949	1.8534282741
H	-7.1100664425	-0.9697862471	-1.6474441781
N	-8.5360964395	0.5243270073	0.0395561741
O	-9.1720556158	-0.0136588492	-0.8547531849
O	-9.0388380361	1.2467580742	0.8875251179
H	-0.6341340313	-1.5968917881	0.5365983917
H	-0.6192654635	-2.1922201287	-1.1331058481
C	-0.3246237466	0.1242242924	-2.4775356724
H	0.0308361738	1.0978803101	-2.8173988064
H	0.1774397682	-0.6476803106	-3.0685524638
H	-1.3960033808	0.0610317033	-2.6849599854
P	1.6236393907	-0.1584899675	-0.4322593844
C	2.2563678933	-1.8591285866	-0.4750051422
C	2.6806228435	0.8196188438	-1.5378684331
C	2.0570364393	-2.6315897945	-1.6283832922
C	2.9887095335	-2.388690893	0.5934177813
C	2.2715360052	2.1029088034	-1.9290262245
C	3.9184998836	0.3240409844	-1.9620444565
C	2.572401401	-3.9221148347	-1.7023583436
H	1.5081770277	-2.230992113	-2.475062979
C	3.5032747614	-3.6822936949	0.512673762
H	3.1675019541	-1.8083508138	1.4927555506
C	3.094207281	2.8778168221	-2.7408160657
H	1.3140567187	2.4995041874	-1.6014778235
C	4.7383811801	1.1070283269	-2.7745587845
H	4.2472412535	-0.6679899928	-1.6665067657
C	3.2942667331	-4.4495586207	-0.6310447237
H	2.4092880263	-4.5135920297	-2.5975286736
H	4.0669203867	-4.0863431	1.3474956861
C	4.3273515739	2.3797967149	-3.1648305326
H	2.771098653	3.8689956243	-3.0427477935
H	5.6966713236	0.7171396689	-3.1027192001
H	3.6940973144	-5.4571894701	-0.6898470371
H	4.9661621415	2.9854155036	-3.800358804
C	1.8065701756	0.50481672	1.2728340683
C	1.5865447593	2.0277388094	1.3296360553
C	0.8822873238	-0.2146517089	2.273064773
H	2.8605886743	0.3111422757	1.5224284324
C	1.8077197061	2.5403302545	2.757264685
H	0.5615078005	2.2631417125	1.0120034266
H	2.273945913	2.5420501043	0.6523114445
C	1.1345496411	0.3053470641	3.6914953035
H	-0.1624967488	-0.0217424518	1.9939896861
H	1.0353304485	-1.2987080569	2.2399539788
C	0.9182316648	1.8164751271	3.767200105
H	1.6212249656	3.6191821796	2.7819520797

H	2.8622056824	2.3906332058	3.0279767237
H	0.465211989	-0.2126553396	4.3863946386
H	2.1639689757	0.0656890291	3.9947165974
H	1.1218623022	2.1820912126	4.7795177402
H	-0.1349998248	2.0388719606	3.5482128297

Int1a-C3
E(RM062X) = -1761.55170977
Sum of electronic and thermal Free Energies = -1761.074468
Charge = 0 Multiplicity = 1 NIMAG = 0

C	-0.9228443114	-1.5484465979	0.2485215375
N	-2.3662262005	-1.3672611495	0.1967530241
C	-2.8535399063	-0.4091106294	0.9588673539
O	-2.2329995597	0.4033571064	1.7087854836
C	-4.3699862502	-0.2698585486	0.9121901599
C	-5.173093408	-1.1065999276	0.1261606166
C	-4.975410911	0.7259933065	1.68601241
C	-6.5539249062	-0.9561991653	0.1079746792
H	-4.7007268137	-1.8787217263	-0.4700900527
C	-6.355612563	0.8950372983	1.6841867851
H	-4.3406468409	1.3644109438	2.2905618083
C	-7.1210403765	0.0466354906	0.8909025486
H	-7.1831938775	-1.5989236224	-0.496239127
H	-6.8322668983	1.6637286722	2.2809793525
N	-8.5768351886	0.2132126578	0.8789509791
O	-9.0633106656	1.090797363	1.5766895853
O	-9.2374667464	-0.5329491759	0.1714720234
H	-0.6790254444	-2.3459540066	-0.463842764
C	-0.4523312345	-2.0421058137	1.624208198
H	-0.9298594294	-3.0080801019	1.8157530837
H	0.6317063863	-2.1999677072	1.6672196922
H	-0.7458482333	-1.3534086337	2.4173327923
H	-0.6444950937	0.6217859462	0.1818417617
H	-0.4487367674	-0.190049932	-1.3736983319
C	-0.2301786791	-0.2684809707	-0.3018014254
P	1.5691332944	-0.1333392759	-0.1469845809
C	2.3252961902	-1.7556696699	-0.434916875
C	2.0136872923	0.521057409	1.4791922827
C	1.8786166154	-2.4970680298	-1.538550211
C	3.300878593	-2.2884511893	0.4139693098
C	1.0179963435	0.8470577055	2.4082742908
C	3.3605681046	0.7906598754	1.7634146015
C	2.4212451219	-3.7525616185	-1.7981398128
H	1.1011278822	-2.1055010152	-2.1897894215
C	3.8400197913	-3.5458309606	0.1466546117
H	3.631237092	-1.7445027054	1.2926241822
C	1.3825008998	1.4189564137	3.627788381
H	-0.0354295413	0.6712315592	2.19349685
C	3.7117586591	1.3593835547	2.983832298
H	4.1351918931	0.5581809553	1.0365996313
C	3.4048278042	-4.2755564267	-0.9582292989
H	2.0687803943	-4.3232879144	-2.6512995043
H	4.5941924682	-3.9564662095	0.8104185616
C	2.721863235	1.6707522783	3.9176835741
H	0.6105253505	1.6708018309	4.3481532025
H	4.7553211433	1.5619948498	3.2028072838
H	3.8243214808	-5.2562946058	-1.1602881081

H	2.9967352756	2.1166833863	4.8687537453
C	2.2057749496	1.0820287096	-1.3721653617
C	2.107595935	0.600275552	-2.8273590901
C	1.4915656448	2.4317279009	-1.1759664041
H	3.2687545144	1.1966178805	-1.1120492782
C	2.6635546175	1.6687010728	-3.7759967989
H	1.0568393499	0.4025136088	-3.0802037981
H	2.6628671987	-0.33315189	-2.9599109922
C	2.0617117123	3.4807489218	-2.1349907494
H	0.4195119416	2.3056529083	-1.3789579468
H	1.5908561588	2.7719730317	-0.1392266633
C	1.961713753	3.0136577974	-3.5872943633
H	2.5592811789	1.3212752829	-4.8091504172
H	3.7385693916	1.7898736903	-3.5845855743
H	1.5260087548	4.4255624052	-1.996342984
H	3.1144165891	3.6664130456	-1.8817257266
H	2.3958529328	3.7631951807	-4.2574874621
H	0.9026219132	2.9118676283	-3.8617405575

TS2a-C2
E(RM062X) = -1761.49476615
Sum of electronic and thermal Free Energies = -1761.021429
Charge = 0 Multiplicity = 1 NIMAG = 1

C	-0.5240389799	-0.8951686096	0.1376695585
C	-0.1135058458	0.5251628734	-0.253239698
H	0.0761931697	1.2735935335	0.5020554706
N	-1.9602720089	-1.1303842916	0.0218118481
C	-2.6119412509	-0.0073649805	0.0280667976
O	-2.045513962	1.1466342132	0.055533109
C	-4.1147111239	-0.0463273194	-0.0217457522
C	-4.8253733609	1.1566507241	-0.0733422931
C	-4.8081541752	-1.2629829524	-0.0169213292
C	-6.2152087837	1.1569784497	-0.1224791799
H	-4.2753971168	2.0910878814	-0.0754697332
C	-6.1956957555	-1.2832279286	-0.0656156563
H	-4.2494554838	-2.1913387203	0.0244781124
C	-6.8731314404	-0.0675663312	-0.117811902
H	-6.7782365163	2.0816482896	-0.1639108016
H	-6.7458300323	-2.2165141906	-0.0635377069
N	-8.339868451	-0.0798952541	-0.1707545577
O	-8.9062365182	-1.1619090742	-0.1652695724
O	-8.9231769306	0.992157257	-0.21788147
H	-0.1961693936	-1.1031811588	1.1652548113
H	-0.0109382088	-1.609624249	-0.5148481573
C	-0.1785526422	0.9100491573	-1.7147510119
H	-0.2735881331	1.9910777626	-1.8287349331
H	0.6917047457	0.5722133705	-2.2828461518
H	-1.0609868041	0.4368661336	-2.1556741439
P	2.2859070951	0.1065292258	-0.0019261528
C	2.8181738944	-1.6280913501	-0.2178495609
C	3.3576916206	1.0310892667	-1.1637800338
C	2.4829393481	-2.2518004686	-1.4301170407
C	3.5156164724	-2.3574444447	0.751441235
C	2.9429950145	2.3055168398	-1.5757787926
C	4.5739401876	0.5221638207	-1.6367002047
C	2.8471737266	-3.573432713	-1.6711688552
H	1.9417394602	-1.6972731066	-2.1944643908

C	3.8729119745	-3.6853727228	0.5106409093
H	3.7929582432	-1.9010453781	1.6966060368
C	3.7352521552	3.0625653029	-2.4367672513
H	1.9993618307	2.7119234808	-1.2189557938
C	5.3627935707	1.2797458823	-2.5019839297
H	4.9089346452	-0.465524862	-1.3312858099
C	3.5416686115	-4.2944202654	-0.6979387254
H	2.5851235367	-4.0412018326	-2.6152415215
H	4.4144155902	-4.2402498898	1.2709063828
C	4.945593868	2.5489189936	-2.9028672943
H	3.4041650434	4.0490332813	-2.7468391296
H	6.3042968543	0.8762431091	-2.8623668964
H	3.8214686465	-5.3272088774	-0.8821011605
H	5.5607466741	3.1352072718	-3.5787951929
C	2.8472410314	0.62188216	1.6911300968
C	2.884346599	2.1530306536	1.8231358474
C	1.9187356362	0.0172542499	2.7592322781
H	3.8702565544	0.2425921064	1.8308585165
C	3.3127238875	2.5643876592	3.2359392967
H	1.8862779759	2.5641967478	1.6086729662
H	3.5747695075	2.5824405452	1.0906057374
C	2.3711612864	0.4200512407	4.1652323957
H	0.8983619048	0.3915135882	2.5933216532
H	1.8740787776	-1.0740201626	2.671105295
C	2.4168133239	1.9419801937	4.3077614936
H	3.3040484772	3.6568653083	3.3158736915
H	4.349894694	2.2400190171	3.3989227444
H	1.6938523015	-0.0151506487	4.9078685772
H	3.3701058966	0.0035163814	4.3567516395
H	2.7712681756	2.2214663213	5.305892136
H	1.3982699096	2.3418924891	4.2053831504

TS2a-C3
E(RM062X) = -1761.49905673
Sum of electronic and thermal Free Energies = -1761.024135
Charge = 0 Multiplicity = 1 NIMAG = 1

C	-0.5312760765	-1.1467011883	0.2941135803
N	-1.9672773259	-1.2788508879	0.0390375515
C	-2.5751165169	-0.1609875484	0.2867473907
O	-1.9583406423	0.9145414118	0.6410868275
C	-4.0695524986	-0.0956978299	0.150820916
C	-4.8092274271	-1.2279494843	-0.2118080651
C	-4.726456744	1.1134542891	0.3977658399
C	-6.1907119812	-1.1580802113	-0.3310359563
H	-4.290319825	-2.161486181	-0.3987387815
C	-6.1098450885	1.2024437128	0.2860112864
H	-4.1414143497	1.9820452501	0.678965015
C	-6.8146693401	0.0609040677	-0.0779637402
H	-6.7768044462	-2.0250843166	-0.611274179
H	-6.6327783714	2.1322833581	0.4752358466
N	-8.2750886566	0.1434752944	-0.1993794713
O	-8.8128058255	1.2125671562	0.0443828933
O	-8.8815784795	-0.8608340244	-0.538479076
H	-0.0152784385	-1.6608764859	-0.5212822346
C	-0.1686017988	-1.838621726	1.6066705092
H	-0.5490404267	-2.8634721798	1.596978797
H	0.9171252353	-1.8780552451	1.7529455116

H	-0.6219749948	-1.310879037	2.4535806857
H	0.1532277219	0.8588941111	1.1977455004
H	-0.1432579185	0.8968170783	-0.6295724781
C	-0.1009412961	0.3257766876	0.2909844577
P	2.2393549326	0.0657305505	-0.0571569581
C	2.956863389	-1.5746593792	-0.4550418998
C	3.1733253661	0.6208758673	1.4184893151
C	2.3898582709	-2.3307162935	-1.494024338
C	4.0112393571	-2.1278416123	0.2847833871
C	2.5791614851	0.4973894262	2.6800571759
C	4.4717734566	1.1441541665	1.3276521243
C	2.8860716796	-3.5956241741	-1.8029030148
H	1.5545813958	-1.9378765668	-2.0673227654
C	4.5004796781	-3.3971123106	-0.0230058436
H	4.4555749369	-1.5737362751	1.1058351728
C	3.2696685066	0.8827672929	3.8304345344
H	1.5746518422	0.0953937669	2.7758821524
C	5.1580837254	1.5324256557	2.4755061586
H	4.9595555138	1.2449059858	0.3619793555
C	3.9433621401	-4.1319793634	-1.0682057706
H	2.438049646	-4.1639334207	-2.6122742982
H	5.3185708926	-3.8103603745	0.5593119554
C	4.558427691	1.4011429886	3.729596622
H	2.795581633	0.7789796807	4.8015760281
H	6.1631524702	1.9344946302	2.3906546303
H	4.3253904132	-5.1203921053	-1.3048060649
H	5.0955654489	1.703713107	4.6234767141
C	2.7771741506	1.2261980056	-1.4083343092
C	2.1615664981	0.8371586355	-2.7615171872
C	2.3884064479	2.6638083015	-1.0197040733
H	3.8700936157	1.1566470652	-1.499144179
C	2.5414536921	1.8481592245	-3.8490382245
H	1.0661043858	0.7955377766	-2.6694806282
H	2.501954558	-0.1586864573	-3.0600740892
C	2.7775602708	3.6569042148	-2.1184967611
H	1.3019320668	2.715579284	-0.8603848615
H	2.863381957	2.9472036726	-0.0746102315
C	2.1498495112	3.2730728417	-3.4584827261
H	2.0641710689	1.5624384372	-4.7926704016
H	3.6271066695	1.8038162046	-4.0129993262
H	2.4706206774	4.6661712375	-1.8235133202
H	3.8717614924	3.6704312026	-2.2201410857
H	2.4560380408	3.9781409186	-4.2389498602
H	1.0557655373	3.3392811205	-3.376952735

Table S1: Summary of the energies and NIMAG of all structures optimized at the SMD(THF)-M06-2X/6-31+G(d,p) level of theory.

	T = 298.15 K	T = 343.15 K			
	Hartree	Hartree	Hartree		cm^{-1}
System	Gibbs Free Energy (QRRHO)	Gibbs Free Energy (QRRHO)	Energy	NIMAG	Frequency
1a-ti	-721,741978	-721,749992	-721,896204	0	--
1a-to	-721,741287	-721,749303	-721,895486	0	--
1a-ci	-721,741151	-721,749114	-721,895849	0	--
1a-co	-721,739781	-721,747784	-721,894031	0	--
3a	-721,759017	-721,768265	-721,914710	0	--
2a	-721,760449	-721,766979	-721,917187	0	--
1b-ti	-976,887303	--	-977,030667	0	--
1b-to	-976,886836	--	-977,029964	0	--
1b-ci	-976,886526	--	-977,030279	0	--
1b-co	-976,884692	--	-977,028366	0	--
1c-ti	-517,306143	--	-517,461014	0	--
1c-to	-517,305535	--	-517,460277	0	--
1c-ci	-517,305419	--	-517,460580	0	--
1c-co	-517,303257	--	-517,458662	0	--
1d-ti	-631,762104	--	-631,947169	0	--
1d-to	-631,761567	--	-631,946501	0	--
1d-ci	-631,761080	--	-631,946752	0	--
1d-co	-631,758974	--	-631,944630	0	--
1e-ti	-708,934369	--	-709,138724	0	--
1e-to	-708,932949	--	-709,137042	0	--
1e-ci	-708,931544	--	-709,136353	0	--
1e-co	-708,929233	--	-709,133823	0	--
P(Me)3	-460,913384	--	-460,997596	0	--
PCyPh2	-1039,331864	-1039,341015	-1039,634063	0	--
TS1a-C2-ti	-1760,999428	--	-1761,481009	1	-559,3965
TS1a-C2-to	-1761,016443	-1761,029922	-1761,495486	1	-548,0883
TS1a-C2-ci	-1761,001772	--	-1761,481559	1	-555,6043
TS1a-C2-co	-1761,012296	--	-1761,491513	1	-566,8821
TS1a-C3-ti	-1761,007313	--	-1761,488521	1	-564,0402
TS1a-C3-to	-1761,018690	-1761,032101	-1761,498290	1	-554,8987
TS1a-C3-ci	-1760,998311	--	-1761,478967	1	-586,3025
TS1a-C3-co	-1761,009597	--	-1761,489366	1	-551,5377
TS1a-C2-ti_P(Me)3	-1182,585900	--	-1182,843741	1	-590,4138

TS1a-C2-to_P(Me)3	-1182,600186	--	-1182,857713	1	-563,8125
TS1a-C2-ci_P(Me)3	-1182,588214	--	-1182,845967	1	-602,2522
TS1a-C2-co_P(Me)3	-1182,598592	--	-1182,856297	1	-564,5941
TS1a-C3-ti_P(Me)3	-1182,590987	--	-1182,849344	1	-584,6386
TS1a-C3-to_P(Me)3	-1182,604492	--	-1182,861838	1	-557,0130
TS1a-C3-ci_P(Me)3	-1182,586611	--	-1182,844452	1	-579,3358
TS1a-C3-co_P(Me)3	-1182,595506	--	-1182,854051	1	-562,8848
TS1b-C2-to	-2016,160058	--	-2016,628225	1	-550,8710
TS1b-C3-to	-2016,163176	--	-2016,630966	1	-558,5352
TS1c-C2-to	-1556,577907	--	-1557,057461	1	-552,1052
TS1c-C3-to	-1556,580646	--	-1557,060191	1	-559,9430
TS1d-C2-to	-1671,032580	--	-1671,542776	1	-555,3812
TS1d-C3-to	-1671,034397	--	-1671,545399	1	-557,0799
TS1e-C2-to	-1748,209694	--	-1748,740219	1	-509,5454
TS1e-C3-to	-1748,208560	--	-1748,739238	1	-566,3209
Int1a-C2	-1761,063531	-1761,076664	-1761,549647	0	--
Int1a-C3	-1761,066735	-1761,079921	-1761,551710	0	--
TS2a-C2	-1761,013064	-1761,026361	-1761,494766	1	-576,7474
TS2a-C3	-1761,016029	-1761,029225	-1761,499057	1	-594,8463

Cartesian coordinates, energies, and NIMAG of all structures optimized at the SMD(THF)-ωB97X-D/6-31+G(d,p) level of theory.

1a
E(wB97xD) = -721.963902
Sum of electronic and thermal Free Energies = -712.812083
Charge = 0 Multiplicity = 1 NIMAG = 0

C	3.05460700	0.89129300	0.68315600
C	4.06776700	-0.10747700	0.21275600
H	4.28519400	-0.96471100	0.84498400
H	4.88183500	0.23085900	-0.42274000
N	2.75351300	-0.05599500	-0.38668700
C	1.84812800	-1.09166800	-0.31494200
O	2.17389500	-2.26786200	-0.38395300
C	0.40763500	-0.67513900	-0.22371800
C	-0.52213500	-1.60265800	0.25345200
C	-0.01184500	0.60156200	-0.60908900
C	-1.86314700	-1.26033400	0.36220400
H	-0.18933400	-2.59329600	0.54268900
C	-1.35150400	0.95620800	-0.51518500
H	0.70287000	1.31627300	-1.00085600
C	-2.25217300	0.01748200	-0.02517400
H	-2.59104700	-1.96823500	0.73871700
H	-1.69002400	1.93893800	-0.81874600
N	-3.66841500	0.38873800	0.08418600
O	-3.99461100	1.52113300	-0.24731600
O	-4.45345700	-0.45179600	0.50288700
C	3.14204800	2.34916500	0.32643200
H	2.14901100	2.80953500	0.32056300
H	3.75536900	2.87675100	1.06363300
H	3.59394300	2.47966700	-0.66128000
H	2.58360000	0.66260700	1.63824800

PPh2Cy
E(wB97xD) = -1039.756270
Sum of electronic and thermal Free Energies = -1039.455982
Charge = 0 Multiplicity = 1 NIMAG = 0

P	-0.06248200	-0.02018000	-1.06459500
C	1.45128000	-0.43106800	-0.05487800
C	1.96991500	-1.83455700	-0.40632200
C	2.54723700	0.62073400	-0.29428900
H	1.19108000	-0.40132300	1.01280400
C	3.24097100	-2.17216400	0.37937500
H	2.18962700	-1.87341300	-1.48291500
H	1.20085700	-2.59062000	-0.21382400
C	3.82036500	0.28239900	0.48832800
H	2.78152400	0.66336500	-1.36789300
H	2.18951600	1.61525200	-0.00575800
C	4.33121500	-1.12158000	0.15667100
H	3.60292700	-3.16485500	0.08804200
H	2.99921400	-2.22313500	1.45044100
H	4.59365000	1.02929000	0.27501600
H	3.60636600	0.34504500	1.56452600
H	5.21446400	-1.35656200	0.76171700
H	4.64840800	-1.15028300	-0.89527800

C	-0.55103700	1.59397100	-0.30944800
C	-0.45242100	2.74685700	-1.09620400
C	-0.97653100	1.72103200	1.01920500
C	-0.76333900	4.00068700	-0.56790900
H	-0.12786300	2.66570100	-2.13056900
C	-1.28998500	2.97029900	1.54851100
H	-1.06859600	0.83906400	1.64748100
C	-1.18289800	4.11395200	0.75538400
H	-0.68093500	4.88552800	-1.19227500
H	-1.62013800	3.05210100	2.58006500
H	-1.42902200	5.08779700	1.16836600
C	-1.36340300	-1.14074600	-0.38431200
C	-2.51066700	-1.31795700	-1.16993000
C	-1.29000500	-1.80887100	0.84481200
C	-3.56278000	-2.12136500	-0.73445100
H	-2.58322900	-0.82003700	-2.13413000
C	-2.33538300	-2.62383700	1.27709600
H	-0.41598500	-1.70208900	1.48000800
C	-3.47620400	-2.77938200	0.49135400
H	-4.44484200	-2.23955800	-1.35694800
H	-2.25742800	-3.13581100	2.23188000
H	-4.29035300	-3.41316600	0.83021700

TS1C2-to
E(wB97xD) = -1761.688599
Sum of electronic and thermal Free Energies = -1761.217479
Charge = 0 Multiplicity = 1 NIMAG = 1 (-524.55)

C	-0.05737000	0.01021800	0.02288400
C	-0.06351200	0.00170200	1.48816500
H	0.91559600	-0.03343700	1.96955900
H	-0.78303300	-0.68476000	1.93664000
N	-0.62475700	1.33095200	1.29424800
C	0.29739600	2.32909500	1.32174300
O	1.51570900	2.20822600	1.52258700
C	-0.27502800	3.70755600	1.07367700
C	0.58427000	4.80927100	1.12086900
C	-1.63071200	3.90313000	0.78870500
C	0.10750500	6.09222900	0.88758700
H	1.63352800	4.64856500	1.34166800
C	-2.12562500	5.17846100	0.54896800
H	-2.29681700	3.04916600	0.75783600
C	-1.24468900	6.25397500	0.60298600
H	0.76820300	6.94959900	0.92340400
H	-3.17348800	5.33769300	0.32626200
N	-1.75857100	7.60252400	0.35114800
O	-2.94990600	7.72954800	0.09463300
O	-0.97320300	8.54116900	0.40774200
H	0.76035000	0.54319800	-0.44838500
C	-1.25738100	-0.34661700	-0.80379800
H	-1.90530000	0.52823700	-0.90471900
H	-0.95537600	-0.65382100	-1.80733900
H	-1.83386300	-1.15098200	-0.33870500
P	1.25716500	-2.21085800	-0.37901600
C	2.02954700	-2.70422200	-1.96478600
C	0.13439300	-3.58897400	0.04594000
C	2.39561200	-1.68325100	-2.84989200
C	2.30062200	-4.03444300	-2.31292100

C	-0.73597800	-4.08563200	-0.93621400
C	0.00281400	-4.06065700	1.35839300
C	3.03203100	-1.98194600	-4.05441000
H	2.18482700	-0.64683700	-2.59862100
C	2.92813300	-4.33290600	-3.51912100
H	2.02155400	-4.84057900	-1.64076800
C	-1.69407800	-5.04409400	-0.61875700
H	-0.66301500	-3.72592600	-1.95920100
C	-0.95941900	-5.01876700	1.67524200
H	0.65074200	-3.69028300	2.14690700
C	3.29717000	-3.30731000	-4.39077500
H	3.31301200	-1.17944200	-4.72962800
H	3.13297200	-5.36759600	-3.77738900
C	-1.80868700	-5.51534600	0.68904600
H	-2.35378300	-5.42107100	-1.39460800
H	-1.04135200	-5.37707700	2.69708400
H	3.78774500	-3.54286100	-5.33049200
H	-2.55628100	-6.26260100	0.93697200
C	2.63960300	-2.25689000	0.85762400
C	3.46369200	-3.54954800	0.89976300
C	3.54504100	-1.03129400	0.64236700
H	2.13906800	-2.12333800	1.82751900
C	4.54234800	-3.47173000	1.98569500
H	3.94719900	-3.69808600	-0.07439000
H	2.81600900	-4.41620200	1.07107500
C	4.63233600	-0.96041600	1.71915500
H	4.01942200	-1.10114600	-0.34652800
H	2.95017200	-0.11054600	0.65486200
C	5.44790800	-2.25390500	1.78493200
H	5.13641200	-4.39271100	1.98179400
H	4.05969200	-3.40771900	2.97105700
H	5.28814100	-0.10482600	1.52305900
H	4.15710800	-0.77946000	2.69335400
H	6.18685400	-2.19396500	2.59204500
H	6.00843000	-2.37445900	0.84724800

TS1C3-to
E(wB97xD) = -1761.691071
Sum of electronic and thermal Free Energies = -1761.220548
Charge = 0 Multiplicity = 1 NIMAG = 1 (-569.42)

C	0.09049100	0.02653200	-0.03381400
C	0.05469400	0.01708900	1.43237500
H	1.09843700	0.05113800	-0.45022600
H	0.85338700	0.49734400	1.98377100
H	-0.87845400	-0.18381400	1.94255600
C	-0.83545200	-0.90539400	-0.77962800
H	-0.93038600	-0.58010500	-1.82006000
H	-0.43602200	-1.92477300	-0.77361200
H	-1.83238500	-0.91363800	-0.32748600
N	-0.47748000	1.34424100	0.23023500
C	0.42075900	2.36857100	0.23402300
O	1.63678000	2.28862800	0.01167100
C	-0.18329900	3.72196500	0.54115500
C	0.65079700	4.84394700	0.53559700
C	-1.54287100	3.87605900	0.83250600
C	0.14483900	6.10652200	0.81428300
H	1.70327300	4.71592500	0.30900300

C	-2.06693700	5.13045600	1.11686100
H	-2.18963200	3.00693900	0.83259400
C	-1.21093700	6.22702200	1.10182000
H	0.78558300	6.97964000	0.80999700
H	-3.11830300	5.25771700	1.34370400
N	-1.75614100	7.55412700	1.39832300
O	-2.95146100	7.64555200	1.65130500
O	-0.99157500	8.51124000	1.38000600
P	0.94509900	-2.24382700	2.19078500
C	1.87682000	-3.02281200	0.82140200
C	2.17726500	-2.18649900	3.54548900
C	2.82921200	-2.22317700	0.17310100
C	1.68638300	-4.33651000	0.38001400
C	2.11703300	-1.12087500	4.45059400
C	3.15033000	-3.17790900	3.72157400
C	3.57687900	-2.72605200	-0.88706500
H	2.99282400	-1.19983800	0.50240700
C	2.42967500	-4.83722800	-0.68906400
H	0.96289200	-4.98507400	0.86368100
C	3.00760400	-1.05105000	5.52118600
H	1.36903400	-0.34247300	4.32278800
C	4.04154300	-3.10614800	4.78915300
H	3.21650600	-4.00663600	3.02172800
C	3.37498100	-4.03534600	-1.32429000
H	4.31216600	-2.09339900	-1.37493500
H	2.26954700	-5.85872900	-1.02103900
C	3.97089500	-2.04339900	5.69073800
H	2.95118800	-0.21952700	6.21716700
H	4.79337600	-3.87929800	4.91664100
H	3.95206500	-4.42741100	-2.15627700
H	4.66861200	-1.98796700	6.52098300
C	-0.29556200	-3.50178700	2.75708200
C	-0.83119600	-3.14565100	4.15418600
C	-1.45336000	-3.59743600	1.74840200
H	0.21156100	-4.47456800	2.82728500
C	-1.88993300	-4.15507200	4.60979800
H	-1.27378800	-2.13912700	4.13112200
H	-0.01238600	-3.11820800	4.88017000
C	-2.50202000	-4.61539000	2.20533400
H	-1.92647800	-2.61007600	1.65709200
H	-1.08493200	-3.86135100	0.75158000
C	-3.03284400	-4.27753900	3.59989700
H	-2.27782600	-3.85945400	5.59101600
H	-1.41361500	-5.13684500	4.73871800
H	-3.32358400	-4.64644400	1.48101400
H	-2.05069700	-5.61728000	2.21914900
H	-3.74801700	-5.03962300	3.92925900
H	-3.57959400	-3.32518600	3.55533400

Int-C2
E(wB97xD) = -1761.746733
Sum of electronic and thermal Free Energies = -1761.266470
Charge = 0 Multiplicity = 1 NIMAG = 0

C	-0.92097000	-1.34487600	-0.48539600
C	-0.13359200	-0.09513900	-0.97783900
H	-0.53076800	0.76654800	-0.42753500
N	-2.34944700	-1.14851400	-0.60219000

C	-2.86663100	-0.36426200	0.32329300
O	-2.26701400	0.22411400	1.26971300
C	-4.37049600	-0.15508900	0.21869000
C	-5.01388900	0.61874700	1.18983700
C	-5.13050800	-0.70776400	-0.81993500
C	-6.38378600	0.84267000	1.13783400
H	-4.41776400	1.04343700	1.98987000
C	-6.50008800	-0.49571800	-0.89291400
H	-4.63317700	-1.30787500	-1.57304000
C	-7.10862300	0.27991600	0.09161800
H	-6.88225500	1.44042600	1.89128000
H	-7.08947800	-0.92156700	-1.69586800
N	-8.54946900	0.50923400	0.02277800
O	-9.17572400	0.00261100	-0.90225400
O	-9.07169800	1.19871300	0.89248500
H	-0.62728100	-1.58719300	0.54505700
H	-0.63364800	-2.19436000	-1.11557200
C	-0.33061900	0.11741400	-2.48276900
H	0.04417900	1.08123700	-2.83028400
H	0.14472600	-0.66788000	-3.07788100
H	-1.40509000	0.08145900	-2.68023600
P	1.62735600	-0.16086400	-0.43759200
C	2.26097000	-1.86068700	-0.47908100
C	2.68596900	0.81977400	-1.53870100
C	2.05015000	-2.63817100	-1.62613900
C	2.99817900	-2.39104200	0.58492100
C	2.28053900	2.10212500	-1.93348200
C	3.92557200	0.32584200	-1.95762200
C	2.55696800	-3.93095500	-1.69819100
H	1.49819300	-2.24116600	-2.47106300
C	3.50411000	-3.68665500	0.50696300
H	3.18839700	-1.81022200	1.48063600
C	3.10582000	2.87593600	-2.74185100
H	1.32345800	2.50122800	-1.61202800
C	4.74854300	1.10683800	-2.76648600
H	4.25483800	-0.66492300	-1.66118300
C	3.28266300	-4.45801900	-0.63080000
H	2.38336100	-4.52511100	-2.58948900
H	4.07105700	-4.08990100	1.33981500
C	4.33998600	2.37873900	-3.15989300
H	2.78343200	3.86667900	-3.04560000
H	5.70811100	0.71624700	-3.08976300
H	3.67579400	-5.46826500	-0.68763800
H	4.98168900	2.98367600	-3.79292200
C	1.80426800	0.50888800	1.26579000
C	1.58852700	2.03332500	1.32166200
C	0.88879800	-0.20686900	2.27752500
H	2.85799000	0.31698100	1.51407100
C	1.81684000	2.55195900	2.74579000
H	0.56543000	2.27317100	1.00518100
H	2.27522700	2.54539600	0.64270000
C	1.14778600	0.31767800	3.69268200
H	-0.15854800	-0.02080100	2.00667700
H	1.04519100	-1.28986200	2.25034300
C	0.93970900	1.83034600	3.76873400
H	1.62418700	3.63000000	2.76672400
H	2.87438200	2.41167800	3.00924300
H	0.47723900	-0.19509000	4.39053000

H	2.17577400	0.07220600	3.99553300
H	1.16066200	2.19550900	4.77785700
H	-0.11551200	2.05808900	3.56740400

Int-C3
E(wB97xD) = -1761.748372
Sum of electronic and thermal Free Energies = -1761.269024
Charge = 0 Multiplicity = 1 NIMAG = 0

C	0.25178300	1.47260200	0.01131300
N	1.67783800	1.31026500	-0.22897400
C	2.28655200	0.35946800	0.45129900
O	1.79242700	-0.47194900	1.27251800
C	3.78470900	0.25599600	0.19433900
C	4.46054700	1.13823900	-0.65802100
C	4.51083100	-0.75153900	0.83741400
C	5.82738800	1.02153100	-0.86831600
H	3.90042100	1.92177300	-1.15472300
C	5.87923800	-0.88769400	0.64156800
H	3.98006100	-1.42770200	1.49830300
C	6.51904500	0.00524000	-0.21269100
H	6.35158500	1.70418300	-1.52613600
H	6.44156800	-1.66833000	1.13951400
N	7.95782400	-0.12557000	-0.42687000
O	8.55581600	-1.01796800	0.16519400
O	8.50689000	0.66241500	-1.18989600
H	-0.09138800	2.26165600	-0.66815100
C	-0.04290600	1.97634400	1.43163000
H	0.46214400	2.93875800	1.56185100
H	-1.11174900	2.14430200	1.60386100
H	0.33600300	1.28983800	2.18967600
H	0.00330600	-0.69811600	-0.01343600
H	-0.41243800	0.09980800	-1.52711500
C	-0.49118700	0.18079200	-0.43728300
P	-2.24909900	-0.00321900	-0.05202900
C	-3.08057500	1.59750400	-0.22878300
C	-2.46594000	-0.67851200	1.61145500
C	-2.80080300	2.35479700	-1.37482200
C	-3.95089900	2.10365500	0.74105400
C	-1.35598500	-0.97969000	2.40896600
C	-3.75736100	-0.98655900	2.06232700
C	-3.40152900	3.59654100	-1.55313200
H	-2.10753500	1.98735400	-2.12549500
C	-4.54965100	3.34735800	0.55550700
H	-4.15384900	1.54972500	1.65074600
C	-1.54836400	-1.56280900	3.66024700
H	-0.34236300	-0.77799600	2.06781100
C	-3.93806600	-1.56646100	3.31297700
H	-4.62405400	-0.77708500	1.44167300
C	-4.27909600	4.09229100	-0.59001700
H	-3.17657700	4.17836100	-2.44109300
H	-5.22160500	3.73580300	1.31406200
C	-2.83255500	-1.85198000	4.11437000
H	-0.68532200	-1.79450400	4.27620500
H	-4.94041200	-1.79829700	3.65853800
H	-4.74434500	5.06327000	-0.72853700
H	-2.97390000	-2.30675900	5.09003400
C	-3.00078000	-1.22967600	-1.19609900

C	-3.10252000	-0.74392700	-2.65055300
C	-2.24163300	-2.56668600	-1.09899100
H	-4.01895100	-1.37014900	-0.80623700
C	-3.75065300	-1.82088800	-3.52773900
H	-2.09984800	-0.51788800	-3.03621600
H	-3.69273400	0.17523800	-2.70645300
C	-2.90432200	-3.62571500	-1.98435500
H	-1.20528300	-2.42062200	-1.42945700
H	-2.20615000	-2.91378500	-0.06107600
C	-3.00795200	-3.15452400	-3.43563000
H	-3.77972300	-1.46897700	-4.56443200
H	-4.79238600	-1.96187900	-3.20881500
H	-2.33012100	-4.55623200	-1.92346900
H	-3.90900200	-3.84291600	-1.59698700
H	-3.51602800	-3.91107800	-4.04344400
H	-1.99743900	-3.03652400	-3.85040400

TS2C2
E(wB97xD) = -1761.689561
Sum of electronic and thermal Free Energies = -1761.215067
Charge = 0 Multiplicity = 1 NIMAG = 1 (-532.20)

C	-0.00860700	-0.03177400	0.00899400
C	-0.08588300	-0.00685100	1.53259800
H	0.81339200	-0.01884900	2.12618000
N	-0.21935800	-1.35872800	-0.56065400
C	-0.01558900	-2.28729200	0.32598000
O	0.24966900	-2.05025900	1.56127400
C	-0.11069800	-3.72463600	-0.10402900
C	0.03112400	-4.73641300	0.85010700
C	-0.33347600	-4.06837400	-1.44261300
C	-0.04966000	-6.07427000	0.48443600
H	0.20583500	-4.46418200	1.88473200
C	-0.41627900	-5.39887200	-1.82693900
H	-0.44116900	-3.28294000	-2.18177600
C	-0.27288400	-6.38379000	-0.85282100
H	0.05913900	-6.86188600	1.21991500
H	-0.58815900	-5.67043500	-2.86118400
N	-0.35914700	-7.78969200	-1.24920000
O	-0.53408100	-8.04838300	-2.43440300
O	-0.25269000	-8.64557400	-0.37821700
H	0.96638500	0.35237600	-0.31487100
H	-0.77072600	0.63804600	-0.40099100
C	-1.43555700	-0.14697300	2.19322600
H	-1.33521200	-0.46129700	3.23299700
H	-2.01204100	0.78051600	2.16258800
H	-2.01317800	-0.90347600	1.65493200
P	0.24146100	2.45436700	1.72195500
C	-0.38303800	3.43973300	0.31693200
C	-0.57824400	3.20447100	3.17597300
C	-1.70212800	3.19165400	-0.09052600
C	0.36615300	4.40374000	-0.36578500
C	-0.68131400	2.44199300	4.34691400
C	-1.09075900	4.50719700	3.16540200
C	-2.26233200	3.89792000	-1.15038000
H	-2.29926600	2.44579600	0.42846700
C	-0.19392300	5.10494600	-1.43327300
H	1.38728200	4.62465500	-0.07281400

C	-1.27403300	2.97517700	5.48884600
H	-0.29283900	1.42772600	4.37111600
C	-1.68835800	5.03743600	4.30705900
H	-1.02723300	5.11287100	2.26618400
C	-1.50675400	4.85556900	-1.82657200
H	-3.28592300	3.69639200	-1.45081200
H	0.39898800	5.85072700	-1.95410600
C	-1.78041700	4.27391900	5.46985100
H	-1.34428600	2.37397100	6.39016200
H	-2.08210500	6.04913800	4.28635600
H	-1.94011900	5.40372000	-2.65756200
H	-2.24832000	4.68871800	6.35752500
C	2.03901600	2.86999100	1.91401100
C	2.57295400	2.42893400	3.28770000
C	2.86386900	2.21801800	0.78972300
H	2.13516300	3.96304600	1.85234200
C	4.06310200	2.75967000	3.42426800
H	2.42733500	1.34585900	3.40802600
H	2.01610700	2.92016500	4.09111700
C	4.34783400	2.56984800	0.92448700
H	2.75110500	1.12725000	0.85261900
H	2.49294100	2.51839100	-0.19598900
C	4.89182200	2.15088600	2.29154900
H	4.42636200	2.40592600	4.39553200
H	4.18628800	3.85151400	3.41711700
H	4.91363400	2.08304000	0.12258000
H	4.47635700	3.65346400	0.79463500
H	5.94187600	2.44781900	2.39046100
H	4.86115100	1.05515300	2.36921200

TS2C3
E(wB97xD) = -1761.694127
Sum of electronic and thermal Free Energies = -1761.217563
Charge = 0 Multiplicity = 1 NIMAG = 1 (-581.36)

C	0.00530200	-0.03559800	0.00832800
N	0.00432200	-0.03291800	1.47244400
C	1.21714400	-0.00133700	1.93162300
O	2.25579500	0.08953400	1.17226500
C	1.44010700	-0.06262200	3.41437000
C	0.36947600	-0.24407600	4.29753800
C	2.73722900	0.05774700	3.92116700
C	0.58502400	-0.30066200	5.66675000
H	-0.63487600	-0.34159200	3.90180500
C	2.97236200	0.00278600	5.28930700
H	3.56282800	0.19478700	3.23228500
C	1.88818100	-0.17516800	6.14156100
H	-0.23825400	-0.44076800	6.35633600
H	3.97522700	0.09635900	5.68734200
N	2.12372100	-0.23356100	7.58466800
O	3.27638500	-0.13260300	7.98880200
O	1.15727200	-0.38013100	8.32398900
H	-0.78062400	0.65384000	-0.30905000
C	-0.34219500	-1.42920000	-0.51337400
H	-1.26689400	-1.77576800	-0.04366300
H	-0.49386000	-1.42385100	-1.59828500

H	0.45408900	-2.14210200	-0.27159100
H	2.06869800	-0.18341200	-0.99918500
H	1.61941300	1.50882800	-0.40636300
C	1.34218000	0.47170600	-0.54087300
P	0.68270800	1.06392000	-2.79024800
C	-1.08672800	1.09773200	-3.26328300
C	1.47086000	-0.04085100	-4.01800600
C	-1.99670300	1.76047100	-2.42534400
C	-1.57579200	0.43344700	-4.39532500
C	1.77969200	-1.35246100	-3.64077000
C	1.76617600	0.37482500	-5.32394700
C	-3.35586100	1.77980600	-2.72703100
H	-1.65128000	2.26557400	-1.52815900
C	-2.93754900	0.44879500	-4.69141100
H	-0.89871600	-0.10285200	-5.05214500
C	2.36708500	-2.23290100	-4.54870500
H	1.56094600	-1.69853800	-2.63561200
C	2.35705300	-0.50241600	-6.22830500
H	1.53091200	1.38424700	-5.64675700
C	-3.83027200	1.12366800	-3.86154700
H	-4.04385400	2.30061500	-2.06815100
H	-3.29876900	-0.07118300	-5.57359700
C	2.65801800	-1.80896600	-5.84257800
H	2.59921000	-3.24757500	-4.24026200
H	2.57976500	-0.16618400	-7.23641200
H	-4.89095900	1.13268100	-4.09324100
H	3.11849100	-2.49248700	-6.54942000
C	1.36806400	2.75008100	-3.16842000
C	0.72307900	3.83771200	-2.29350400
C	2.89679900	2.73240700	-2.98180600
H	1.13825200	2.98060300	-4.21741500
C	1.34778600	5.20987700	-2.56889400
H	0.85583800	3.58641000	-1.23182600
H	-0.35288200	3.88951700	-2.48283100
C	3.50460400	4.10978700	-3.26162100
H	3.13455500	2.43970700	-1.94996400
H	3.35503800	1.98558600	-3.63786800
C	2.86600600	5.19039500	-2.38813600
H	0.89246800	5.95460800	-1.90674000
H	1.10932600	5.51068200	-3.59844900
H	4.58664400	4.07116400	-3.09408300
H	3.35379400	4.36126600	-4.32064400
H	3.28855000	6.17237200	-2.62854800
H	3.10310800	4.98969700	-1.33394700

2a
E(wB97xD) = -721.984710
Sum of electronic and thermal Free Energies = -721.830595
Charge = 0 Multiplicity = 1 NIMAG = 0

C	0.25415900	0.19675300	-0.11344900
C	-0.34936300	-1.05716000	-0.24367900
C	-1.73210600	-1.17753300	-0.18056800
C	-2.48943300	-0.02916600	0.01427300
C	-1.91337800	1.23085700	0.14688900
C	-0.53258600	1.33753100	0.08068000
H	0.25898000	-1.94111400	-0.39401400
H	-2.21157700	-2.14340900	-0.27994400

H	-2.53286100	2.10612600	0.29718000
H	-0.05758800	2.30698300	0.17944200
C	1.72385000	0.33129100	-0.18002800
N	-3.94954600	-0.14891500	0.08378600
O	-4.60310400	0.86932400	0.27124200
O	-4.44399400	-1.26117000	-0.04868300
O	2.41725300	-0.80436400	-0.40043600
N	2.37058600	1.42263700	-0.05094000
C	3.78700700	1.08816000	-0.21942300
C	3.82933400	-0.45424400	-0.30324800
H	4.29886200	-0.80991700	-1.22268500
H	4.36696200	1.46646600	0.62840900
H	4.16752900	1.56761000	-1.12693300
C	4.42910500	-1.13347600	0.91015800
H	4.32562700	-2.22008000	0.84251800
H	5.49496900	-0.89110900	0.97247500
H	3.94105700	-0.78600600	1.82701200

3a
E(wB97xD) = -721.982197
Sum of electronic and thermal Free Energies = -721.828518
Charge = 0 Multiplicity = 1 NIMAG = 0

C	0.23612000	0.06937900	-0.10660100
C	-0.50428100	1.24530100	0.04338500
C	-1.89121900	1.19428300	0.10642100
C	-2.51450800	-0.04407600	0.01602700
C	-1.80073600	-1.22942000	-0.13441200
C	-0.41702100	-1.16484000	-0.19570200
H	-0.00013200	2.20197400	0.11176500
H	-2.47654500	2.09783400	0.22295400
H	-2.31660500	-2.17905000	-0.20155200
H	0.16413600	-2.07251300	-0.31233000
C	1.71126700	0.11581600	-0.17314900
N	-3.97859500	-0.10342100	0.08287200
O	-4.51592100	-1.19997200	-0.00545300
O	-4.59231300	0.94665900	0.22393700
O	2.26988900	1.34272900	-0.09167200
N	2.47787200	-0.89346000	-0.30203900
C	3.85944500	-0.38790300	-0.32517700
C	3.70137600	1.13759200	-0.12619400
H	4.10790000	1.73362400	-0.94470800
H	4.11275700	1.48848000	0.82463900
C	4.71212600	-1.05722900	0.74418700
H	4.75929200	-2.13731800	0.57800200
H	5.73196700	-0.65928200	0.72052000
H	4.29113700	-0.87549100	1.73898400
H	4.28250900	-0.60884300	-1.31131200
